# Supplementary material for: Effect of interventions incorporating personalised cancer risk information on intentions and behaviour: a systematic review and meta-analysis of randomised controlled trials
Source: BMJ Open. 2018 Jan 23;8(1):e017717. doi: 10.1136/bmjopen-2017-017717 (PMC5786113; doi:10.1136/bmjopen-2017-017717)
Supplement: Supplementary file 1 [file bmjopen-2017-017717supp001.pdf]

Supplementary file 1 – Complete search strategy

Medline and Cinahl

S28	S26 NOT S27
S27	review
S26	S24 AND S25
S25	S13 NOT S15
S24	S14 OR S16 OR S17 OR S21 OR S22 OR S23
S23	(behaviour OR behavior) AND health
S22	(MH "Health Behavior+") OR (MH "Risk Reduction Behavior+")
S21	S18 OR S20
S20	S19 AND S1
S19	screen* AND uptake OR attendance OR intention OR adherence
S18	(MM "Early Detection of Cancer/UT")
S17	anxiety* OR worry* OR denial* OR hopelessness* OR avoidance*
S16	efficacy OR effectiv*
S15	PT review OR PT letter OR PT comment OR PT editorial
S14	percep* OR perceive* OR understand* OR understood* OR accura* OR comprehen*
S13	S9 NOT S12
S12	S10 OR S11
S11	(MH "Prognosis+")
S10	prognos* OR treatment* OR surgery*
S9	S1 AND S8
S8	S6 OR S7
S7	(MH "Risk Assessment+")
S6	S4 AND S5
S5	score* OR model* OR predict* OR tool*
S4	S2 OR S3
S3	(MH "Risk+")
S2	risk*
S1	"cancer" OR (MH "Neoplasms+")

Embase

1	cancer.mp. or exp neoplasm/
2	exp risk/ or risk*.mp.
3	(score* or model* or predict* or tool*).mp. [mp=title, abstract, heading word, drug trade name, original title, device manufacturer, drug manufacturer, device trade name, keyword]
4	2 and 3
5	exp risk assessment/
6	4 or 5
7	1 and 6
8	(percep* or perceive* or understand* or understood* or accura* or comprehen*).mp. [mp=title, abstract, heading word, drug trade name, original title, device manufacturer, drug manufacturer, device trade name, keyword]
9	(efficacy* or effectiv*).mp. [mp=title, abstract, heading word, drug trade name, original title, device manufacturer, drug manufacturer, device trade name, keyword]
10	exp prognosis/
11	(prognos* or treatment* or surgery*).mp. [mp=title, abstract, heading word, drug trade name, original title, device manufacturer, drug manufacturer, device trade name, keyword]

12 (review or letter or comment or editorial).pt.
13 (radiotherapy* or stage* or grade*).mp. [mp=title, abstract, heading word, drug trade name, original title, device manufacturer, drug manufacturer, device trade name, keyword]
14 (anxiety* or worry* or fatalism* or hopelessness* or denial* or avoid*).mp. [mp=title, abstract, heading word, drug trade name, original title, device manufacturer, drug manufacturer, device trade name, keyword]
15 8 or 9 or 14
16 10 or 11 or 12 or 13
17 exp cancer screening/
18 health behaviour.mp. or exp health behavior/
19 ((behaviour or behavior) and health).mp. [mp=title, abstract, heading word, drug trade name, original title, device manufacturer, drug manufacturer, device trade name, keyword]
20 (screen* and (uptake or attendance or intention or adherence)).mp. [mp=title, abstract, heading word, drug trade name, original title, device manufacturer, drug manufacturer, device trade name, keyword]
21 20 and 1
22 15 or 17 or 18 or 19 or 21
23 22 and 7
24 23 not 16
25 limit 24 to yr="2000 -Current"
26 25 not review.mp. [mp=title, abstract, heading word, drug trade name, original title, device manufacturer, drug manufacturer, device trade name, keyword]

PsycInfo

S20 S19 NOT review Limiters - Publication Year: 2000-2015
S19 S17 NOT (S10 OR S11 OR S12)
S18 S17 NOT (S10 OR S11 OR S12)
S17 S7 and (S8 or S9 or S13 or S15 or S16)
S16 health AND (behaviour OR behavior)
S15 S14 AND S1
S14 screen* AND (uptake OR attendance OR intention OR adherence)
S13 MM "Cancer Screening"
S12 (prognos* OR treatment* OR surgery*) AND (S10 OR S11)
S11 prognos* OR treatment* OR surgery*
S10 DE "Prognosis"
S9 efficacy or effectiv* or worry* or anxiety* or hopelessness* or denial*
S8 percep* OR perceive* OR understand* OR understood* OR accura* OR comprehen*
S7 (S1 AND S6)
S6 (S4 OR S5)
S5 DE "Risk Assessment"
S4 (S2 AND S3)
S3 score* OR model* OR predict* OR tool*
S2 risk*
S1 DE "Neoplasms" OR DE "Benign Neoplasms" OR DE "Breast Neoplasms" OR DE "Endocrine Neoplasms" OR DE "Leukemias" OR DE "Nervous System Neoplasms" OR DE "Terminal Cancer"
