# Supplementary material for: Effect of interventions incorporating personalised cancer risk information on intentions and behaviour: a systematic review and meta-analysis of randomised controlled trials
Source: BMJ Open. 2018 Jan 23;8(1):e017717. doi: 10.1136/bmjopen-2017-017717 (PMC5786113; doi:10.1136/bmjopen-2017-017717)
Supplement: Supplementary file 2 [file bmjopen-2017-017717supp002.pdf]

Supplementary file 2. Quality assessment of included studies

Author, date	Study addressed a clearly focused issue	Randomisation	Recruitment / comparability of study groups at baseline	Blinding	Exposure measurement	Outcome measurement	Comparability of study groups during study	Follow up	Overall
Bodurtha, 2009	⬤	⬤	●	•	⬤	⬤	●	●	M-H
Bowen 2006	⬤	⬤	⬤	•	⬤	⬤	⬤	●	H
Bowen 2010	⬤	⬤	⬤	•	⬤	⬤	⬤	●	H
Davis, 2004	⬤	⬤	●	•	⬤	⬤	●	•	M
Glanz, 2013	⬤	⬤	●	●	●	●	●	⬤	M
Glazebrook 2006	⬤	⬤	●	•	●	●	●	•	M
Greene, 2003	●	●	•	•	●	●	•	•	L-M
Helmes, 2006	⬤	●	●	•	●	●	●	●	M
Holloway, 2003	⬤	⬤	⬤	●	⬤	⬤	●	•	M-H
Lipkus , 2006	⬤	⬤	•	•	●	●	•	⬤	M
Lipkus, 2001	⬤	●	•	•	n/a	●	●	●	M
Rimer 2002	⬤	⬤	●	•	●	⬤	●	●	M
Rubenstein, 2011	⬤	⬤	⬤	•	⬤	●	⬤	⬤	M
Schroy, 2011	⬤	⬤	⬤	•	⬤	⬤	⬤	●	M-H
Schroy, 2012	⬤	⬤	⬤	•	⬤	⬤	⬤	⬤	H
Seitz 2016	●	●	●	•	●	●	●	•	M

Sequist 2011	● (M)	● (M)	● (M)	• (L)	● (M)	● (M)	● (M)	n/a	M
Sherratt 2016	⬤ (H)	⬤ (H)	⬤ (H)	● (M)	● (M)	● (M)	⬤ (H)	⬤ (H)	H
Trevena 2008	● (M)	● (M)	⬤ (H)	⬤ (H)	● (M)	● (M)	● (M)	● (M)	M

• Low (L) ● Medium (M) ⬤ High (H)
