# Supplementary material for: Effect of interventions incorporating personalised cancer risk information on intentions and behaviour: a systematic review and meta-analysis of randomised controlled trials
Source: BMJ Open. 2018 Jan 23;8(1):e017717. doi: 10.1136/bmjopen-2017-017717 (PMC5786113; doi:10.1136/bmjopen-2017-017717)
Supplement: Author's manuscript [file bmjopen-2017-017717.draft_revisions.pdf]

BMJ Open is committed to open peer review. As part of this commitment we make the peer review history of every article we publish publicly available.

When an article is published we post the peer reviewers' comments and the authors' responses online. We also post the versions of the paper that were used during peer review. These are the versions that the peer review comments apply to.

The versions of the paper that follow are the versions that were submitted during the peer review process. They are not the versions of record or the final published versions. They should not be cited or distributed as the published version of this manuscript.

BMJ Open is an open access journal and the full, final, typeset and author-corrected version of record of the manuscript is available on our site with no access controls, subscription charges or pay-per-view fees (http://bmjopen.bmj.com).

If you have any questions on BMJ Open's open peer review process please email editorial.bmjopen@bmj.com

BMJ Open

The impact of cancer risk based interventions to people at population level risk: a systematic review and meta-analysis

Journal:	*BMJ Open*
Manuscript ID	bmjopen-2017-017717
Article Type:	Research
Date Submitted by the Author:	11-May-2017
Complete List of Authors:	Usher-Smith, Juliet; The Primary Care Unit, Institute of Public Health<br>Silarova, Barbora; MRC Epidemiology Unit,<br>Sharp, Stephen; University of Cambridge, MRC Epidemiology Unit<br>Mills, Katie; The Primary Care Unit, Institute of Public Health<br>Griffin, Simon; The Primary Care Unit, Institute of Public Health
**Primary Subject Heading**:	Communication
Secondary Subject Heading:	Oncology
Keywords:	ONCOLOGY, PREVENTIVE MEDICINE, PUBLIC HEALTH

SCHOLARONE™
Manuscripts

The impact of cancer risk based interventions to people at population level risk: a systematic review and meta-analysis

Juliet A Usher-Smith[1], Barbora Silarova[2], Stephen J Sharp[2], Katie Mills[1], Simon J Griffin[1]

[1] The Primary Care Unit, Institute of Public Health, University of Cambridge School of Clinical Medicine, Box 113 Cambridge Biomedical Campus, Cambridge, CB2 0SR, UK

[2] MRC Epidemiology Unit, Institute of Metabolic Science, Cambridge, CB2 0QQ

Correspondence to: Juliet Usher-Smith jau20@medschl.cam.ac.uk

For peer review only

For peer review only - http://bmjopen.bmj.com/site/about/guidelines.xhtml

ABSTRACT

Objective To provide a comprehensive review of the impact of interventions incorporating cancer risk information targeted at the general adult population.

Design A systematic review and random effects meta-analysis

Data sources An electronic search of Medline, EMBASE, CINAHL and PsychINFO from 01/01/2000 to 01/12/2015.

Inclusions criteria Primary research papers evaluating interventions including provision of a personal estimate of future cancer risk based on two or more non-genetic variables to adults recruited from the general population.

Results We included 32 studies reporting on 21 outcomes. Risk-based interventions reduce perceived absolute risk (standardised difference in means (95%CI) between groups: -0.46 (-0.67 to -0.26)) and perceived comparative risk (-0.73 (-1.03 to -0.43)), increase accuracy of absolute risk but not comparative risk, and reduce cancer worry (-0.44 (-0.58 to -0.29)), while not affecting intention to attend or attendance at screening (RR 1.00 (0.97-1.03)). Few studies reported the impact on health behaviours.

Conclusions Whilst there is evidence that cancer risk-based interventions decrease perceived risk and worry, they have no effect on screening behaviour and there is no evidence of effectiveness on health behaviours. Further research is needed before cancer risk information is incorporated into routine practice for health promotion in the general population.

Key words: Cancer, risk, systematic review, intervention, prevention, communication

For peer review only - http://bmjopen.bmj.com/site/about/guidelines.xhtml

Strengths and limitations of this study

- This systematic review is the first comprehensive review of the impact of cancer risk-based interventions on individuals at population level risk for cancer.
- The use of a broad search strategy across multiple databases enabled us to identify 32 studies reporting the impact of cancer risk-based interventions on 21 outcomes.
- However, there was large heterogeneity across the studies and the different outcome measures included. This limited the pooling of results.

For peer review only

For peer review only - http://bmjopen.bmj.com/site/about/guidelines.xhtml

INTRODUCTION

In 2006 the National Cancer Institute recognised risk prediction models as an 'area of extraordinary opportunity'[1]. Since then an increasing number of risk prediction models have been developed. Such models can facilitate a personalised approach to cancer prevention and treatment and a more equitable and cost-effective distribution of finite resources by targeting screening and prevention activities at those most likely to benefit. Furthermore, being able to estimate, communicate and monitor individual risk and demonstrate the impact of lifestyle change on future risk of cancer may complement wider collective approaches to shifting population distributions of behaviour, risk factors and cancer risk.

Research has shown that many individuals have incorrect perceptions of their risk of cancer[2–4] and that both over- and under-estimation are associated with maladaptive health behaviours[5]. Additionally, whilst up to 40% of all cancers are attributable to lifestyle factors[6], only 3% of people are aware that being overweight can increase their risk of cancer and less than a third that physical activity could help reduce risk[7–10], with one in seven people believing that lifetime risk of cancer is unmodifiable[11]. Providing individuals with estimates of their risk of cancer may improve accuracy of risk perception and motivate behaviour change at an individual level. It may also enable individuals to make more informed decisions around uptake of cancer screening programmes. This has led to an increasing number of interventions incorporating risk information being developed. All such interventions, however, have the potential to also cause harm both directly through reductions in psychological well-being and indirectly through false reassurance.

Information about risk of cardiovascular disease is now routinely offered to individuals, albeit with limited evidence of positive effects[12]. Understanding the impact of cancer risk based

For peer review only - http://bmjopen.bmj.com/site/about/guidelines.xhtml

interventions, before they are introduced into routine practice, is therefore important. Previous systematic reviews in this area have focused on randomised controlled trials in primary care[13], tailored information about cancer risk and screening[14,15], or educational interventions for people with cancer or at high risk of cancer[16]. We aimed to provide a comprehensive review of the impact of provision of cancer risk-based interventions to the general adult population across all settings.

METHODS

We performed a systematic literature review following an a priori established study protocol (available on request). Reporting followed the PRISMA statement[17].

Search strategy

We performed an electronic literature search of Medline, EMBASE, CINAHL and PsychINFO from January 2000 until December 2015 with no language limits using a combination of subject headings and free text incorporating 'cancer', 'risk/risk factor/risk assessment' and 'prediction/model/score/tool' and outcomes including 'perception', 'efficacy', 'anxiety', 'worry' and 'denial' (see Supplementary file 1 for the complete search strategies). We then extended the search by manually screening the reference lists of all included papers.

Study selection

We included studies if they were randomised controlled studies or pre-post intervention studies published as a primary research paper in a peer-reviewed journal, included adults with no previous history of cancer and included provision of a personal estimate of future cancer risk based on two or more non-genetic variables to individuals. In order to focus on the provision of cancer risk to the general population, we excluded studies which had recruited participants on

For peer review only

For peer review only - http://bmjopen.bmj.com/site/about/guidelines.xhtml

the basis of a personal or family history of cancer or following referral to specialist cancer risk services. Vignette, observational and qualitative studies were also excluded along with conference abstracts, editorials, commentaries and letters.

Two reviewers (JUS and BS) screened the titles and abstracts to exclude papers that were clearly not relevant. A third reviewer (SG) independently assessed a random selection of 5% of the papers screened by each of the first reviewers. The full text was examined if a definite decision to exclude could not be made based on title and abstract alone. Two reviewers (JUS and BS) independently assessed all full-text papers. We discussed papers for which it was unclear whether or not the inclusion criteria were met at consensus meetings with a third reviewer (SG). Papers written in languages other than English were translated into English for assessment and subsequent data extraction.

Data extraction

Two researchers (JUS+BS/KM) independently extracted data from studies included in the review using a standardized data abstraction form to reduce bias. The data extracted included: (1) Study characteristics (cancer type, study design, study setting, duration of follow-up); (2) selection of participants (inclusion criteria, method of recruitment/randomisation); (3) participant characteristics (age, level of cancer risk, sample size); (4) the intervention (risk tool used, method and format of risk communication, additional information or follow-up provided), and (4) measured outcome(s). Reviewers were not blinded to publication details.

Quality assessment

We conducted quality assessment at the same time as data extraction using a checklist based on the CASP guidelines[18] as an initial framework. Each study was then classified as high, medium

For peer review only - http://bmjopen.bmj.com/site/about/guidelines.xhtml

or low quality. No studies were excluded based on quality alone.

Data synthesis and statistical analysis

For analysis, we grouped the measured outcomes into those relating to: 1) risk perception and understanding of risk estimate; 2) psychological well-being (e.g. worry, anxiety, depression); 3) intention or motivation to change health-related behaviour; 4) intention to attend cancer screening; 5) change in health-related behaviour; and 6) cancer screening uptake. For continuous outcomes, the majority of the studies did not include sufficient data for us to express the effect of the intervention as a difference in the mean change from baseline between groups. We, therefore, present the standardised difference in mean values between groups at follow-up i.e. the difference in means expressed in standard deviation units. Where the standard deviation at follow-up was not reported, we used the standard deviation of the control group at baseline or the standard deviation from another study which measured the same outcome. For binary outcomes, such as screening attendance, we presented intervention effects as relative risk rather than odds ratios to avoid overestimating the risk[19]. Where possible we combined results from different studies using random effects meta-analysis but due to variations in study design and reporting we were only able to do this for a small number of outcomes. For outcomes with data from three or more studies, we estimated the heterogeneity between studies using the I^2 statistic. We did not perform formal tests of heterogeneity for outcomes with data from less than three studies. All analyses were conducted using statistical software package STATA/SE version 12.

RESULTS

After duplicates were removed, the search identified 30,879 papers. Of these, 30,711 were excluded at title and abstract level and a further 142 after full-text assessment. After title and

For peer review only - http://bmjopen.bmj.com/site/about/guidelines.xhtml

abstract screening by the first reviewers (JUS and BS), no additional papers met the inclusion criteria in the random 5% screened by the second reviewer (SG). The most common reasons for exclusion at full-text level were that the papers did not include provision of a personal risk estimate, were conference abstracts, recruited participants following referral to specialist genetic services, or did not include any data on predefined outcomes (Figure 1). Six further papers were identified through citation searching, giving 32 included studies in the analysis.

A summary of the design and setting of those 32 studies is shown in Table 1. Further details of the risk tool used to calculate the risk estimate provided to participants and the format of the intervention(s) are given in Table 2. With the exception of two studies in the UK[20,21] and one in the Netherlands[22], all studies were conducted in the USA. Fifteen provided information about risk of breast cancer, eight for colorectal cancer, three skin cancer, one each for lung and cervical cancer and four for multiple cancers. Quality assessment for each of studies is provided in Supplementary file 2. Eight were assessed as high or medium/high quality, 15 as medium quality and 9 as medium/low or low quality.

Together, the 32 studies reported the impact of cancer risk-based interventions on 21 outcomes. The overall findings for these along with the number of studies addressing each outcome are summarised in Table 3.

Risk perception and understanding of risk estimate

Perceived risk and accuracy of risk perception were the most frequent outcomes reported with 18 studies including a measure of one or both.

Perceived risk

For peer review only - http://bmjopen.bmj.com/site/about/guidelines.xhtml

Five randomised controlled trials (RCTs) measured either absolute risk perception (a numerical estimate of the individual's risk of developing cancer over a given time period) or comparative risk perception (an estimate of the individual's risk of developing cancer compared to others of the same age and sex) and included sufficient data for meta-analysis (Figure 2)[23–28]. In all five studies, on average, before provision of cancer risk information, participants overestimated both their absolute and comparative risk. The mean perceived absolute and comparative risk post intervention were significantly lower in those provided with personalised risk information than the control groups (standardised mean difference between groups: -0.46 (95%CI: -0.67 to -0.26, I^2 = 66%) for perceived absolute risk and -0.73 (95%CI: -1.03 to -0.43, I^2 = 0%) for perceived comparative risk). There were no clear differences according to format of the risk information or time between the intervention and outcome assessment.

We could not include a further seven studies in the meta-analysis. Two compared two intervention groups which received either absolute and comparative risk or comparative risk alone and found no significant changes in comparative risk perception from baseline to follow-up and no significant between-group differences[21,29]. An RCT by Dillard *et al.* only recruited women who overestimated their risk at baseline and compared effect of different styles of risk information. The overall estimate of lifetime risk across all groups decreased from 56.4% to 28.4% post-intervention (*n*=72) but the post-intervention levels remained significantly higher than the estimated risk (mean 11.2% difference) p<0.01[30]. By comparison Wang *et al.*[31] reported only on those who underestimated their risk at baseline. At the 6 month follow-up, perceptions about risk of colon cancer increased among a greater percentage of those in the intervention than in the control arm (17% vs 10%, p=0.05), but not for breast cancer or ovarian cancer. Female college students who completed a self-assessment risk score also reported

For peer review only - http://bmjopen.bmj.com/site/about/guidelines.xhtml

increased perceived comparative susceptibility ($p<0.05$) post-intervention compared with those who did not[32].

Two RCTs by Lipkus *et al.*[27,33] tested the effect of providing absolute risk feedback alone, comparative risk feedback alone or absolute plus comparative risk information. In one study, women given absolute risk feedback alone had lower perceptions of their numerical 10-year risks and comparative risk at follow up (16.8% (SD: 20.2) and 2.2 (SD: 0.8) respectively) than women who also received comparative risk information (26.1% (SD: 23.4) and 2.8 (SD: 0.9), $p<0.05$)[33]. In the other, perceptions of absolute risk did not vary significantly between groups but those informed that they had more than the average number of risk factors compared with others had higher mean comparative risk estimates than those in the control and in the lower comparative risk feedback groups[27].

Accuracy of risk perception

Six RCTs reported accuracy of risk perception with and without provision of risk information. It was possible to pool data from four studies that measured accuracy of absolute or comparative risk perception after provision of either absolute risk information or absolute plus comparative risk information[34–37]. Those who received risk estimates had more accurate absolute risk estimates at follow-up (RR 5.54 (1.84 to 16.67) I^2=86.5%), with no difference between those provided with absolute risk alone or absolute plus comparative risk, while there was no significant effect on comparative risk accuracy (RR 1.32 (0.82 to 2.13) I^2=78.2%). A further study which could not be pooled also showed an increase in the proportion who had accurate absolute and comparative risk estimates from baseline to follow-up (75 (25%) to 147 (49%) for accurate absolute risk estimates and 88 (29%) to 138 (46%) for accurate comparative risk)[38]. By contrast, one study showed no difference in the change in percentage of individuals

For peer review only - http://bmjopen.bmj.com/site/about/guidelines.xhtml

overestimating their absolute risk (-2.7% in the control group (*n*=184) compared to -5.8% in the intervention group (*n*=183), p=0.20)[39].

Two studies additionally compared the effect of alternative formats on risk accuracy. Emmons *et al.* showed that those who were randomised to have the opportunity to see how adopting or changing any of the risk factors would impact on their total risk profile had greater improvement in accuracy for both comparative and absolute risk accuracy compared to those who did not[36]. Lipkus *et al.* 2001a presented risk of breast cancer as either a point estimate on a 0-100% scale, as a range, or as a point estimate plus a range and showed no difference between groups in the percentage of participants who were accurate immediately after receiving risk information (point estimate 90.7%, point estimate plus range 97.7%, range 87.2-90.2%)[40].

Psychological well-being

Cancer worry

Ten RCTs reported cancer worry. Three reported worry in the different groups before and after the intervention using either the Lerman four item cancer worry scale[41], which ranges from 4 to 16[26,28], or a 10-point scale[24], and were able to be summarised as the standardised difference in mean worry between the intervention and control groups post intervention (Figure 3). The meta-analysis shows an overall reduction in worry with a standardised difference in means of -0.44 (95%CI: -0.58 to -0.29, I^2 = 0%).

Of the other seven RCTs which could not be pooled, six reported no significant intervention effects and four reported no numerical results[30,33,36,38]. Three reported no change in the proportion "*very concerned*" from baseline to follow up among controls (22.3% vs 22.0%, *n*=655) compared with a non-significant decrease among intervention women (27.1% vs

For peer review only - http://bmjopen.bmj.com/site/about/guidelines.xhtml

24.2%)[34], and no significant differences in the change from pre- to post- intervention scores on an adapted 3-item cancer worry scale with scores ranging from 3-12 (-0.17 for the intervention group vs -0.24 for the control group, p=0.65)[39] or index of overall negative emotions about getting colorectal cancer (CRC) on a scale from 3 to 15[27].

Anxiety and depression

Two studies measured anxiety and depression. Holloway *et al.*[20] included five modified Likert scales assessing screening-related anxiety and concerns alongside the Spielberger State Anxiety Inventory (SSAI)[42]. Women in intervention practices were significantly less likely to be "anxious about recent smear test" (OR: 0.81 (95%CI: 0.66 to 0.98)), "concerned about chances of serious problems with smear test in the future" (OR: 0.70 (95%CI: 0.51 to 0.95)), "fearful of cervical cancer" (OR: 0.66 (95%CI: 0.47 to 0.93)) and have a poor "perception of gynaecological health" (OR: 0.43 (95%CI: 0.19 to 0.99)). They were also less likely to be "concerned about smear result" but this was not statistically significant (OR: 0.75 (95%CI: 0.45 to 1.24)). After adjusting for clustering there was a non-statistically significant difference between the groups in the SSAI (-1.6 (95%CI: -3.5 to 0.2), p=0.084). The same study also included 20 additional outcomes relating to general aspects of knowledge and psychosocial wellbeing. No effect was seen for any of those relating to psychosocial wellbeing. The RCT by Trevena *et al.*, also reported no significant difference in anxiety (p=0.56)[43].

Affect and health-related quality of life

Affect was measured using the Positive and Negative Affect Scale (PANAS)[44] in one RCT in which the intervention group of female undergraduates received a risk feedback sheet whilst the control group received no information[30]. No significant between-group differences were observed. Health-related quality of life was measured in two RCTs[28,45] using the SF-36[46]. Both

For peer review only - http://bmjopen.bmj.com/site/about/guidelines.xhtml

reported a significant increase at follow-up in the intervention group compared with the control group.

Preferences and intentions for screening

Concordance between screening preferences and national recommendations

Two studies reported concordance between screening preferences and national recommendations for cervical screening[20] and lung cancer[47], both showed an increase in the intervention group. In the cluster-randomised trial by Holloway *et al.*[20] participants in the intervention group were significantly less likely to state a preference for the next screening interval to be 12 months or less (OR: 0.51 (95%CI: 0.41-0.64)). In the pre/post study in the US among a convenience sample of current or former smokers by Lau *et al.*[47] there was a significant increase in those with preferences in line with the U.S. Preventive Services Task Force recommendations from 25% to 59% (p<0.001), particularly amongst those ineligible for screening where concordance increased from 14% to 53% (p<0.001).

Decisional conflict

Two studies also reported a reduction in decisional conflict following risk information: the before-and-after study by Lau *et al.*[47] showed a significant decrease from 46.3 (SD: 29.7) to 15.1 (SD: 25.8) assessed using the ten-item Decisional Conflict Scale; and Lipkus *et al.*[27] showed that participants who received either absolute or absolute plus comparative risk had significantly lower ambivalence than those in the control group.

Intention to attend cancer screening

Eight studies included intentions to attend cancer screening, four for mammography and four for CRC screening. Seven showed no effect of risk information. Bodurtha *et al.*[48] found no

For peer review only - http://bmjopen.bmj.com/site/about/guidelines.xhtml

significant differences between the groups at 18 months after adjusting for baseline intentions and recruitment site (adjusted OR: 0.97 (95%CI: 0.70 to 1.33)). Davis *et al.*[39] reported that the intervention group were no more likely at one month to report being in the maintenance stage (having had one mammogram in the past two years and two or more in the past four years and planning to get another on schedule) than the control group who received no intervention (67% in the intervention group compared to 68% in the control group). Lipkus *et al.*[33] reported the extent to which the risk estimate affected intentions to get a mammogram on a 5-point scale from "*much less likely*" to "*much more likely*". Immediately after the risk information overall, 2.5%, 67.8%, and 24.8% reported that the risk feedback lowered, did not affect, or increased their intentions to get a mammogram respectively, with no differences between the groups. Helmes *et al*.[26] reported changes in a single breast health intentions measure which included intention to have mammography, clinical breast examination, and breast self-examination and found no significant differences at baseline (p=0.23) or three month follow-up (p=0.46). Schroy *et al.*[49] showed no difference between groups on a five-point scale of how sure they were that they would schedule a CRC screening test (mean scores 4.3 (SD: 1.0) for both groups). Han *et al* [50] also measured interest in CRC screening using a single five-point Likert response item. ANCOVA adjusting for sociodemographic factors only (age, race, sex) showed no significant change in interest in CRC screening following website use (change in interest = 0.08 (95%CI: 0.07–0.23), p =0.31), and no significant effects of age, race, or sex. Trevena *et al.*[43] similarly reported no effect on intention to have CRC screening of a decision aid including baseline risk. The only study to show an effect was an RCT by Lipkus *et al.*[27]. Intention was measured on a seven-point Likert scale as the extent to which participants intended to complete a faecal occult blood test (FOBT) that would be given to them within the following month. The intentions reported by participants who received absolute risk (mean 3.65, *n*=40) or absolute plus either low (mean 6.43, *n*=38) or high (mean 6.65, *n*=39)

For peer review only - http://bmjopen.bmj.com/site/about/guidelines.xhtml

comparative risk information were statistically significantly higher ($p<0.05$) than the control group (mean 2.21, n=43). The mean intention reported by the group which received the comparative risk was also significantly higher than for the absolute risk only group.

Attendance at screening

Twelve RCTs reported attendance at screening: six for mammography[28,37,39,45,48,51]; five for colorectal cancer[27,43,49,51,52]; and one for cervical cancer[20]. All showed no effect of the risk-based interventions and meta-analysis (Figure 4) confirmed this with a combined RR of 1.02 (95%CI: 0.98-1.03, I^2: 61.6%). A further cohort study which could not be included in those pooled results reported the number of women adhering to the American Cancer Society Guidelines for mammography before and after a risk based consultation with a pharmacist[53]. No significant differences were seen after the intervention in any of the age groups or those at higher risk.

Intention to change health-related behaviours

Smoking cessation

One cohort study[54] measured readiness to quit smoking over time after provision of personalised cancer risk information. Including only those with data at all three time points, the readiness to quit increased between baseline and one year ($p<0.0001$) and two years ($p<0.001$).

Intention to tan or protect skin

One RCT measured intention to tan on a six-item Likert-type scale and intention to protect skin using a three-item scale[32]. Participants who completed a self-assessment risk score reported significantly decreased intentions to use tanning beds (2.68, n=70 compared to 3.19, n=71,

For peer review only - http://bmjopen.bmj.com/site/about/guidelines.xhtml

$p<0.05$). In contrast there were no significant differences in intentions to protect skin (2.38, *n*=70 compared to 2.49, *n*=71, $p>0.05$).

Change in health-related behaviours

Sun exposure and sun protection habits

Two RCTs[21,55] measured sun protection habits by survey completion at baseline and follow up. Together these showed increases in overall sun protection habits with variable results for individual aspects including wearing a sun hat, wearing a shirt, wearing sunglasses, use of sun cream, number of sunburns, staying in the shade, and sun exposure during weekdays and weekends.

Tanning bed usage

One RCT[32] measured tanning behaviour change and tanning bed usage following provision of risk information. Participants who completed a self-assessment risk score reported lower rates of tanning bed usage in the previous month at follow-up (2.18, *n*=70 compared to 3.76, *n*=71, $p<0.05$) but no difference in change in tanning behaviour from pre- to post-intervention (-1.25, *n*=70 compared to -2.08, *n*=71, $p>0.05$).

Self/parent and clinical skin examination

Two RCTs measured rates of skin examination in adults[21] or parents and children[55]. Both showed statistically significant increases among adults and parents receiving personalised risk information ($p<0.05$) while the increase in parents examining their children was not significant ($p=0.06$).

Smoking

For peer review only - http://bmjopen.bmj.com/site/about/guidelines.xhtml

One cohort study[54] measured change in tobacco use and smoking status after providing personalised cancer risk information describing both modifiable and non-modifiable risk factors. Including only those with data at all three time points, the prevalence of current smokers increased from baseline to one year (5.7% to 6.7%, p<0.05) but decreased from baseline to follow up at two years (5.7% to 5.3%, p<0.05).

Clinical breast examination and breast self-examination

Three RCTs[28,45,48] and one pre-post intervention study[53] measured rates of clinical breast examination and/or breast self-examination after risk information. In the RCT by Bodurtha *et al.*, no significant differences were seen between the intervention and control group for either frequency of clinical breast examination (crude rates: 91.4% vs 91.0%; adjusted OR: 1.00 (95%CI: 0.60 to 1.66)) or breast self-examination (crude rates: 56.8% vs 57.6%; adjusted OR: 0.95 (95%CI: 0.67 to 1.33)[48]. The other three studies showed significant increases: Giles *et al.* showed that adherence to the American Cancer Society guidelines for monthly breast self-examination increased from 31% to 56% (p<0.001) for all women six months after the intervention and adherence to guidelines for clinical breast examination increased in women aged 40-49 years (81% to 97%, p<0.025)[53]; the two studies by Bowen *et al.*, found significantly (p<0.01) greater increases in the proportion reporting performing breast self-examination in the intervention groups (35% to 52% and 36% to 62%) compared with controls (33% to 36% and 38% to 40%)[28,45].

DISCUSSION

This systematic review is, to our knowledge, the first comprehensive review of the impact of cancer risk-based interventions on individuals at population level risk for cancer. The findings show that before receiving risk information, on average, people over-estimate their risk of

For peer review only - http://bmjopen.bmj.com/site/about/guidelines.xhtml

cancer – in some cases by a factor of three. Providing risk-based interventions reduces perceived risk, increases accuracy of absolute risk but not comparative risk, and reduces cancer worry, whilst not affecting intention to attend or attendance at screening. Risk-based interventions also increase self-report sun protection habits and skin examination and may decrease smoking but there is a notable absence of studies assessing the impact on diet, physical activity or alcohol consumption and none including objective measures of behaviour.

The finding that people tend to overestimate their risk and that provision of risk-based information on average reduces risk perception has been reported for other diseases, including diabetes[56], coronary heart disease[57] and cardiovascular disease[12]. Whilst this reduction in perceived risk may reduce maladaptive behaviours such as avoidance or denial[5], there is also the possibility that, instead of promoting healthy lifestyles, provision of disease risk information may provide false reassurance and encourage the adoption of unhealthy behaviours.

However, risk perception is not as simple as recalling a number or comparative estimate and conceptual problems in understanding risk information are well known[58]. Qualitative studies have also shown that an individual's risk perception is based on a complex integration of cognitive and social biases[59] arising from personal or lay theories of disease and risk[24,33,60] and past experiences, expectations and beliefs[61]. This may in part explain our finding that risk-based inventions improve accuracy of absolute risk perception but not comparative risk. By its very nature comparative risk is a more emotive construct and one which may be more prone to cognitive and social biases and in turn more resistant to change. For the same reasons, however, comparative risk may play a more important role in influencing decisions concerning health behaviours.

For peer review only - http://bmjopen.bmj.com/site/about/guidelines.xhtml

Our finding that risk-based interventions had no effect on intention to attend or attendance at screening is consistent with a previous Cochrane review in which personalised risk communication had little effect on the uptake of screening tests (fixed-effect OR 0.95 (95% CI 0.78 to 1.15))[15]. However, as in that review, there was evidence of decreased decisional-conflict and increased concordance between screening preferences and recommendations. This suggests that providing individuals with risk-based information may contribute to their decision to take up screening or not but is unlikely to influence overall rates of screening.

The main strengths of this review are the systematic search of multiple electronic databases and the broad inclusion criteria. Together these allowed us to include studies that assess the impact of cancer risk-based interventions on multiple outcomes. We have, therefore, been able to provide the first comprehensive overview of the impact of cancer risk-based intervention on individuals at population level risk. This approach, however, has its limitations. Firstly, there was large heterogeneity between the studies and in many the intervention consisted of provision of a risk score plus a range of additional information, either written or delivered in person or in groups. Separating the effect of the risk information alone from these additional elements of the interventions was therefore not possible. Secondly, although we have included 21 outcomes reported across the included studies, as a result of this number of outcomes, we were not able to assess and report all the interactions and moderators and mediators. Instead we have presented the overall effects that can be expected if risk information were to be provided to those at population level risk. Thirdly, as many of the included studies did not include sufficient data for us to express the results of continuous measures as the difference in the standardised mean change between groups, we have only been able to present the difference in mean values at follow-up. Finally, the heterogeneity remained high for several of the outcomes.

For peer review only - http://bmjopen.bmj.com/site/about/guidelines.xhtml

This likely reflects underlying variations in the design of the included studies and the different components included within the interventions but we feel our pooling of the data is justified in order to provide overall estimates reflecting the inherent variations in intervention delivery outside trial settings.

In addition to these specific limitations of our review, the findings also suggest a number of areas for future research. In particular, the absence of studies assessing the impact on diet, physical activity and alcohol consumption demonstrate the need for trials incorporating change in these behaviours, preferably measured objectively. Only with such data will we be able to assess whether the observed impacts on risk perception and accuracy translate into meaningful changes in risk factors and whether such individualised approaches have a place alongside population-wide prevention strategies.

Overall, this review demonstrates that whilst a large number of cancer risk prediction models exist and their incorporation into interventions does decrease perceived risk and worry and increase absolute risk accuracy, there is evidence that they have a minimal effect on screening behaviour and no evidence of their effectiveness on health behaviours. Further research is therefore needed before cancer risk information is incorporated into routine practice for those at population level risk of cancer.

ACKNOWLEDGEMENTS

The authors thank Isla Kuhn, Reader Services Librarian, University of Cambridge Medical Library, for her help developing the search strategy.

Contributors

For peer review only - http://bmjopen.bmj.com/site/about/guidelines.xhtml

JUS developed the protocol, completed the search, screened articles for inclusion, extracted data, synthesized the findings, interpreted the results and drafted the manuscript. BS developed the protocol, screened articles for inclusion, extracted data, interpreted the results and critically revised the manuscript. SS synthesized the findings and critically revised the manuscript. KM extracted data, interpreted the results and critically revised the manuscript. SJG developed the protocol, screened articles for inclusion, interpreted the results and critically revised the manuscript. All authors approved the final version.

Funding

JUS was funded by a National Institute for Health Clinical Lectureship and, along with KM, is now funded by a Cancer Research UK/BUPA Foundation Cancer Prevention Fellowship (C55650/A21464). BS was supported by the Medical Research Council [MC_UU_12015/4].

All researchers were independent of the funding body and the funder had no role in data collection, analysis and interpretation of data; in the writing of the report; or decision to submit the article for publication.

Data sharing

All data are available from the reports or authors of the primary research. No additional data is available.

Competing Interests

All authors have completed the Unified Competing Interest form at *www.icmje.org/coi_disclosure.pdf* (available on request from the corresponding author) and declare that (1) they have no support from or relationships with companies that might have an interest in the submitted work in the previous 3 years; (2) their spouses, partners, or children

For peer review only - http://bmjopen.bmj.com/site/about/guidelines.xhtml

have no financial relationships that may be relevant to the submitted work; and (3) they have no non-financial interests that may be relevant to the submitted work.

The corresponding author has the right to grant on behalf of all authors and does grant on behalf of all authors, an exclusive licence on a worldwide basis to the BMJ Publishing Group Ltd and its Licensees to permit this article (if accepted) to be published in BMJ editions and any other BMJPGL products and sub-licences to exploit all subsidiary rights, as set out in our licence (http://resources.bmj.com/bmj/authors/checklists-forms/licence-for-publication).

All authors had full access to all of the data in the study and can take responsibility for the integrity of the data and the accuracy of the data analysis

The corresponding author affirms that the manuscript is an honest, accurate, and transparent account of the study being reported; that no important aspects of the study have been omitted; and that any discrepancies from the study as planned (and, if relevant, registered) have been explained.

FIGURE LEGENDS

Figure 1. PRISMA flow diagram

Figure 2. Standardised difference in mean perceived absolute and comparative between groups post intervention. AR – absolute risk; CR – comparative risk

Figure 3. Standardised difference in mean worry between groups post intervention. AR – absolute risk; CR – comparative risk

Figure 4. Relative risk for adherence to recommended screening post intervention. CRC – colorectal cancer; FOBT – faecal occult blood test; AR – absolute risk; CR – comparative risk

REFERENCES

For peer review only - http://bmjopen.bmj.com/site/about/guidelines.xhtml

1 National Cancer Institute. The nation's investment in cancer research. A plan and budget proposal for the fiscal year 2006. 2006.

2 McCaul KD, O'Donnell SM. Naive beliefs about breast cancer risk. *Women's Heal* 1998;**4**:93–101.

3 Emery J, Morris H, Goodchild R, *et al.* The GRAIDS Trial: a cluster randomised controlled trial of computer decision support for the management of familial cancer risk in primary care. *Br J Cancer* 2007;**97**:486–93.

4 Weinstein ND, Atwood K, Puleo E, *et al.* Colon Cancer: Risk Perceptions and Risk Communication. *J Health Commun* 2004;**9**:53–65.

5 Waters EA, Klein WM, Moser RP, *et al.* Correlates of unrealistic risk beliefs in a nationally representative sample. *J Behav Med* 2011;**34**:225–35.

6 Parkin DM, Boyd L, Walker LC. The fraction of cancer attributable to lifestyle and environmental factors in the UK in 2010. *Br J Cancer* 2011;**105 Suppl**:S77-81.

7 Cancer Research UK. Perceptions of Risk Survey 2008 : Key Findings. 2008.

8 Grunfeld EA, Ramirez AJ, Hunter MS, *et al.* Women's knowledge and beliefs regarding breast cancer. *Br J Cancer* 2002;**86**:1373–8.

9 Redeker C, Wardle J, Wilder D, *et al.* The launch of Cancer Research UK's 'Reduce the Risk' campaign: baseline measurements of public awareness of cancer risk factors in 2004. *Eur J Cancer* 2009;**45**:827–36.

10 Wardle J, Waller J, Brunswick N, *et al.* Awareness of risk factors for cancer among British adults. *Public Health* 2001;**115**:173–4.

11 Ryan AM, Cushen S, Schellekens H, *et al.* Poor Awareness of Risk Factors for Cancer in Irish Adults: Results of a Large Survey and Review of the Literature. *Oncologist* 2015;**20**:372–8.

12 Usher-Smith JA, Silarova B, Schuit E, *et al.* Impact of provision of cardiovascular

For peer review only

For peer review only - http://bmjopen.bmj.com/site/about/guidelines.xhtml

disease risk estimates to healthcare professionals and patients: a systematic review. *BMJ Open* 2015;**5**:e008717.

13 Walker JG, Licqurish S, Pirotta M, *et al.* Cancer Risk Assessment Tools in Primary Care: A Systematic Review of Randomized Controlled Trials. 2015;:480–9.

14 Albada A, Ausems MGEM, Bensing JM, *et al.* Tailored information about cancer risk and screening: a systematic review. *Patient Educ Couns* 2009;**77**:155–71.

15 Edwards AGK, Evans R, Dundon J, *et al.* Personal risk communication for informed decision making about taking screening tests. *Cochrane Database Syst Rev 2006* Published Online First: 2006. doi:10.1002/14651858.CD001865.pub2

16 Dieng M, Watts CG, Kasparian NA, *et al.* Improving subjective perception of personal cancer risk: systematic review and meta-analysis of educational interventions for people with cancer or at high risk of cancer. *Psychooncology* 2014;**23**:613–25.

17 Moher D, Liberati A, Tetzlaff J, *et al.* Preferred reporting items for systematic reviews and meta-analyses: the PRISMA statement. *Int J Surg* 2010;**8**:336–41.

18 Critical Appraisal Skills Programme (CASP) Checklists. http://www.casp-uk.net/casp-tools-checklists

19 Knol MJ, Le Cessie S, Algra A, *et al.* Overestimation of risk ratios by odds ratios in trials and cohort studies: Alternatives to logistic regression. *CMAJ* 2012;**184**:895–9.

20 Holloway RM, Wilkinson C, Peters TJ, *et al.* Cluster-randomised trial of risk communication to enhance informed uptake of cervical screening. *Br J Gen Pract* 2003;**53**:620–5.

21 Glazebrook C, Garrud P, Avery A, *et al.* Impact of a multimedia intervention "Skinsafe" on patients' knowledge and protective behaviors. *Prev Med (Baltim)* 2006;**42**:449–54.

22 Timmermans DRM, Oudhoff JP. Weergave van risico's in de KWF Kanker Risico Test.

For peer review only

For peer review only - http://bmjopen.bmj.com/site/about/guidelines.xhtml

Ned Tijdschr Geneeskd 2012;**156**:A4888–910.

23 Quillin JM, Fries E, McClish D, *et al.* Gail model risk assessment and risk perceptions. *J Behav Med* 2004;**27**:205–14.

24 McCaul K, Canevello A, Mathwig J, *et al.* Risk communication and worry about breast cancer. *Psychol Health Med* 2003;**8**:379–89.

25 Lipkus IM, Klein WM, Rimer BK. Communicating breast cancer risks to women using different formats. *Cancer Epidemiol biomarkers Prev a Publ Am Assoc Cancer Res cosponsored by Am Soc Prev Oncol a Publ Am Assoc Cancer Res cosponsored by Ame* 2001;**10**:895–8.

26 Helmes AW, Culver JO, Bowen DJ. Results of a randomized study of telephone versus in-person breast cancer risk counseling. *Patient Educ Couns* 2006;**64**:96–103.

27 Lipkus IM, Klein WMP. Effects of communicating social comparison information on risk perceptions for colorectal cancer. *J Health Commun* 2006;**11**:391–407.

28 Bowen DJ, Powers D, Greenlee H. Effects of Breast Cancer Risk Counseling for Sexual Minority Women. *Health Care Women Int* 2006;**27**:59–74.

29 Braithwaite D, Sutton S, Mackay J, *et al.* Development of a risk assessment tool for women with a family history of breast cancer. *Cancer Detect Prev* 2005;**29**:433–9.

30 Dillard AJ, McCaul KD, Kelso PD, *et al.* Resisting good news: reactions to breast cancer risk communication. *Heal Commun* 2006;**19**:115–23.

31 Wang C, Sen A, Ruffin MT, *et al.* Family history assessment: Impact on disease risk perceptions. *Am J Prev Med* 2012;**43**:392–8.

32 Greene K, Brinn LS. Messages Influencing College Women's Tanning Bed Use: Statistical versus Narrative Evidence Format and a Self-Assessment to Increase Perceived Susceptibility. *J Health Commun* 2003;**8**:443.

33 Lipkus IM, Biradavolu M, Fenn K, *et al.* Informing women about their breast cancer

For peer review only

For peer review only - http://bmjopen.bmj.com/site/about/guidelines.xhtml

risks: truth and consequences. *Health Commun* 2001;**13**:205–26.

34 Livaudais-Toman J, Karliner LS, Tice JA, *et al.* Impact of a primary care based intervention on breast cancer knowledge, risk perception and concern: A randomized, controlled trial. *Breast* 2015;**24**:758–66.

35 Weinstein ND, Atwood K, Puleo E, *et al.* Colon Cancer: Risk Perceptions and Risk Communication. *J Health Commun* 2004;**9**:53–65.

36 Emmons K, Wong M, Puleo E, *et al.* Tailored Computer-Based Cancer Risk Communication: Correcting Colorectal Cancer Risk Perception. *J Health Commun* 2004;**9**:127–41.

37 Rimer BK, Halabi S, Sugg Skinner C, *et al.* Effects of a mammography decision-making intervention at 12 and 24 months. *Am J Prev Med* 2002;**22**:247–57.

38 Lipkus IM, Klein WMP, Skinner CS, *et al.* Breast cancer risk perceptions and breast cancer worry: what predicts what? *J Risk Res* 2005;**8**:439–52.

39 Davis S, Stewart S, Bloom J. Increasing the accuracy of perceived breast cancer risk: results from a randomized trial with Cancer Information Service callers. *Prev Med* 2004;**39**:64–73.

40 Lipkus IM, Klein WM, Rimer BK. Communicating breast cancer risks to women using different formats. *Cancer Epidemiol Biomarkers Prev* 2001;**10**:895–8.

41 Lerman C, Trock B, Rimer BK, *et al.* Psychological and behavioral implications of abnormal mammograms. *Ann Intern Med* 1991;**114**:657–61.

42 Speilberger C. *State-trait Anxiety Inventory for adults*. Palo Alto. CA: Consulting Psychologists Press 1983.

43 Trevena L, Irwig L, Barratt A, *et al.* Randomized trial of a self-administered decision aid for colorectal cancer screening. *J Med Screen* 2008;**15**:76–82.

44 Watson D, Clark LA, Tellegen A. Development and validation of brief measures of

For peer review only

For peer review only - http://bmjopen.bmj.com/site/about/guidelines.xhtml

positive and negative affect: the PANAS scales. *J Pers Soc Psychol* 1988;**54**:1063–70.

45 Bowen DJ, Powers D. Effects of a mail and telephone intervention on breast health behaviors. *Health Educ Behav* 2010;**37**:479–89.

46 Ware JE, Sherbourne CD. The MOS 36-item short-form health survey (SF-36). I. Conceptual framework and item selection. *Med Care* 1992;**30**:473–83.

47 Lau YK, Caverly TJ, Cao P, *et al.* Evaluation of a Personalized, Web-Based Decision Aid for Lung Cancer Screening. *Am J Prev Med* 2015;**49**:e125–9.

48 Bodurtha J, Quillin JMJ, Tracy K a, *et al.* Mammography screening after risk-tailored messages: the women improving screening through education and risk assessment (WISER) randomized, controlled trial. *J women's Heal* 2009;**18**:41–7.

49 Schroy PC, Emmons KM, Peters E, *et al.* Aid-assisted decision making and colorectal cancer screening: A randomized controlled trial. *Am J Prev Med* 2012;**43**:573–83.

50 Han PKJ, Duarte CW, Daggett S, *et al.* Effects of personalized colorectal cancer risk information on laypersons' interest in colorectal cancer screening: The importance of individual differences. *Patient Educ Couns* 2015;**98**:1280–6.

51 Rubinstein WS, Acheson LS, Neill SMO, *et al.* Clinical utility of family history for cancer screening and referral in primary care: A report from the Family Healthware Impact Trial. 2012;**13**:956–65.

52 Sequist TD, Zaslavsky AM, Colditz GA, *et al.* Electronic patient messages to promote colorectal cancer screening: a randomized controlled trial. *Arch Intern Med* 2011;**171**:636–41.

53 Giles JT, Kennedy DT, Dunn EC, *et al.* Results of a community pharmacy-based breast cancer risk-assessment and education program. *Pharmacotherapy* 2001;**21**:243–53.

54 Schnoll RA, Wang H, Miller SM, *et al.* Change in worksite smoking behavior following cancer risk feedback: A pilot study. *Am J Health Behav* 2005;**29**:215–27.

For peer review only

For peer review only - http://bmjopen.bmj.com/site/about/guidelines.xhtml

55 Glanz K, Steffen AD, Schoenfeld E, *et al.* Randomized Trial of Tailored Skin Cancer Prevention for Children: The Project SCAPE Family Study. *J Health Commun* 2013;**18**:1368–83.

56 Godino JG, van Sluijs EMF, Marteau TM, *et al.* Lifestyle Advice Combined with Personalized Estimates of Genetic or Phenotypic Risk of Type 2 Diabetes, and Objectively Measured Physical Activity: A Randomized Controlled Trial. *PLOS Med* 2016;**13**:e1002185.

57 Asimakopoulou KG, Fox C, Spimpolo J, *et al.* The impact of different time frames of risk communication on Type 2 diabetes patients' understanding and memory for risk of coronary heart disease and stroke. *Diabet Med* 2008;**25**:811–7.

58 Han PK, Lehman TC, Massett H, *et al.* Conceptual problems in laypersons' understanding of individualized cancer risk: a qualitative study. *Heal Expect* 2009;**12**:4–17.

59 Bottorff JL, Ratner PA, Johnson JL, *et al.* Communicating cancer risk information: the challenges of uncertainty. *Patient Educ Couns* 1998;**33**:67–81.

60 Heiniger L, Butow PN, Charles M, *et al.* Intuition versus cognition: a qualitative exploration of how women understand and manage their increased breast cancer risk. *J Behav Med* Published Online First: 2015. doi:10.1007/s10865-015-9632-7

61 Walter FM, Emery J. Perceptions of family history across common diseases: a qualitative study in primary care. *Fam Pract* 2006;**23**:472–80.

For peer review only - http://bmjopen.bmj.com/site/about/guidelines.xhtml

Table 1. Details of the design, setting and key outcomes of the included studies

Author, year	Cancer site(s)	Design	Follow-up	Setting and participants	Risk level / co-morbidities	Outcome(s)	Quality *
Bodurtha 2009	Breast	RCT	18 months	899 women with no history of breast cancer recruited from waiting rooms of four women's health clinics	Not given	Mammography, clinical breast examinations, breast self-examination, mammography intentions	M-H
Bowen 2006	Breast	RCT	6 and 24 months	150 sexual minority women recruited via public advertisements	Mean Gail lifetime risk 12%	Perceived risk, cancer worry, mental health, breast self-examination, breast cancer screening	H
Bowen 2010	Breast	RCT	12 months	1,366 women recruited via telephone with no previous diagnosis of breast cancer	Mean Gail lifetime risk 12%	Quality of life, breast self-examination, mammography	
Davis, 2004	Breast	RCT	1 month	392 women with no history cancer calling the Cancer Information Service	27% 2-6% lifetime risk; 32% 6-9% lifetime risk; 41% 9-46% lifetime risk	Adherence to breast cancer screening, intention for breast cancer screening, risk perception, cancer worry	M
Dillard, 2006a	Breast	RCT	0, 2 weeks	Convenience sample of 72 female undergraduates with no first degree relatives with breast cancer	Not given	Mood, comparative risk estimates, percentage risk estimates for other women, worry, beliefs about the accuracy of the feedback, seriousness ratings concerning breast cancer	L-M
Dillard, 2006b	Breast	RCT	0, 2 weeks	Convenience sample of 62 female undergraduates with no first degree relatives with breast cancer	Not given	Perceived risk	L-M
Emmons, 2004	Colorectal	RCT	0	353 patients with no history of cancer scheduled for routine or non-urgent health care visits to two primary care practices	Mean 20 year risk 9.96 per 1,000	Accuracy of risk perception, cancer worry	M-H
Giles 2001	Breast	Cohort	6 months	140 members of general public attending one of six community pharmacies	15% ≥ 1.7 lifetime risk	Breast self-examination, clinical breast examination, mammography screening	M
Glanz 2013	Skin	RCT	16 weeks	Convenience sample of 1047 parents not currently being treated for skin cancer recruited through schools and community centres	38% high risk	Sun protection habits, sun exposure, skin examination by parents	M
Glazebrook 2006	Skin	Cluster RCT	6 months	589 recruited from 10 primary care practices from a convenience sample of appointments	Not given	Sun protection habits, perceived risk	M
Greene 2003	Skin	RCT	3-4 weeks	141 undergraduates at one university	Not given	Perceived risk, intention to tan, actual tan bed usage	L-M
Han, 2015	Colon	Cohort	0	578 members of general public accessing freely	0.8-22% lifetime	Interest in getting tested or screened for	M

For peer review only - http://bmjopen.bmj.com/site/about/guidelines.xhtml

				accessible website "Are you at risk for colon cancer"	risk	colon cancer	
Helmes, 2006	Breast	RCT	3 months	Random sample of 340 members of state healthcare system with no history of breast/ovarian cancer or testing for cancer risk	Mean 9.5% (3.2) lifetime risk	Risk perception, cancer worry, intention to have mammogram and clinical breast examination, intention to do breast self-examination, interest in genetic testing	M
Holloway, 2003	Cervical	RCT	0, 4 years	1890 women attending routine cervical smear test at one of 29 GP practices	78-80% very low risk; 20-22% low risk	Preference for future screening interval, screening related anxiety, screening related mental health, actual screening behaviour, 21 short-term outcome measures relating to knowledge and psychosocial wellbeing	M-H
Kaplan 2014	Breast	RCT	1 week and 6 months	1235 patients scheduled for routine or non-urgent health care visits to two primary care practices with no history of breast cancer	75% average risk	Patient-physician discussion and documentation of breast cancer risk	L-M
Lau 2015	Lung	Cohort	0	Convenience sample of 60 current or former smokers with no history of lung cancer and who had not have a chest CT in the previous year	Mean 6-year risk 0.012%	Knowledge of cancer risk factors and lung cancer screening, decisional conflict, concordance	L-M
Lipkus 2006	Colorectal	RCT	0	160 members of general public with no history of CRC or screening for CRC recruited through newspaper advertisements	Not given	Absolute and comparative CRC risk, worry, defensive reactions, ambivalence, intention to screen using a FOBT, actual FOBT screening rates	M
Lipkus, 2001a	Breast	2x2 design	0, 6-8 months	169 members of general public recruited through newspaper advertisements	Mean lifetime risk 7.78% (SD 1.13)	Perception of risk	L
Lipkus, 2001b	Breast	RCT	0	121 members of general public recruited through newspaper advertisements	Mean 10 year risk 2.65% (SD 1.13)	Perception of risk, negative affect related to getting breast cancer, mammography screening and intentions	M
Lipkus, 2005	Breast	RCT	0	301 members of general public recruited through newspaper advertisements	Mean lifetime risk 8.5% (range 1.2 to 30.5)	Perception of risk, accuracy of risk, breast cancer worry	L
Livaudais-Toman, 2015	Breast	RCT	1 week	1235 women with scheduled appointments at an academic medical center or hospital with no history of breast cancer	25% high risk	Perception of risk, breast cancer concern	H
McCaul, 2003	Breast	2x2 design	0, 1-2 weeks	59 female undergraduates with no first-degree relatives with breast cancer at one university	Mean lifetime risk 11.5%	Perception of risk, accuracy of risk, breast cancer worry	L
Quillin, 2004	Breast	RCT	1 month	299 women with no history of breast cancer attending outpatient mammography clinic	Mean lifetime risk 11.1% (SD 5.14)	Perception of risk, risk accuracy	M

For peer review only - http://bmjopen.bmj.com/site/about/guidelines.xhtml

Rimer 2002	Breast	RCT	1 and 2 years	752 women aged 40-44 and 50-54 enrolled in a personal care plan	Mean 10 year risk 2.7%	Risk accuracy, mammography	M
Rubenstein 2011	Breast, ovarian, colon	RCT	6 months	3786 patients from primary care clinics with no history of colon, breast or ovaraian cancer invited by mail following record review	34% moderate or strong risk of ≥ 1 of the cancers	CRC screening, mammography	M
Schnoll, 2005	Lung, breast, colorectal, ovarian, skin, prostate	Cohort	1 and 2 years	6378 employees and their spouses from six worksites	Not given	Smoking status, readiness to quit smoking	M-H
Schroy, 2011	Colorectal	RCT	0	666 patients due for bowel screening identified from monthly audits of one hospital's electronic medical record	Average	Knowledge, preferences, satisfaction with the decision-making process, screening intentions, and test concordance	M-H
Schroy, 2012	Colorectal	RCT	0, 1, 3, 6 and 12 months	825 patients due for bowel screening identified from monthly audits of one hospital's electronic medical record	Average	Completion of a CRC screening test	H
Sequist 2012	Colorectal	RCT	1 and 4 months	1,103 patients from 14 ambulatory health centres who were overdue for colorectal cancer screening	Average	CRC screening	M
Timmermans 2012	Colon, lung	RCT	0	612 members of general public with no history of cancer	4.6% reported a history of cancer	Risk accuracy	M
Trevena 2008	Colorectal	RCT	1 month	314 patients recruited from 6 primary care practices without a history of colorectal cancer	Not given	Anxiety, screening intentions, CRC screening	M
Wang, 2012	Colon, breast, ovarian	RCT	6 months	3786 patients from primary care clinics with no history of colon, breast or ovarian cancer invited by mail following record review	82% moderate or strong risk for ≥1 of the 6 conditions	Perception of risk	M
Weinstein, 2004	Colon	2x2 design	0	353 patients with no history of cancer with scheduled routine or non-urgent health care visits at two primary care practices	Below-average	Recall of risk communication, risk accuracy	L-M

RCT – randomised controlled trial; CRC – colorectal cancer; CT computerised tomography; FOBT – faecal occult blood test
* L – low, M – medium, H - high

For peer review only

Table 2. Details of the risk-based interventions in each of the included studies

Author, year	Risk tool	Intervention group(s)	Comparison (where applicable)	Format of risk
Bodurtha 2009	Gail model (5 year and lifetime)	Information sheets with risk level and handouts addressing traditional constructs of Health Belief Model including barriers to mammography, breast cancer seriousness, individual risk for breast cancer, and benefits of yearly mammography	General information about breast cancer prevention practices, including mammography	Usual (<15%), Moderate (15-30%) or Strong (>30%)
Bowen 2006	Gail model (5 year, 10 year and at age 79)	Four weekly 2-hour sessions led by a health counsellor focusing on risk assessment and education, screening, stress management and social support	Delayed intervention	No details given
Bowen 2010	Gail model (lifetime)	Information sheets with general information on breast cancer risk and personalised risk information plus telephone counselling and offer for more intensive group or genetic counselling	Delayed intervention	Bar graph of absolute lifetime risk along with age-appropriate estimates for the "average risk" woman
Davis, 2004	BRCA tool (updated version of Gail model) (lifetime)	10min brief intervention designed to increase accuracy of perceived risk including results of risk assessment and screening recommendations tailored to participant's stage of adoption of mammography and follow up written information	No intervention	Verbal over the telephone. No additional details given.
Dillard, 2006a	Gail model (5 year and lifetime)	Risk feedback sheet following completion of risk assessment questions plus kindness questionnaire or study calendar +/- additional questions about risk factors	No intervention	Absolute risk estimate as % and comparative estimate ranging from 'much lower' to 'much higher' along with a visual scale with risk estimate represented by a mark on the scale
Dillard, 2006b	Gail model (5 year and lifetime)	Risk feedback sheet including information on two other women and their risk factors as downward social comparison condition	Risk feedback sheet	Absolute risk estimate as % and comparative estimate ranging from 'much lower' to 'much higher' along with a visual scale with risk estimate represented by a mark on the scale +/- downward social comparison condition
Emmons, 2004	Harvard cancer risk model (20 year)	1) Absolute risk with active impact; 2) Absolute risk without active impact; 3) Absolute and relative risk with active impact; 4) Absolute and relative risk without active impact	Passive risk communication but no absolute or relative risk estimates	Absolute risk over 20 years +/- relative risk plus absolute risk +/- option to manipulate their risk factor profiles to see impact of changing risk factors on a visual scale using an interactive computer-based tool
Giles 2001	Gail model (5 year and lifetime)	Pharmacist consultation and written explanation of individual risk factors with 5 year probability, lifetime probability, comparison with someone of the same age with	Not applicable	Bar chart of absolute risk as a percentage for 5 year and lifetime risk alongside risk of a woman of the same age and race with no

For peer review only - http://bmjopen.bmj.com/site/about/guidelines.xhtml

		no additional risk factors along with encouragement to follow guidelines for breast self-examination and mammograms		additional risk factors
Glanz 2013	Children's BRAT	Three mailings with personalised risk feedback, interactive skin cancer education materials, a family fun guide and suggestions for overcoming barriers and reminders to engage in preventive practices	Single mailing of standardised skin cancer information	No details given
Glazebrooke 2006	No details given	Self-directed computer program including sections on skin protection, how to detect melanoma, dangers of sun exposure, how to check skin, how to reduce risk and individualized feedback of risk	Not applicable	Comparative risk
Greene 2003	Relative risk adapted from "ADD Wants to Convert"	Self-assessment of risk alongside generic messages about tanning, tanning beds and sun exposure	Generic messages about tanning, tanning beds and sun exposure	Numerical scale from 1-36
Han, 2015	CCRAT (NCI Colorectal Cancer Risk Assessment Tool) (5, 10 year and lifetime)	Individual's estimated CRC risk as well as age- and sex-matched population average CRC risk	Not applicable	Absolute 5-year, 10-year and lifetime risk on visual scale from 0-100% and pictogram with 100 people for individual and age- and sex-matched population average
Helmes, 2006	Gail model (lifetime)	Face-to-face or telephone intervention consisting of 8 items: 1) a personal risk sheet ; 2) a personal computer-drawn pedigree; 3) a 23 page participant booklet; 4) Breast self-examination brochure; 5) Pap smear and mammography brochure; 6) BSE shower card; 7) pictures of chromosomes and gene mutations; 8) a list of community resources for breast cancer	No intervention	Bar charts of absolute % risk with numerical % alongside for the individual, an average-risk woman, and a high-risk woman
Holloway, 2003	Wilkinson score	Brief 10 minute counselling session integrated with smear test appointment including relative and absolute risks and then negotiation of appropriate screening intervals	Normal care	Comparative and absolute risk in pictures and numbers
Kaplan 2014	Referral Screening Tool; Gail Model; and Breast Cancer Surveillance Consortium model (5 year)	Breast cancer risk assessment by tablet computer at the clinic that generated individually tailored printouts for patients and their physicians	Breast cancer risk assessment via telephone	High risk or average risk
Lau 2015	PLCOm2012 model	Web-based decision aid which computed baseline lung	Not applicable	Absolute risk as % and on visual scale plus

For peer review only - http://bmjopen.bmj.com/site/about/guidelines.xhtml

	(6 year)	cancer risk and an individual's chance of benefiting from, and risk of being harmed by, screening		pictogram of 100 people showing benefits of lung cancer screening and description of harms and benefits with numbers for each
Lipkus 2006	Not given	Written information about CRC, CRC screening methods and CRC risk factors plus either 1) tailored CRC risk factor information or 2) tailored CRC risk factor information plus information on whether their total number of CRC risk factors was greater or not than average	Written information about CRC, CRC screening methods, and CRC risk factors	Narrative comparative risk
Lipkus, 2001a	Gail model (lifetime)	1-2 page handout describing the Gail Model plus either 1) a point estimate of their risk; 2) a risk range derived from the 95% confidence intervals; 3) a point estimate of their risk plus a risk range derived from the 95% confidence intervals	No information	As a percentage in a pie chart
Lipkus, 2001b	Gail model (10 year)	1 page handout describing the Gail model plus absolute risk alone	As for intervention group plus how their risk compared to a woman of their age and race at the lowest level of risk	Absolute risk +/- risk of a woman at the lowest level of risk as percentages in a pie chart
Lipkus, 2005	Gail model (lifetime)	In three groups, women obtained information about their absolute risk only, in one of three formats. Three additional groups received their absolute risk in one of the three formats along with information about the risk of another woman the same age and race as the participant with no other risk factors	No information	Numerical percentages either 1) ''point estimate condition'' - single best point estimate of their risk as a percentage; 2) ''range condition'' - upper and lower bounds of risk as percentages; 3) "point estimate and range''
Livaudais-Toman, 2015	Referral Screening Tool; Gail Model; and Breast Cancer Surveillance Consortium model (5 year)	Individually-tailored print-outs for patients and their physicians (one page in length) including specific risk reduction recommendations.	No information	Absolute risk as a percentage and relative risk (higher/lower)
McCaul, 2003	Gail model (5 year and lifetime)	Printed feedback on two sheets including either absolute risk information, relative risk information, or both	No information	Absolute risk as a percentage and mark on two scales ranging from 0% to 100%. Comparative risk as a label (e.g., 'Same') and a mark on a scale ranging from 'Much lower' to 'Much higher,' with seven labels including a centre label of 'About the Same'

For peer review only - http://bmjopen.bmj.com/site/about/guidelines.xhtml

Quillin, 2004	Gail model (5 year and lifetime)	Risk assessment with genetic counsellor then one-page summary including breast health messages that were appropriate for their calculated risk, including recommendations for screening, available genetic counselling, and contact information for psychosocial support	No information	Percentage risk alongside qualitative interpretation ("low", "moderate", high") and whether it is higher/lower than the average women's risk
Rimer 2002	Gail model (10 year and lifetime)	Tailored print booklet and brief tailored newspaper plus personalized risk	Usual care (postcard reminder)	Absolute risk as a percentage
Rubenstein 2011	Family Healthware tool	Written personalized risk assessment and tailored prevention messages	Written generalized prevention messages	Qualitative risk - weak, moderate or strong familial risk
Schnoll, 2005	Not given	A personalized risk-feedback letter, which listed modifiable and non-modifiable cancer risk factors, calculated risk, and information about specialized risk-reduction programs.	Not applicable	Qualitative risk - above average or average
Schroy, 2011	Harvard cancer risk model (10 year)	Interactive 20-30 min computer-based decision aid plus personalized risk assessment	Interactive 20-30 min computer-based decision aid alone	Thermograph, indicating where the participant is along with a description e.g. your risk is below average
Schroy, 2012	Harvard cancer risk model (10 year)	Interactive 20-30 min computer-based decision aid plus personalized risk assessment followed immediately by a meeting with their providers to discuss screening and identify a preferred screening strategy. Providers received written notification hand-delivered by all the patients acknowledging that they were participating in the "CRC decision aid study" at the time of the visit to ensure that screening was discussed	As for intervention but without personalized risk assessment	Qualitative framing ("very much below average risk" to "very much above average risk") with accompanying suggestions for behaviour modifications that might reduce risk, including a strong recommendation for screening, regardless of risk
Sequist 2011	Harvard cancer risk model (10 year)	Personalized electronic message highlighting their overdue screening status and providing a link to a web-based tool to assess their risk	No contact	Comparative risk on 7-point ordinal scale from very-much below average to very-much above average and in interactive graphical format
Timmermans 2012	Shortened KWF Kanker Risico Test (5 year)	Participants were randomized to one of 12 experimental groups who received a combination of: 1) Average population risk (no quantitative risk information provided/only the number/number + graphic illustration); 2) the calculated personal risk (no quantitative information /numbers); and 3) the relative risk reduction after changing lifestyle (or no quantification of risk reduction)	Standard version of the KWF-KRT	12 different formats including numbers, graphical illustrations (emoticons and bar charts) of average population risk, personal risk and relative risk reduction

For peer review only - http://bmjopen.bmj.com/site/about/guidelines.xhtml

Trevena 2008	No details given	20 page booklet including personalized risk, absolute reduction in colorectal cancer mortality with screening over the next 10 years, probability of test outcomes from screening and information about how to get screeed.	3 page booklet with information and recommendations about screening	Words and 1000-face diagrams
Wang, 2012	Family Healthware tool	Written personalized prevention messages delivered via mail, e-mail, or in person tailored to familial risk for each of the six conditions alongside a family tree and information about the characteristics in one's family history that put the person at increased risk (if applicable)	Standard print messages about screening and lifestyle choices via mail, e-mail, or in-person	Qualitative risk - weak, moderate or strong familial risk
Weinstein, 2004	Harvard cancer risk model (20 year)	Absolute or relative risk electronically +/- the opportunity to manipulate the risk along with details of the risk factors that comprised their risk and recommendations for what they should change to reduce their risk	Feedback on which of their behaviours and non-modifiable attributes lowered and which increased their risk and advice on steps they could take to lower their risk	Absolute risk - numerical estimate in units of cases per thousand people like them alongside an oval window with the risk marked on a horizontal hairline. Comparative risk was expressed in terms of one of seven categories: ''very much below average'', ''much below average,'' ''below average,'' ''average'', ''above average,'' ''much above average,'' and ''very much above average'' alongside an oval window with the risk marked on a horizontal hairline

CRC – colorectal cancer

For peer review only

For peer review only - http://bmjopen.bmj.com/site/about/guidelines.xhtml

Table 3. Summary of impact of provision of personalised cancer risk on measured outcomes

	Decrease	No change	Increase
Risk perception	Perceived risk (*n*=12)		Absolute risk accuracy (*n*=5)
		----------Comparative risk accuracy (*n*=3) ----------	
Psychological outcomes	Worry (*n*=10)	Depression (*n*=2)<br>Affect (*n*=1)	Quality of life (*n*=2)
	----------------Anxiety (*n*=2)---------------		
Health behaviour	Intention to use tanning beds (*n*=1)<br>Smoking (*n*=1)	Intention to protect skin(*n*=1)<br>Clinical breast examination (*n*=2)<br>Use of tanning beds (*n*=1)	Readiness to quit smoking(*n*=1)<br>Sun protection habits (*n*=2)<br>Skin examination (*n*=2)<br>Breast self-examination (*n*=4)
Screening	Decisional conflict around screening decisions (*n*=2)	Intention to attend screening (*n*=8)<br>Attendance at screening (*n*=13)	Concordance between screening preferences and recommendations (*n*=2)

For peer review only - http://bmjopen.bmj.com/site/about/guidelines.xhtml

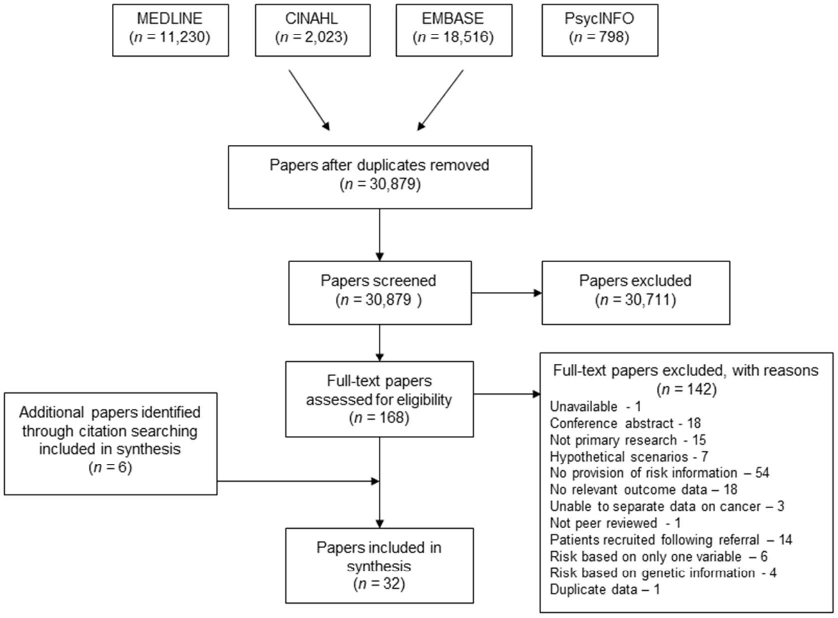

Figure 1. PRISMA flow diagram

254x190mm (96 x 96 DPI)

For peer review only - http://bmjopen.bmj.com/site/about/guidelines.xhtml

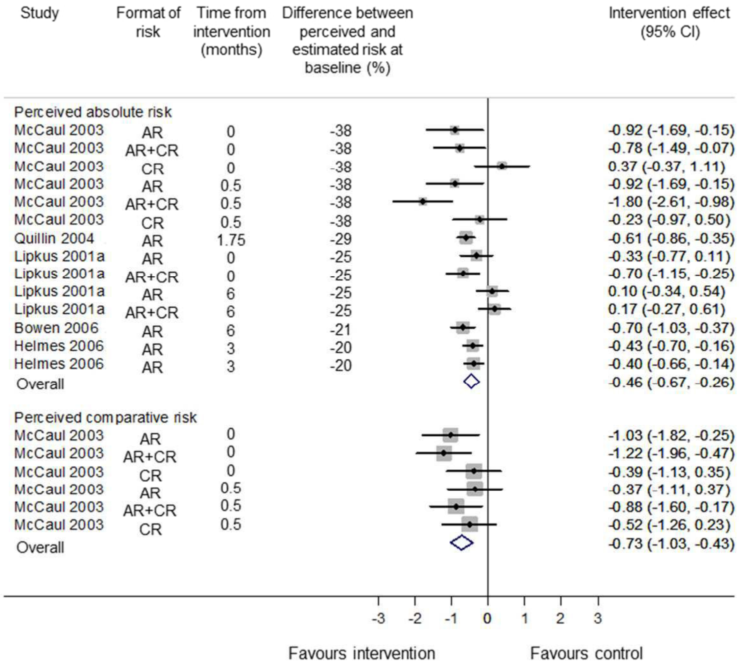

Figure 2. Standardised difference in mean perceived absolute and comparative between groups post intervention. AR – absolute risk; CR – comparative risk

254x190mm (96 x 96 DPI)

w only

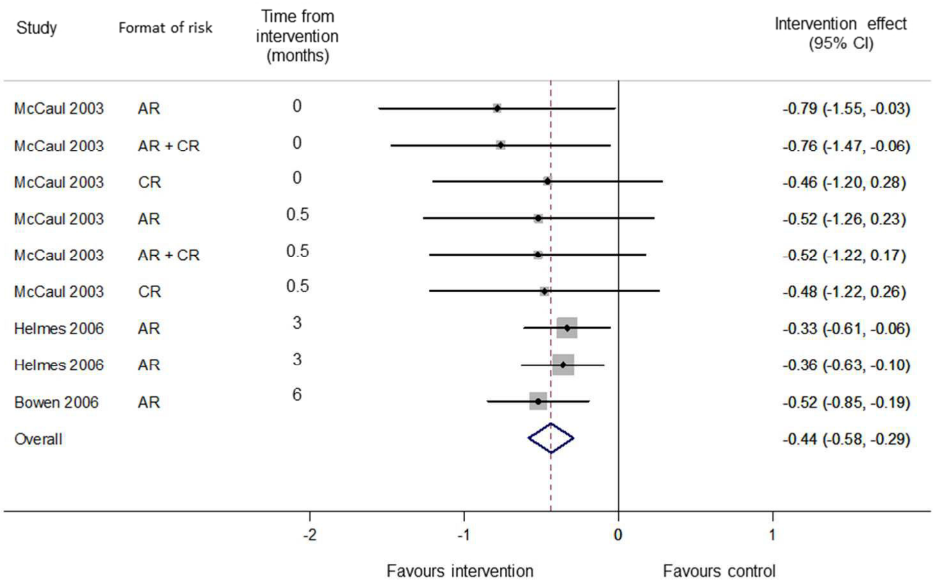

Figure 3. Standardised difference in mean worry between groups post intervention. AR – absolute risk; CR – comparative risk

254x190mm (96 x 96 DPI)

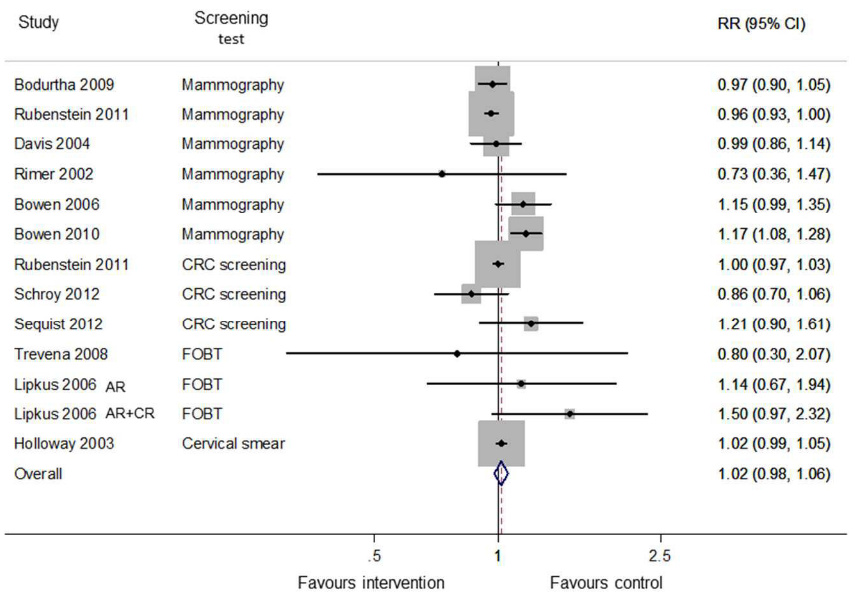

Figure 4. Relative risk for adherence to recommended screening post intervention. CRC – colorectal cancer; FOBT – faecal occult blood test; AR – absolute risk; CR – comparative risk

254x190mm (96 x 96 DPI)

w only

Supplementary file 1 – Complete search strategy

Medline and Cinahl

S28	S26 NOT S27
S27	review
S26	S24 AND S25
S25	S13 NOT S15
S24	S14 OR S16 OR S17 OR S21 OR S22 OR S23
S23	(behaviour OR behavior) AND health
S22	(MH "Health Behavior+") OR (MH "Risk Reduction Behavior+")
S21	S18 OR S20
S20	S19 AND S1
S19	screen* AND uptake OR attendance OR intention OR adherence
S18	(MM "Early Detection of Cancer/UT")
S17	anxiety* OR worry* OR denial* OR hopelessness* OR avoidance*
S16	efficacy OR effectiv*
S15	PT review OR PT letter OR PT comment OR PT editorial
S14	percep* OR perceive* OR understand* OR understood* OR accura* OR comprehen*
S13	S9 NOT S12
S12	S10 OR S11
S11	(MH "Prognosis+")
S10	prognos* OR treatment* OR surgery*
S9	S1 AND S8
S8	S6 OR S7
S7	(MH "Risk Assessment+")
S6	S4 AND S5
S5	score* OR model* OR predict* OR tool*
S4	S2 OR S3
S3	(MH "Risk+")
S2	risk*
S1	"cancer" OR (MH "Neoplasms+")

Embase

1	cancer.mp. or exp neoplasm/
2	exp risk/ or risk*.mp.
3	(score* or model* or predict* or tool*).mp. [mp=title, abstract, heading word, drug trade name, original title, device manufacturer, drug manufacturer, device trade name, keyword]
4	2 and 3
5	exp risk assessment/
6	4 or 5
7	1 and 6
8	(percep* or perceive* or understand* or understood* or accura* or comprehen*).mp. [mp=title, abstract, heading word, drug trade name, original title, device manufacturer, drug manufacturer, device trade name, keyword]
9	(efficacy* or effectiv*).mp. [mp=title, abstract, heading word, drug trade name, original title, device manufacturer, drug manufacturer, device trade name, keyword]
10	exp prognosis/
11	(prognos* or treatment* or surgery*).mp. [mp=title, abstract, heading word, drug trade name, original title, device manufacturer, drug manufacturer, device trade name, keyword]

For peer review only

12 (review or letter or comment or editorial).pt.
13 (radiotherapy* or stage* or grade*).mp. [mp=title, abstract, heading word, drug trade name, original title, device manufacturer, drug manufacturer, device trade name, keyword]
14 (anxiety* or worry* or fatalism* or hopelessness* or denial* or avoid*).mp. [mp=title, abstract, heading word, drug trade name, original title, device manufacturer, drug manufacturer, device trade name, keyword]
15 8 or 9 or 14
16 10 or 11 or 12 or 13
17 exp cancer screening/
18 health behaviour.mp. or exp health behavior/
19 ((behaviour or behavior) and health).mp. [mp=title, abstract, heading word, drug trade name, original title, device manufacturer, drug manufacturer, device trade name, keyword]
20 (screen* and (uptake or attendance or intention or adherence)).mp. [mp=title, abstract, heading word, drug trade name, original title, device manufacturer, drug manufacturer, device trade name, keyword]
21 20 and 1
22 15 or 17 or 18 or 19 or 21
23 22 and 7
24 23 not 16
25 limit 24 to yr="2000 -Current"
26 25 not review.mp. [mp=title, abstract, heading word, drug trade name, original title, device manufacturer, drug manufacturer, device trade name, keyword]

PsycInfo
S20 S19 NOT review Limiters - Publication Year: 2000-2015
S19 S17 NOT (S10 OR S11 OR S12)
S18 S17 NOT (S10 OR S11 OR S12)
S17 S7 and (S8 or S9 or S13 or S15 or S16)
S16 health AND (behaviour OR behavior)
S15 S14 AND S1
S14 screen* AND (uptake OR attendance OR intention OR adherence)
S13 MM "Cancer Screening"
S12 (prognos* OR treatment* OR surgery*) AND (S10 OR S11)
S11 prognos* OR treatment* OR surgery*
S10 DE "Prognosis"
S9 efficacy or effectiv* or worry* or anxiety* or hopelessness* or denial*
S8 percep* OR perceive* OR understand* OR understood* OR accura* OR comprehen*
S7 (S1 AND S6)
S6 (S4 OR S5)
S5 DE "Risk Assessment"
S4 (S2 AND S3)
S3 score* OR model* OR predict* OR tool*
S2 risk*
S1 DE "Neoplasms" OR DE "Benign Neoplasms" OR DE "Breast Neoplasms" OR DE "Endocrine Neoplasms" OR DE "Leukemias" OR DE "Nervous System Neoplasms" OR DE "Terminal Cancer"

For peer review only

Supplementary file 2. Quality assessment of included studies

Author, date	Study addressed a clearly focused issue	Use of an appropriate method / Randomisation (for RCTs)	Recruitment / comparability of study groups at baseline	Blinding (for RCTs)	Exposure measurement	Outcome measurement	Comparability of study groups during study (for RCTs)	Follow up (for longitudinal studies)	Confounding factors (for non-RCTs):	Overall
Bodurtha, 2009	●	●	•	·	●	●	•	•	n/a	M-H
Bowen 2006	●	●	●	·	●	●	●	•	n/a	H
Bowen 2010	●	●	●	·	●	●	●	•	n/a	H
Davis, 2004	●	●	•	·	●	●	•	·	n/a	M
Dillard, 2006a	•	•	·	·	•	•	·	•	n/a	L-M
Dillard, 2006b	•	•	·	·	•	•	·	•	n/a	L-M
Emmons, 2004	●	●	•	·	●	●	•	·	n/a	M-H
Giles, 2001	●	●	•	·	•	•	•	•	n/a	M
Glanz, 2013	●	●	•	•	•	•	•	●	n/a	M
Glazebrook 2006	●	●	•	·	•	•	•	·	n/a	M
Greene, 2003	•	•	·	·	•	•	·	·	n/a	L-M
Han, 2015	•	n/a	•	n/a	•	•	n/a	n/a	·	M
Helmes, 2006	●	•	•	·	•	•	•	•	n/a	M
Holloway, 2003	●	●	●	•	●	●	•	·	n/a	M-H
Kaplan, 2014	•	•	•	·	•	•	·	·	n/a	L-M
Lau, 2015	•	•	·	n/a	·	•	n/a	n/a	·	L-M

Lipkus , 2006	H	H	L	L	M	M	L	H	n/a	M
Lipkus, 2001a	H	H	L	L	M	M	L	L	L	L
Lipkus, 2001b	H	M	L	L	n/a	M	M	M	M	M
Lipkus, 2005	H	L	L	L	n/a	H	L	H	M	L
Livaudais-Toman, 2015	H	H	H	H	n/a	H	H	M	n/a	H
McCaul, 2003	H	H	L	L	H	M	L	M	n/a	L
Quillin, 2004	H	H	L	L	H	M	L	M	n/a	M
Rimer 2002	H	H	M	L	M	H	M	M	n/a	M
Rubenstein, 2011	H	H	H	L	H	M	H	H	n/a	M
Schnoll, 2005	H	H	L	n/a	M	M	L	L	H	M-H
Schroy, 2011	H	H	H	L	H	H	H	M	n/a	M-H
Schroy, 2012	H	H	H	L	H	H	H	H	n/a	H
Sequist 2011	M	M	M	L	M	M	M	n/a	n/a	M
Timmermans 2012	M	M	M	L	M	M	M	n/a	n/a	M
Trevena 2008	M	M	H	H	M	M	M	M	n/a	M
Wang, 2012	H	H	H	L	H	M	H	H	n/a	M
Weinstein, 2004	H	M	L	L	H	M	L	L	n/a	L-M

• Low (L) ● Medium (M) ⬤ High (H)

For peer review only

PRISMA 2009 Checklist

Section/topic	#	Checklist item	Reported on page #
**TITLE**			
Title	1	Identify the report as a systematic review, meta-analysis, or both.	1
**ABSTRACT**			
Structured summary	2	Provide a structured summary including, as applicable: background; objectives; data sources; study eligibility criteria, participants, and interventions; study appraisal and synthesis methods; results; limitations; conclusions and implications of key findings; systematic review registration number.	2
**INTRODUCTION**			
Rationale	3	Describe the rationale for the review in the context of what is already known.	3
Objectives	4	Provide an explicit statement of questions being addressed with reference to participants, interventions, comparisons, outcomes, and study design (PICOS).	4
**METHODS**			
Protocol and registration	5	Indicate if a review protocol exists, if and where it can be accessed (e.g., Web address), and, if available, provide registration information including registration number.	4
Eligibility criteria	6	Specify study characteristics (e.g., PICOS, length of follow-up) and report characteristics (e.g., years considered, language, publication status) used as criteria for eligibility, giving rationale.	5
Information sources	7	Describe all information sources (e.g., databases with dates of coverage, contact with study authors to identify additional studies) in the search and date last searched.	4
Search	8	Present full electronic search strategy for at least one database, including any limits used, such that it could be repeated.	Supplementary file 1
Study selection	9	State the process for selecting studies (i.e., screening, eligibility, included in systematic review, and, if applicable, included in the meta-analysis).	5
Data collection process	10	Describe method of data extraction from reports (e.g., piloted forms, independently, in duplicate) and any processes for obtaining and confirming data from investigators.	5/6
Data items	11	List and define all variables for which data were sought (e.g., PICOS, funding sources) and any assumptions and simplifications made.	5/6
Risk of bias in individual studies	12	Describe methods used for assessing risk of bias of individual studies (including specification of whether this was done at the study or outcome level), and how this information is to be used in any data synthesis.	6
Summary measures	13	State the principal summary measures (e.g., risk ratio, difference in means).	6/7
Synthesis of results	14	Describe the methods of handling data and combining results of studies, if done, including measures of consistency (e.g., I^2) for each meta-analysis.	6/7

For peer review only - http://bmjopen.bmj.com/site/about/guidelines.xhtml

PRISMA 2009 Checklist

Section/topic	#	Checklist item	Reported on page #
Risk of bias across studies	15	Specify any assessment of risk of bias that may affect the cumulative evidence (e.g., publication bias, selective reporting within studies).	6
Additional analyses	16	Describe methods of additional analyses (e.g., sensitivity or subgroup analyses, meta-regression), if done, indicating which were pre-specified.	N/A
**RESULTS**			
Study selection	17	Give numbers of studies screened, assessed for eligibility, and included in the review, with reasons for exclusions at each stage, ideally with a flow diagram.	7 and Figure 1
Study characteristics	18	For each study, present characteristics for which data were extracted (e.g., study size, PICOS, follow-up period) and provide the citations.	Table 1 and Table 2
Risk of bias within studies	19	Present data on risk of bias of each study and, if available, any outcome level assessment (see item 12).	Supplementary file 2
Results of individual studies	20	For all outcomes considered (benefits or harms), present, for each study: (a) simple summary data for each intervention group (b) effect estimates and confidence intervals, ideally with a forest plot.	8-17 and Figures 2, 3 and 4
Synthesis of results	21	Present results of each meta-analysis done, including confidence intervals and measures of consistency.	8-17 and Figures 2, 3 and 4
Risk of bias across studies	22	Present results of any assessment of risk of bias across studies (see Item 15).	N/A
Additional analysis	23	Give results of additional analyses, if done (e.g., sensitivity or subgroup analyses, meta-regression [see Item 16]).	N/A
**DISCUSSION**			
Summary of evidence	24	Summarize the main findings including the strength of evidence for each main outcome; consider their relevance to key groups (e.g., healthcare providers, users, and policy makers).	17/18
Limitations	25	Discuss limitations at study and outcome level (e.g., risk of bias), and at review-level (e.g., incomplete retrieval of identified research, reporting bias).	19/20
Conclusions	26	Provide a general interpretation of the results in the context of other evidence, and implications for future research.	18-20
**FUNDING**			

For peer review only

PRISMA 2009 Checklist

Funding	27	Describe sources of funding for the systematic review and other support (e.g., supply of data); role of funders for the systematic review.	21

From: Moher D, Liberati A, Tetzlaff J, Altman DG, The PRISMA Group (2009). Preferred Reporting Items for Systematic Reviews and Meta-Analyses: The PRISMA Statement. PLoS Med 6(6): e1000097. doi:10.1371/journal.pmed1000097

For more information, visit: **www.prisma-statement.org**.

For peer review only

BMJ Open

Change in intention and behaviour following interventions incorporating information about cancer risk amongst the general population: a systematic review and meta-analysis of randomised controlled trials

Journal:	*BMJ Open*
Manuscript ID	bmjopen-2017-017717.R1
Article Type:	Research
Date Submitted by the Author:	12-Oct-2017
Complete List of Authors:	Usher-Smith, Juliet; The Primary Care Unit, Institute of Public Health<br>Silarova, Barbora; MRC Epidemiology Unit,<br>Sharp, Stephen; University of Cambridge, MRC Epidemiology Unit<br>Mills, Katie; The Primary Care Unit, Institute of Public Health<br>Griffin, Simon; The Primary Care Unit, Institute of Public Health
**Primary Subject Heading**:	Communication
Secondary Subject Heading:	Oncology
Keywords:	ONCOLOGY, PREVENTIVE MEDICINE, PUBLIC HEALTH

SCHOLARONE™
Manuscripts

For peer review only

Change in intention and behaviour following interventions incorporating information about cancer risk amongst the general population: a systematic review and meta-analysis of randomised controlled trials

Juliet A Usher-Smith[1], Barbora Silarova[2], Stephen J Sharp[2], Katie Mills[1], Simon J Griffin[1]

[1] The Primary Care Unit, Institute of Public Health, University of Cambridge School of Clinical Medicine, Box 113 Cambridge Biomedical Campus, Cambridge, CB2 0SR, UK

[2] MRC Epidemiology Unit, Institute of Metabolic Science, Cambridge, CB2 0QQ

Correspondence to: Juliet Usher-Smith jau20@medschl.cam.ac.uk

For peer review only

For peer review only - http://bmjopen.bmj.com/site/about/guidelines.xhtml

ABSTRACT

Objective To provide a comprehensive review of the impact on intention and behaviour, including screening uptake, of interventions incorporating information about cancer risk targeted at the general adult population.

Design A systematic review and random effects meta-analysis

Data sources An electronic search of Medline, EMBASE, CINAHL and PsychINFO from 01/01/2000 to 01/07/2017.

Inclusions criteria Randomised controlled trials of interventions including provision of a personal estimate of future cancer risk based on two or more non-genetic variables to adults recruited from the general population including at least one behavioural outcome.

Results We included 19 studies reporting 12 outcomes. There was significant heterogeneity in interventions and outcomes between studies. There is evidence that interventions incorporating cancer risk information do not affect intention to attend or attendance at screening (Relative risk (RR) 1.00 (0.97-1.03)). There is limited evidence that they increase intention to tan, smoking abstinence, sun protection, adult skin self-examination and breast examination but do not increase intention to protect skin, smoking cessation or parental child skin examination. No studies reported changes in diet, alcohol consumption or physical activity.

Conclusions Interventions incorporating cancer risk information do not affect uptake of screening but there is limited evidence of effect on some health behaviours. Further research, ideally including objective measures of behaviour, is needed before cancer risk information is incorporated into routine practice for health promotion in the general population.

Key words: Cancer, risk, systematic review, intervention, prevention, communication

For peer review only - http://bmjopen.bmj.com/site/about/guidelines.xhtml

For peer review only

Strengths and limitations of this study

- This systematic review is the first comprehensive review of interventions incorporating cancer risk on intention and behaviour of individuals in the general population.
- The use of a broad search strategy across multiple databases enabled us to identify 19 randomised controlled trials reporting the impact of interventions incorporating cancer risk information on 12 outcomes.
- However, there was large heterogeneity across the studies, including the content of interventions and the outcome measures. This meant it was only possible to meta-analyse one outcome, attendance at screening, and in many studies separating the effect of the risk information alone from additional elements of the interventions was not possible.

For peer review only - http://bmjopen.bmj.com/site/about/guidelines.xhtml

INTRODUCTION

In 2006 the US National Cancer Institute recognised risk prediction models as an 'area of extraordinary opportunity'[1]. Since then an increasing number of risk prediction models have been developed. Such models can facilitate a personalised approach to cancer prevention and treatment and a more equitable and cost-effective distribution of finite resources by targeting screening and prevention activities at those most likely to benefit. Furthermore, being able to estimate, communicate and monitor individual risk and demonstrate the impact of lifestyle change on future risk of cancer may complement wider collective approaches to shifting population distributions of behaviour, risk factors and cancer risk.

Research has shown that many individuals have incorrect perceptions of their risk of cancer[2–4] and that both over- and under-estimation are associated with maladaptive health behaviours[5]. Additionally, whilst up to 40% of all cancers are attributable to lifestyle factors[6], only 3% of people are aware that being overweight can increase their risk of cancer and less than a third that physical activity could help reduce risk[7–10]. One in seven people additionally believe that lifetime risk of cancer is unmodifiable[11]. Most behaviour change theories suggest that perceived risk is important alongside other constructs such as self-efficacy, response efficacy in promoting behaviour change[12,13]. Providing individuals with estimates of their risk of cancer alongside other behaviour change interventions may therefore help motivate behaviour change at an individual level. It may also enable individuals to make more informed decisions about uptake of screening tests for cancer. This has led to the development of an increasing number of interventions incorporating information about cancer risk being developed.

Understanding the impact of interventions incorporating information about cancer risk on behaviour and intention to change behaviour before they are introduced into routine practice is

For peer review only - http://bmjopen.bmj.com/site/about/guidelines.xhtml

important. Previous systematic reviews in this area have focused only on trials in primary care[14] or tailored information about cancer risk and screening[15,16]. In this review we aimed to provide a comprehensive synthesis of the impact of interventions incorporating information about cancer risk on intention and behaviour within the general adult population.

METHODS

We performed a systematic literature review following an a priori established study protocol (available on request). Reporting followed the PRISMA statement[17].

Search strategy

We performed an electronic literature search of Medline, EMBASE, CINAHL and PsychINFO from January 2000 until July 2017 with no language limits using a combination of subject headings and free text incorporating 'cancer', 'risk/risk factor/risk assessment' and 'prediction/model/score/tool' (see Supplementary file 1 for the complete search strategies). We then extended the search by manually screening the reference lists of all included papers. We chose to begin the search in 2000 as the previous review of tailored information about cancer risk and screening had noted that computer delivered interventions, as would be required for calculating risk scores, were only described in publications from 2000 onwards[15].

Study selection

We included studies if they were randomised controlled studies published as a primary research paper in a peer-reviewed journal, included adults with no previous history of cancer and included provision to individuals of a personal estimate of future cancer risk based on two or more non-genetic variables and reported at least one behavioural outcome. In order to focus on the provision of cancer risk to the general population, we excluded studies which had

For peer review only

For peer review only - http://bmjopen.bmj.com/site/about/guidelines.xhtml

recruited participants on the basis of a personal or family history of cancer or following referral to specialist cancer risk services. Vignette, before-and-after studies without a control group, cross-sectional and qualitative studies were also excluded along with conference abstracts, editorials, commentaries and letters.

Two reviewers (JUS and BS) each screened half of the titles and abstracts to exclude papers that were clearly not relevant. A third reviewer (SG) independently assessed a random selection of 5% of the papers screened by each of the first reviewers. The full text was examined if a definite decision to exclude could not be made based on title and abstract alone. Two reviewers (JUS and BS) independently assessed all full-text papers. We discussed papers for which it was unclear whether or not the inclusion criteria were met at consensus meetings with a third reviewer (SG). Papers written in languages other than English were translated into English for assessment and subsequent data extraction.

Data extraction

Two researchers (JUS+BS/KM) independently extracted data from studies included in the review using a standardized data abstraction form to reduce bias. The data extracted included: (1) Study characteristics (cancer type, study design, study setting, duration of follow-up); (2) selection of participants (inclusion criteria, method of recruitment/randomisation); (3) participant characteristics (age, level of cancer risk, sample size); (4) the intervention (risk tool used, method and format of risk communication, additional information or follow-up provided), and (4) measured outcome(s). Reviewers were not blinded to publication details.

Quality assessment

We conducted quality assessment at the same time as data extraction using a checklist based on

For peer review only - http://bmjopen.bmj.com/site/about/guidelines.xhtml

the Critical Appraisal Skills Programme (CASP) guidelines[18] as an initial framework. This includes eight questions concerning whether the study addressed a clearly focused issue, the method of recruitment and randomisation, whether blinding was used, the measurement of the exposure and outcome, the comparability of the study groups and the follow-up. Each study was then classified as high, medium or low quality. No studies were excluded based on quality alone.

Data synthesis and statistical analysis

For analysis, we grouped the measured outcomes into those relating to: 1) preferences or intention to attend cancer screening; 2) cancer screening uptake; 3) intention or motivation to change health-related behaviour; and 4) change in health-related behaviour. It was only possible to pool results for screening attendance. For this we used random effects meta-analysis[19] and the 'metan' package in Stata. We present intervention effects as relative risk rather than odds ratios to avoid overestimating the risk[20]. We estimated the heterogeneity between studies using the I^2 statistic. All analyses were conducted using statistical software package Stata/SE version 12.

RESULTS

After duplicates were removed, the search identified 38,906 papers. Of these, 35,604 were excluded at title and abstract level and a further 183 after full-text assessment. After title and abstract screening by the first reviewers (JUS and BS), no additional papers met the inclusion criteria in the random 5% screened by the second reviewer (SG). The most common reasons for exclusion at full-text level were that the papers did not include provision of a personal risk estimate (*n*=62), did not include any data on predefined outcomes (*n*= 37), were conference

For peer review only - http://bmjopen.bmj.com/site/about/guidelines.xhtml

abstracts (*n*=20), or were not primary research (*n*=16) (Figure 1). Five further papers were identified through citation searching, giving 19 studies included in the analysis.

A summary of the participants and setting of those 19 studies is shown in Table 1. With the exception of three studies conducted in the UK[21–23], all studies took place in the USA. Most recruited participants from those attending primary care clinics (n=3), or from lists of potentially eligible individuals from electronic medical records (n=7), telephone services (n=1), insurance records (n=1) or survey companies (n=1). Two recruited through schools, community centres and universities, one from those calling a cancer information service and three used public advertisements.

In eight studies information was provided about risk of breast cancer, in five about risk of colorectal cancer, in three risk of skin cancer, one lung cancer, one cervical cancer and one multiple cancers. Further details of the risk models used to calculate the risk estimate provided to participants and the format of the intervention(s) are given in Table 2. All eight studies providing information about breast cancer risk used the Gail risk model[24]. This was the first risk model developed for breast cancer and includes age, age at menarche, age at first live birth, number of previous biopsies, number of biopsies showing atypical hyperplasia, and number of first-degree relatives with breast cancer. Where details were given (*n*=3), all studies on colorectal cancer used the Harvard Cancer Risk tool[25] which includes family history, height and weight, alcohol consumption, vegetable and red meat consumption, physical activity, screening history, a history of inflammatory bowel disease, and use of aspirin, folate and female hormones. Other risk models used were the Liverpool Lung Project model[26], Family Healthware tool[27], Wilkinson score for cervical cancer[28] and the brief skin cancer risk assessment tool (BRAT)[29] adapted for children. Quality assessment for each of studies is

For peer review only - http://bmjopen.bmj.com/site/about/guidelines.xhtml

provided in Supplementary file 2. Seven were assessed as high or medium/high quality, 11 as medium quality and one as medium/low.

Overall findings and evidence synthesis along with the number and quality of studies addressing each outcome are summarised in Table 3.

Preferences and intentions for screening

Preferences for screening

Two RCTs reported participants' views about screening. In the cluster-randomised trial by Holloway *et al.*[21] participants who received a 10 minute counselling session including information about relative and absolute risks of cervical cancer integrated within a smear test appointment were significantly less likely to state a preference for the next interval for cervical screening to be 12 months or less than those who received usual care (OR: 0.51 (95%CI: 0.41-0.64)). The second study by Lipkus *et al.*[30] reported attitudinal ambivalence towards faecal occult blood test (FOBT) screening measured by their agreement with three Likert-style items stating that they had “mixed feelings”, felt “torn” and had “conflicting thoughts” about whether to get screened for CRC using an FOBT. Participants who received estimates of either absolute or absolute plus comparative risk alongside written information about CRC screening had significantly lower ambivalence than those who received the same written information without tailored CRC risk information ($p<0.05$).

Intention to attend cancer screening

Eight studies assessed intentions to attend cancer screening: five for mammography and four for CRC screening. Five showed no effect of risk information, three in which the only substantial difference between the intervention and control groups was the provision of a risk

For peer review only - http://bmjopen.bmj.com/site/about/guidelines.xhtml

estimate[31–33]. Bodurtha *et al.*[31] found no significant differences at 18 months between those randomised to receive either printed sheets with their 5-year and lifetime estimates of breast cancer risk alongside information addressing barriers to mammography, breast cancer seriousness and benefits of yearly mammography, or general information about breast cancer prevention practices not tailored to their risk level (OR after adjusting for baseline intentions and recruitment site: 0.97 (95%CI: 0.70 to 1.33)). Davis *et al.*[34] reported that women who received a brief intervention over the telephone including information about lifetime risk of cancer and screening recommendations were no more likely at one month to report being in the maintenance stage (having had one mammogram in the past two years and two or more in the past four years and planning to get another on schedule) than the control group who received no intervention (67% in the intervention group compared to 68% in the control group). Helmes *et al*.[35] reported changes in a single breast health intentions measure which included intention to have mammography, clinical breast examination, and breast self-examination. They found no significant differences at baseline (p=0.23) or three month follow-up (p=0.46) between women who received estimates of their lifetime risk of breast cancer along with information about breast awareness either face-to-face or over the telephone and a control group who received no intervention. Schroy *et al*.[32] randomised participants to complete an interactive 20-30 minutes computer-based decision aid which either did or did not include a personalised risk assessment. There was no difference between groups on a five-point scale of how sure they were that they would schedule a CRC screening test (mean scores 4.3 (SD: 1.0) for both groups). Trevena *et al.*[33] similarly reported no effect on intention to have CRC screening of a 20-page decision aid including information about baseline risk and absolute reduction in CRC mortality with screening, compared to a 3-page booklet with information and recommendations about screening.

For peer review only - http://bmjopen.bmj.com/site/about/guidelines.xhtml

The two studies reporting an effect were by Lipkus *et al.*[30] and Seitz *et al.*[36]. In Lipkus *et al.* intention to complete an FOBT that would be given to them within the following month was measured on a seven-point Likert scale The intentions reported by participants who received absolute risk (mean 3.65, n=40) or absolute plus either low (mean 6.43, n=38) or high (mean 6.65, n=39) comparative risk information were statistically significantly higher ($p<0.05$) than those participants in the control group who were provided with the same written information but without risk estimates (mean 2.21, n=43). The mean intention reported by the group which received the comparative risk was also significantly higher than for the absolute risk only group. In Seitz *et al.* women were separated into those with an estimated 10-year breast cancer risk above or below 1.5%. Intention to wait until age 50 before undergoing a mammogram was measured for those with a risk <1.5% and intention to start or continue to undergo mammograms in their 40s for those with a risk ≥1.5. In the low risk group, all risk-based intervention conditions resulted in a significant increase in the percentage of women planning to wait to age 50. However, in the high risk group no such significant difference was seen.

The eighth study by Lipkus *et al.*[37] reported the difference in intentions to get a mammogram between one group that received a one-page handout including their estimated absolute risk and another group that received the same handout plus information concerning how their risk compared to a woman of their age and race at the lowest level of risk. Immediately after the provision of risk information, overall 2.5%, 67.8%, and 24.8% reported that the risk information lowered, did not affect, or increased their intentions to undergo a mammogram respectively, with no differences between the groups.

Attendance at screening

For peer review only - http://bmjopen.bmj.com/site/about/guidelines.xhtml

Twelve RCTs reported attendance at screening: six for mammography[31,34,38–41]; five for colorectal cancer[30,32,33,38,42]; and one for cervical cancer[21]. Except for one high quality RCT in which the intervention group received information sheets including general information on breast cancer risk alongside personalised risk information and telephone counselling and the offer for more intensive group or genetic counselling[41], all showed no effect of the risk-based interventions as shown in the meta-analysis (Figure 2) with a combined RR of 1.02 (95%CI: 0.98-1.03, I^2: 61.6%).

Intention to change health-related behaviours

Intention to tan or protect skin

One RCT by Greene and Brinn measured intention to tan on a six-item Likert-type scale and intention to protect skin using a three-item scale[43]. Participants who completed a self-assessment risk score alongside receiving generic information about tanning, tanning beds and sun exposure reported significantly decreased intentions to use tanning beds than those receiving the same generic information alone (2.68, n=70 compared to 3.19, n=71, $p<0.05$). In contrast there were no significant differences in intentions to protect skin (2.38, n=70 compared to 2.49, n=71, $p>0.05$).

Change in health-related behaviours

Smoking status

One high quality RCT[23] reported the impact of risk information on smoking status. Receiving a personalised risk estimate in addition to a generic leaflet did not predict self-report smoking status at six months in current smokers ($p=0.66$) but was associated with an increased odds of remaining a former smoker in those who had recently quit (OR 1.91 (95%CI 1.03-3.55)).

For peer review only - http://bmjopen.bmj.com/site/about/guidelines.xhtml

Sun exposure and sun protection habits

Two RCTs[22,44] measured sun protection habits by survey completion at baseline and follow up. One by Glanz *et al.* compared the effect on childhood sun exposure and sun protection habits of three mailings with personalised risk feedback, interactive skin cancer education materials and a family fun guide to a single mailing of standardised skin cancer information[44]. The other by Glazebrooke *et al.* compared usual care with a self-directed computer program including individualised feedback of risk alongside sections on skin protection, how to detect melanoma, dangers of sun exposure, how to check skin and how to reduce risk[22]. Both showed increases in overall sun protection habits (increase in sun protection habits index 0.19 in the intervention group compared to 0.14, p=0.02[44] and mean difference in skin protective behaviour score between intervention and control at six month follow-up 0.33 (95% CI 0.09, 0.57) [22]) with variable results for individual aspects including wearing a sun hat, wearing a shirt, wearing sunglasses, use of sun cream, number of sunburns, staying in the shade, and sun exposure during weekdays and weekends.

Tanning bed usage

The RCT by Greene and Brinn[43] measured change in tanning behaviour and tanning bed usage. Participants who completed the self-assessment risk score reported lower rates of tanning bed usage in the previous month at follow-up (2.18, n=70 compared to 3.76, n=71, $p<0.05$) but no difference in change in tanning behaviour from pre- to post-intervention (-1.25, n=70 compared to -2.08, n=71, $p>0.05$).

Self/parent skin examination

The two RCTs by Glanz et al. and Glazebrooke et al., measured rates of skin examination in adults[22] or parents and children[44]. Both showed statistically significant increases among adults

For peer review only - http://bmjopen.bmj.com/site/about/guidelines.xhtml

and parents receiving personalised risk information ($p<0.05$) while the increase in parents examining their children was not statistically significant ($p=0.06$).

Clinical breast examination and breast self-examination

Three RCTs[31,40,41] measured rates of clinical breast examination and/or breast self-examination following provision of risk information. In the RCT by Bodurtha *et al.*, no significant differences were seen between those randomised to receive printed sheets including estimates of 5-year and lifetime risk of breast cancer alongside information addressing barriers to mammography, breast cancer seriousness and benefits of yearly mammography and those receiving general information about breast cancer prevention practices not tailored to their risk level for either frequency of clinical breast examination (crude rates: 91.4% vs 91.0%; adjusted OR: 1.00 (95%CI: 0.60 to 1.66)) or breast self-examination (crude rates: 56.8% vs 57.6%; adjusted OR: 0.95 (95%CI: 0.67 to 1.33)[31]. The other two studies, both by Bowen *et al.*, found significantly ($p<0.01$) greater increases in the proportion reporting performing breast self-examination in the intervention groups (35% to 52% and 36% to 62%) compared with controls (33% to 36% and 38% to 40%)[40,41]. However, both these studies compared intensive interventions (four weekly 2-hour sessions led by a health counsellor[40] or information sheets plus telephone counselling and the offer of more intensive group or genetic counselling[41]) with delayed intervention.

DISCUSSION

This systematic review is, to our knowledge, the first review of the impact of interventions in all settings incorporating information about cancer risk on intention and behaviour in the general population. The findings show that such interventions do not affect intention to attend or attendance at screening. There is limited evidence that they increase intention to tan,

For peer review only - http://bmjopen.bmj.com/site/about/guidelines.xhtml

smoking abstinence, sun protection, adult skin self-examination and breast examination but this was not seen for intention to protect skin, smoking cessation or parental child skin examination. There is a notable absence of studies assessing the impact on diet, physical activity and alcohol consumption with only one reporting smoking status and none including objective measures of behaviour.

Our finding that interventions incorporating information about cancer risk had no effect on intention to attend or attendance at screening is consistent with a previous Cochrane review in which personalised risk communication had little effect on the uptake of screening tests (fixed-effect OR 0.95 (95% CI 0.78 to 1.15))[16]. However, as in that review, there was evidence of increased concordance between screening preferences and recommendations and decreased ambivalence. This supports the suggestion made in that review that personalised risk information might be useful for shared and informed decision making. For example, in surveys of participants about their knowledge and values for cancer screening decisions and decision-making processes, only 21% report feeling extremely well informed[45] and the majority overestimate lifetime risk of cancer incidence and mortality[45,46]. While providing individuals with information about their cancer risk may therefore not influence overall rates of screening it may contribute to the decision to take up screening or not at an individual level and support shared decision making.

The absence of significant effects on health-related behaviours is also consistent with research in other disease areas, such as cardiovascular disease, where systematic reviews have found only few studies reporting behaviour change and no significant effects on lifestyle[47–49]. This is perhaps not surprising given that behaviour change is influenced by many other factors, including health beliefs, social context, the environment, and personal attributes such as time

For peer review only - http://bmjopen.bmj.com/site/about/guidelines.xhtml

orientation[12,13].However, there was no evidence that interventions that include information about cancer risk result in harm through false reassurance and the adoption of unhealthy behaviours. This is important as on average many of the general population overestimate their own risk of cancer[30,35,40,50–52] and so if information about cancer risk were routinely provided within clinical practice large numbers would be receiving an estimate lower than their prior perceptions.

The main strengths of this review are the systematic search of multiple electronic databases and the broad inclusion criteria. This allowed us to include studies that assess the impact of interventions incorporating cancer risk information on multiple behavioural outcomes. However, from nearly 40,000 titles and abstracts, we only included 14 with an additional 5 found through citation searching. This highlights the challenge in identifying studies in this area in which the primary purpose may not be related to the provision of risk information. There was also significant heterogeneity in the outcome measures included, duration of follow-up and method of recruitment across the included studies. For all outcomes except attendance at screening there were either too few studies to meaningfully pool results or each study used different non-comparable measures. The duration of follow-up varied from 1 to 18 months. Although this makes pooling the findings more difficult, the studies with shorter follow-up were those with intention as the outcome measures and, of the 10 studies reporting health-related behaviours, five had a follow-up period of a year or more and three a period of six months. It is therefore unlikely that the studies as a whole were too short to detect changes in behaviour or reflected only immediate un-sustained changes.

A further limitation is that many of the interventions consisted of provision of risk information alongside a range of additional information, either written or delivered in person or in groups.

For peer review only - http://bmjopen.bmj.com/site/about/guidelines.xhtml

Separating the effect of the risk information from those additional elements of the interventions was therefore not possible. However, we chose not to exclude these studies from this review because it is unlikely that risk information would be incorporated into routine practice in isolation and, if anything, including them would overestimate the effect of the risk information. It is also possible that the findings do not reflect the potential impact of interventions incorporating information about cancer risk on the general population as a whole: half of the included studies focused on female cancers and so only recruited women and all were subject to recruitment bias with the participants who agreed to take part potentially more interested in their cancer risk or more healthy, resulting in a bias in either direction.

In addition to these specific limitations of our review, the findings also suggest a number of areas for future research. In particular, the absence of studies assessing the impact on diet, physical activity and alcohol consumption, and only one study reporting smoking cessation, demonstrate the need for trials assessing change in these behaviours, preferably measured objectively , including measures of other theory based determinants of behaviour change (for example, self-efficacy). Only with such data will we be able to assess whether such individualised approaches have a place alongside population-wide prevention strategies.

ACKNOWLEDGEMENTS

The authors thank Isla Kuhn, Reader Services Librarian, University of Cambridge Medical Library, for her help developing the search strategy.

Contributors

JUS developed the protocol, completed the search, screened articles for inclusion, extracted data, synthesized the findings, interpreted the results and drafted the manuscript. BS developed

For peer review only - http://bmjopen.bmj.com/site/about/guidelines.xhtml

the protocol, screened articles for inclusion, extracted data, interpreted the results and critically revised the manuscript. SS synthesized the findings and critically revised the manuscript. KM extracted data, interpreted the results and critically revised the manuscript. SJG developed the protocol, screened articles for inclusion, interpreted the results and critically revised the manuscript. All authors approved the final version.

Funding

JUS and KM are funded by a Cancer Research UK/BUPA Foundation Cancer Prevention Fellowship (C55650/A21464). BS was supported by the Medical Research Council [MC_UU_12015/4]. SJS is supported by the Medical Research Council www.mrc.ac.uk [Unit Programme number MC_UU_12015/1]. The University of Cambridge has received salary support in respect of SJG from the NHS in the East of England through the Clinical Academic Reserve. The views expressed are those of the authors and not necessarily those of the NHS or Department of Health.

All researchers were independent of the funding body and the funder had no role in data collection, analysis and interpretation of data; in the writing of the report; or decision to submit the article for publication.

For peer review only

Data sharing

All data are available from the reports or authors of the primary research. No additional data is available.

Competing Interests

All authors have completed the Unified Competing Interest form at *www.icmje.org/coi_disclosure.pdf* (available on request from the corresponding author) and

declare that (1) they have no support from or relationships with companies that might have an interest in the submitted work in the previous 3 years; (2) their spouses, partners, or children have no financial relationships that may be relevant to the submitted work; and (3) they have no non-financial interests that may be relevant to the submitted work.

The corresponding author has the right to grant on behalf of all authors and does grant on behalf of all authors, an exclusive licence on a worldwide basis to the BMJ Publishing Group Ltd and its Licensees to permit this article (if accepted) to be published in BMJ editions and any other BMJPGL products and sub-licences to exploit all subsidiary rights, as set out in our licence (http://resources.bmj.com/bmj/authors/checklists-forms/licence-for-publication).

All authors had full access to all of the data in the study and can take responsibility for the integrity of the data and the accuracy of the data analysis

The corresponding author affirms that the manuscript is an honest, accurate, and transparent account of the study being reported; that no important aspects of the study have been omitted; and that any discrepancies from the study as planned (and, if relevant, registered) have been explained.

FIGURE LEGENDS

Figure 1. PRISMA flow diagram

Figure 2. Relative risk for adherence to recommended screening post intervention. CRC – colorectal cancer; FOBT – faecal occult blood test; AR – absolute risk; CR – comparative risk

REFERENCES

1 National Cancer Institute. The nation's investment in cancer research. A plan and budget proposal for the fiscal year 2006. 2006.

2 McCaul KD, O'Donnell SM. Naive beliefs about breast cancer risk. *Women's Heal*

For peer review only - http://bmjopen.bmj.com/site/about/guidelines.xhtml

1998;**4**:93–101.

3 Emery J, Morris H, Goodchild R, *et al.* The GRAIDS Trial: a cluster randomised controlled trial of computer decision support for the management of familial cancer risk in primary care. *Br J Cancer* 2007;**97**:486–93.

4 Weinstein ND, Atwood K, Puleo E, *et al.* Colon Cancer: Risk Perceptions and Risk Communication. *J Health Commun* 2004;**9**:53–65.

5 Waters EA, Klein WM, Moser RP, *et al.* Correlates of unrealistic risk beliefs in a nationally representative sample. *J Behav Med* 2011;**34**:225–35.

6 Parkin DM, Boyd L, Walker LC. The fraction of cancer attributable to lifestyle and environmental factors in the UK in 2010. *Br J Cancer* 2011;**105 Suppl**:S77-81.

7 Cancer Research UK. Perceptions of Risk Survey 2008 : Key Findings. 2008.

8 Grunfeld EA, Ramirez AJ, Hunter MS, *et al.* Women's knowledge and beliefs regarding breast cancer. *Br J Cancer* 2002;**86**:1373–8.

9 Redeker C, Wardle J, Wilder D, *et al.* The launch of Cancer Research UK's 'Reduce the Risk' campaign: baseline measurements of public awareness of cancer risk factors in 2004. *Eur J Cancer* 2009;**45**:827–36.

10 Wardle J, Waller J, Brunswick N, *et al.* Awareness of risk factors for cancer among British adults. *Public Health* 2001;**115**:173–4.

11 Ryan AM, Cushen S, Schellekens H, *et al.* Poor Awareness of Risk Factors for Cancer in Irish Adults: Results of a Large Survey and Review of the Literature. *Oncologist* 2015;**20**:372–8.

12 Rogers R. A protection motivation theory of fear appeals and attitude change. *J Psychol* 1975;**91**:93–114.

13 Witte K. Putting the fear back into fear appeals: The extended parallel process model. *Commun Monogr* 1992;**59**:329–49.

For peer review only - http://bmjopen.bmj.com/site/about/guidelines.xhtml

14 Walker JG, Licqurish S, Pirotta M, *et al.* Cancer Risk Assessment Tools in Primary Care: A Systematic Review of Randomized Controlled Trials. 2015;:480–9.

15 Albada A, Ausems MGEM, Bensing JM, *et al.* Tailored information about cancer risk and screening: a systematic review. *Patient Educ Couns* 2009;**77**:155–71.

16 Edwards AGK, Evans R, Dundon J, *et al.* Personal risk communication for informed decision making about taking screening tests. *Cochrane Database Syst Rev 2006* Published Online First: 2006. doi:10.1002/14651858.CD001865.pub2

17 Moher D, Liberati A, Tetzlaff J, *et al.* Preferred reporting items for systematic reviews and meta-analyses: the PRISMA statement. *Int J Surg* 2010;**8**:336–41.

18 Critical Appraisal Skills Programme (CASP) Checklists. http://www.casp-uk.net/casp-tools-checklists

19 Hedges L V., Vevea JL. Fixed- and random-effects models in meta-analysis. *Psychol Methods* 1998;**3**:486–504.

20 Knol MJ, Le Cessie S, Algra A, *et al.* Overestimation of risk ratios by odds ratios in trials and cohort studies: Alternatives to logistic regression. *CMAJ* 2012;**184**:895–9.

21 Holloway RM, Wilkinson C, Peters TJ, *et al.* Cluster-randomised trial of risk communication to enhance informed uptake of cervical screening. *Br J Gen Pract* 2003;**53**:620–5.

22 Glazebrook C, Garrud P, Avery A, *et al.* Impact of a multimedia intervention 'Skinsafe' on patients' knowledge and protective behaviors. *Prev Med (Baltim)* 2006;**42**:449–54.

23 Sherratt FC, Marcus MW, Robinson J, *et al.* Utilizing Lung Cancer Risk Prediction Models to Promote Smoking Cessation: Two Randomized Controlled Trials. *Am J Health Promot* Published Online First: 2016. doi:10.1177/0890117116673820

24 Gail MH, Brinton LA, Byar DP, *et al.* Projecting individualized probabilities of developing breast cancer for white females who are being examined annually. *J Natl*

For peer review only - http://bmjopen.bmj.com/site/about/guidelines.xhtml

Cancer Inst 1989;**81**:1879–86.

25 Colditz G a, Atwood K a, Emmons K, *et al.* Harvard report on cancer prevention volume 4: Harvard Cancer Risk Index. Risk Index Working Group, Harvard Center for Cancer Prevention. *Cancer Causes Control* 2000;**11**:477–88.

26 Cassidy A, Duffy SW, Myles JP, *et al.* Lung cancer risk prediction: a tool for early detection. *Int J cancer* 2007;**120**:1–6.

27 Yoon PW, Scheuner MT, Jorgensen C, *et al.* Developing Family Healthware, a family history screening tool to prevent common chronic diseases. *Prev Chronic Dis* 2009;**6**:A33.

28 Wilkinson CE, Peters TJ, Stott NC, *et al.* Prospective evaluation of a risk scoring system for cervical neoplasia in primary care. *Br J Gen Pract* 1994;**44**:341–4.

29 Glanz K, Schoenfeld E, Weinstock MA, *et al.* Development and reliability of a brief skin cancer risk assessment tool. *Cancer Detect Prev* 2003;**27**:311–5.

30 Lipkus IM, Klein WMP. Effects of communicating social comparison information on risk perceptions for colorectal cancer. *J Health Commun* 2006;**11**:391–407.

31 Bodurtha J, Quillin JMJ, Tracy K a, *et al.* Mammography screening after risk-tailored messages: the women improving screening through education and risk assessment (WISER) randomized, controlled trial. *J women's Heal* 2009;**18**:41–7.

32 Schroy PC, Emmons KM, Peters E, *et al.* Aid-assisted decision making and colorectal cancer screening: A randomized controlled trial. *Am J Prev Med* 2012;**43**:573–83.

33 Trevena L, Irwig L, Barratt A, *et al.* Randomized trial of a self-administered decision aid for colorectal cancer screening. *J Med Screen* 2008;**15**:76–82.

34 Davis S, Stewart S, Bloom J. Increasing the accuracy of perceived breast cancer risk: results from a randomized trial with Cancer Information Service callers. *Prev Med* 2004;**39**:64–73.

For peer review only - http://bmjopen.bmj.com/site/about/guidelines.xhtml

35 Helmes AW, Culver JO, Bowen DJ. Results of a randomized study of telephone versus in-person breast cancer risk counseling. *Patient Educ Couns* 2006;**64**:96–103.

36 Seitz HH, Gibson L, Skubisz C, *et al.* Effects of a risk-based online mammography intervention on accuracy of perceived risk and mammography intentions. *Patient Educ Couns* 2016;**99**:1647–56.

37 Lipkus IM, Biradavolu M, Fenn K, *et al.* Informing women about their breast cancer risks: truth and consequences. *Health Commun* 2001;**13**:205–26.

38 Rubinstein WS, Acheson LS, Neill SMO, *et al.* Clinical utility of family history for cancer screening and referral in primary care: A report from the Family Healthware Impact Trial. 2012;**13**:956–65.

39 Rimer BK, Halabi S, Sugg Skinner C, *et al.* Effects of a mammography decision-making intervention at 12 and 24 months. *Am J Prev Med* 2002;**22**:247–57.

40 Bowen DJ, Powers D, Greenlee H. Effects of Breast Cancer Risk Counseling for Sexual Minority Women. *Health Care Women Int* 2006;**27**:59–74.

41 Bowen DJ, Powers D. Effects of a mail and telephone intervention on breast health behaviors. *Health Educ Behav* 2010;**37**:479–89.

42 Sequist TD, Zaslavsky AM, Colditz GA, *et al.* Electronic patient messages to promote colorectal cancer screening: a randomized controlled trial. *Arch Intern Med* 2011;**171**:636–41.

43 Greene K, Brinn LS. Messages Influencing College Women's Tanning Bed Use: Statistical versus Narrative Evidence Format and a Self-Assessment to Increase Perceived Susceptibility. *J Health Commun* 2003;**8**:443.

44 Glanz K, Steffen AD, Schoenfeld E, *et al.* Randomized Trial of Tailored Skin Cancer Prevention for Children: The Project SCAPE Family Study. *J Health Commun* 2013;**18**:1368–83.

For peer review only - http://bmjopen.bmj.com/site/about/guidelines.xhtml

45 Hoffman RM, Elmore JG, Pignone MP, *et al.* Knowledge and values for cancer screening decisions: Results from a national survey. *Patient Educ Couns* 2016;**99**:624–30.

46 Hoffman RM, Lewis CL, Pignone MP, *et al.* Decision-Making Processes for Breast, Colorectal, and Prostate Cancer Screening: The DECISIONS Survey. *Med Decis Mak* 2010;**30**:53–64.

47 Usher-Smith JA, Silarova B, Schuit E, *et al.* Impact of provision of cardiovascular disease risk estimates to healthcare professionals and patients: a systematic review. *BMJ Open* 2015;**5**:e008717.

48 Willis A, Davies M, Yates T, *et al.* Primary prevention of cardiovascular disease using validated risk scores: a systematic review. *J R Soc Med* 2012;**105**:348–56.

49 Sheridan SL, Viera AJ, Krantz MJ, *et al.* The effect of giving global coronary risk information to adults: a systematic review. *Arch Intern Med* 2010;**170**:230–9.

50 Quillin JM, Fries E, McClish D, *et al.* Gail model risk assessment and risk perceptions. *J Behav Med* 2004;**27**:205–14.

51 McCaul K, Canevello A, Mathwig J, *et al.* Risk communication and worry about breast cancer. *Psychol Health Med* 2003;**8**:379–89.

52 Lipkus IM, Klein WM, Rimer BK. Communicating breast cancer risks to women using different formats. *Cancer Epidemiol biomarkers Prev* 2001;**10**:895–8.

For peer review only

Table 1. Details of the setting and key outcomes of the included studies

Author, year	Cancer site(s)	Follow-up	Setting and participants	Risk level / co-morbidities	Outcome(s)	Quality*
Bodurtha 2009	Breast	18 months	899 women with no history of breast cancer recruited from waiting rooms of four women's health clinics	Not given	Mammography, clinical breast examinations, breast self-examination, mammography intentions	M-H
Bowen 2006	Breast	6 and 24 months	150 sexual minority women recruited via public advertisements	Mean Gail lifetime risk 12%	Breast self-examination, breast cancer screening	H
Bowen 2010	Breast	12 months	1,366 women recruited via purchased lists of telephone numbers with no previous diagnosis of breast cancer	Mean Gail lifetime risk 12%	Breast self-examination, mammography	
Davis, 2004	Breast	1 month	392 women with no history cancer calling the Cancer Information Service	27% 2-6% lifetime risk; 32% 6-9% lifetime risk; 41% 9-46% lifetime risk	Adherence to breast cancer screening, intention for breast cancer screening	M
Glanz 2013	Skin	16 weeks	Convenience sample of 1047 parents not currently being treated for skin cancer recruited through schools and community centres	38% high risk	Sun protection habits, sun exposure, skin examination by parents	M
Glazebrook 2006	Skin	6 months	589 recruited from 10 primary care practices from a convenience sample of appointments	Not given	Sun protection habits	M
Greene 2003	Skin	3-4 weeks	141 undergraduates at one university who received extra credit for participation	Not given	Intention to tan, actual tan bed usage	L-M
Helmes, 2006	Breast	3 months	Random sample of 340 members of state healthcare system with no history of breast/ovarian cancer or testing for cancer risk	Mean 9.5% (3.2) lifetime risk	Intention to have mammogram and clinical breast examination, intention to do breast self-examination	M
Holloway, 2003	Cervical	0, 4 years	1890 women attending routine cervical smear test at one of 29 GP practices	78-80% very low risk; 20-22% low risk	Preference for future screening interval, actual screening behaviour	M-H
Lipkus 2006	Colorectal	0	160 members of general public with no history of CRC or screening for CRC recruited through newspaper advertisements	Not given	Ambivalence, intention to screen using a FOBT, actual FOBT screening rates	M
Lipkus, 2001	Breast	0	121 members of general public recruited through newspaper advertisements	Mean 10 year risk 2.65% (SD 1.13)	Mammography screening and intentions	M
Rimer 2002	Breast	1 and 2 years	752 women aged 40-44 and 50-54 enrolled in a personal care plan	Mean 10 year risk 2.7%	Mammography	M
Rubenstein 2011	Breast, ovarian, colon	6 months	3786 patients from primary care clinic records with no history of colon, breast or ovaraian cancer invited by mail following record review	34% moderate or strong risk of ≥ 1 of the cancers	CRC screening, mammography	M

For peer review only

Schroy, 2011	Colorectal	0	666 patients due for bowel screening identified from monthly audits of one hospital's electronic medical record	Average	Preferences, satisfaction with the decision-making process, screening intentions, and test concordance	M-H
Schroy, 2012	Colorectal	0, 1, 3, 6 and 12 months	825 patients due for bowel screening identified from monthly audits of one hospital's electronic medical record	Average	Completion of a CRC screening test	H
Seitz 2016	Breast	0	2,918 women aged 35-49 with no history of breast cancer or a genetic mutation in BRCA1 or BRCA2 recruited through a survey company	42% 10 year risk <1.5% (mean 1.08 SD 0.01); 58% 10 year risk ≥1.5% (mean 2.53 SD 0.04)	Mammography intentions	M
Sequist 2012	Colorectal	1 and 4 months	1,103 patients from 14 ambulatory health centres who were overdue for colorectal cancer screening	Average	CRC screening	M
Sherratt 2016	Lung	6 months	297 current and 216 recent former smokers aged 18-60 without a history of lung cancer and attending smoking cessation services	Not given	Smoking status	H
Trevena 2008	Colorectal	1 month	314 patients recruited from 6 primary care practices without a history of colorectal cancer	Not given	Screening intentions, CRC screening	M

RCT – randomised controlled trial; CRC – colorectal cancer; CT computerised tomography; FOBT – faecal occult blood test
* L – low, M – medium, H - high

For peer review only

For peer review only - http://bmjopen.bmj.com/site/about/guidelines.xhtml

Table 2. Details of the risk-based interventions in each of the included studies

Author, year	Risk tool	Intervention group(s)	Comparison (where applicable)	Format of risk
Bodurtha 2009	Gail model (5 year and lifetime)	Information sheets with risk level and handouts addressing traditional constructs of Health Belief Model including barriers to mammography, breast cancer seriousness, individual risk for breast cancer, and benefits of yearly mammography	General information about breast cancer prevention practices, including mammography	Usual (<15%), Moderate (15-30%) or Strong (>30%)
Bowen 2006	Gail model (5 year, 10 year and at age 79)	Four weekly 2-hour sessions led by a health counsellor focusing on risk assessment and education, screening, stress management and social support	Delayed intervention	No details given
Bowen 2010	Gail model (lifetime)	Information sheets with general information on breast cancer risk and personalised risk information plus telephone counselling and offer for more intensive group or genetic counselling	Delayed intervention	Bar graph of absolute lifetime risk along with age-appropriate estimates for the "average risk" woman
Davis, 2004	BRCA tool (updated version of Gail model) (lifetime)	10min brief intervention designed to increase accuracy of perceived risk including results of risk assessment and screening recommendations tailored to participant's stage of adoption of mammography and follow up written information	No intervention	Verbal over the telephone. No additional details given.
Glanz 2013	Children's BRAT	Three mailings with personalised risk feedback, interactive skin cancer education materials, a family fun guide and suggestions for overcoming barriers and reminders to engage in preventive practices	Single mailing of standardised skin cancer information	No details given
Glazebrooke 2006	No details given	Self-directed computer program including sections on skin protection, how to detect melanoma, dangers of sun exposure, how to check skin, how to reduce risk and individualized feedback of risk	Usual care	Comparative risk
Greene 2003	Relative risk adapted from "ADD Wants to Convert"	Self-assessment of risk alongside generic messages about tanning, tanning beds and sun exposure	Generic messages about tanning, tanning beds and sun exposure	Numerical scale from 1-36
Helmes, 2006	Gail model (lifetime)	Face-to-face or telephone intervention consisting of 8 items: 1) a personal risk sheet ; 2) a personal computer-drawn pedigree; 3) a 23 page participant booklet; 4) Breast self-examination brochure; 5) Pap smear and mammography	No intervention	Bar charts of absolute % risk with numerical % alongside for the individual, an average-risk woman, and a high-risk woman

For peer review only - http://bmjopen.bmj.com/site/about/guidelines.xhtml

		brochure; 6) BSE shower card; 7) pictures of chromosomes and gene mutations; 8) a list of community resources for breast cancer		
Holloway, 2003	Wilkinson score	Brief 10 minute counselling session integrated with smear test appointment including relative and absolute risks and then negotiation of appropriate screening intervals	Usual care	Comparative and absolute risk in pictures and numbers
Lipkus 2006	Not given	Written information about CRC, CRC screening methods and CRC risk factors plus either 1) tailored CRC risk factor information or 2) tailored CRC risk factor information plus information on whether their total number of CRC risk factors was greater or not than average	Written information about CRC, CRC screening methods, and CRC risk factors	Narrative comparative risk
Lipkus, 2001	Gail model (10 year)	1 page handout describing the Gail model plus absolute risk alone	As for intervention group plus how their risk compared to a woman of their age and race at the lowest level of risk	Absolute risk +/- risk of a woman at the lowest level of risk as percentages in a pie chart
Rimer 2002	Gail model (10 year and lifetime)	Tailored print booklet and brief tailored newspaper plus personalized risk	Usual care (postcard reminder)	Absolute risk as a percentage
Rubenstein 2011	Family Healthware tool	Written personalized risk assessment and tailored prevention messages	Written generalized prevention messages	Qualitative risk - weak, moderate or strong familial risk
Schroy, 2011	Harvard cancer risk model (10 year)	Interactive 20-30 min computer-based decision aid plus personalized risk assessment	Interactive 20-30 min computer-based decision aid alone	Thermograph, indicating where the participant is along with a description e.g. your risk is below average
Schroy, 2012	Harvard cancer risk model (10 year)	Interactive 20-30 min computer-based decision aid plus personalized risk assessment followed immediately by a meeting with their providers to discuss screening and identify a preferred screening strategy. Providers received written notification hand-delivered by all the patients acknowledging that they were participating in the "CRC decision aid study" at the time of the visit to ensure that screening was discussed	As for intervention but without personalized risk assessment	Qualitative framing ("very much below average risk" to "very much above average risk") with accompanying suggestions for behaviour modifications that might reduce risk, including a strong recommendation for screening, regardless of risk
Seitz 2016	Gail model (10 year)	Online risk plus basic information about mammography and national recommendations plus either 1) statements about women making choices 2) untailored exemplars of women making choices or 3) exemplars of similar women making choices	No information or the same basic information as intervention group	Absolute risk and risk of an average-risk age-matched women as numeric frequencies and icon arrays

For peer review only - http://bmjopen.bmj.com/site/about/guidelines.xhtml

Sequist 2011	Harvard cancer risk model (10 year)	Personalized electronic message highlighting their overdue screening status and providing a link to a web-based tool to assess their risk	No contact	Comparative risk on 7-point ordinal scale from very-much below average to very-much above average and in interactive graphical format
Sherratt 2016	Liverpool Lung Project model (5 year at age 70)	Personalised risk plus booklet stating the association between smoking and lung cancer and highlighting that quitting smoking was the best thing to do	As for intervention but without personalized risk assessment	Verbal and written absolute risk if continue to smoke and if stop smoking alongside icon arrays
Trevena 2008	No details given	20 page booklet including personalized risk, absolute reduction in colorectal cancer mortality with screening over the next 10 years, probability of test outcomes from screening and information about how to get screeed.	3 page booklet with information and recommendations about screening	Words and 1000-face diagrams

CRC – colorectal cancer

For peer review only

For peer review only - http://bmjopen.bmj.com/site/about/guidelines.xhtml

Table 3. Summary of evidence on outcomes

Outcome measure	Number of studies	Studies with significant positive effect	Studies with no effect	Best evidence synthesis
Screening				
Preferences for screening	2	1 medium/high quality and 1 high quality RCT	None	Evidence of positive effect
Intention to attend screening	8	1 medium quality RCT*	1 high quality, 1 medium/high quality and 4 medium quality RCTs*	Evidence of no effect
Attendance at screening	12	1 high quality RCT	2 high quality, 2 medium/high quality and 7 medium quality studies	Evidence of no effect
Health-related behaviours				
Intention to change health-related behaviours				
To tan	1	1 low/medium RCT	None	Limited evidence of positive effect
To protect skin	1	None	1 low/medium RCT	Limited evidence of no effect
Health-related behaviours				
Smoking cessation	1	None	1 high quality RCT	Limited evidence of no effect
Smoking abstinence	1	1 high quality RCT	None	Limited evidence of positive effect
Sun protection	2	2 medium quality RCTs		Indicative evidence of positive effect
Tanning bed usage	1	None	1 low/medium RCT	Limited evidence
Adult skin examination	2	2 medium quality RCTs	None	Indicative evidence of positive effect
Child skin examination	1	None	1 medium quality RCT	Limited evidence of no effect
Breast examination	3	2 high quality RCTs	1 medium/high RCT	Indicative evidence of positive effect
Diet	0	None	None	No evidence
Physical activity	0	None	None	No evidence
Alcohol	0	None	None	No evidence

* 1 medium quality study reported a significant positive effect in low risk women and no effect in high risk women

For peer review only - http://bmjopen.bmj.com/site/about/guidelines.xhtml

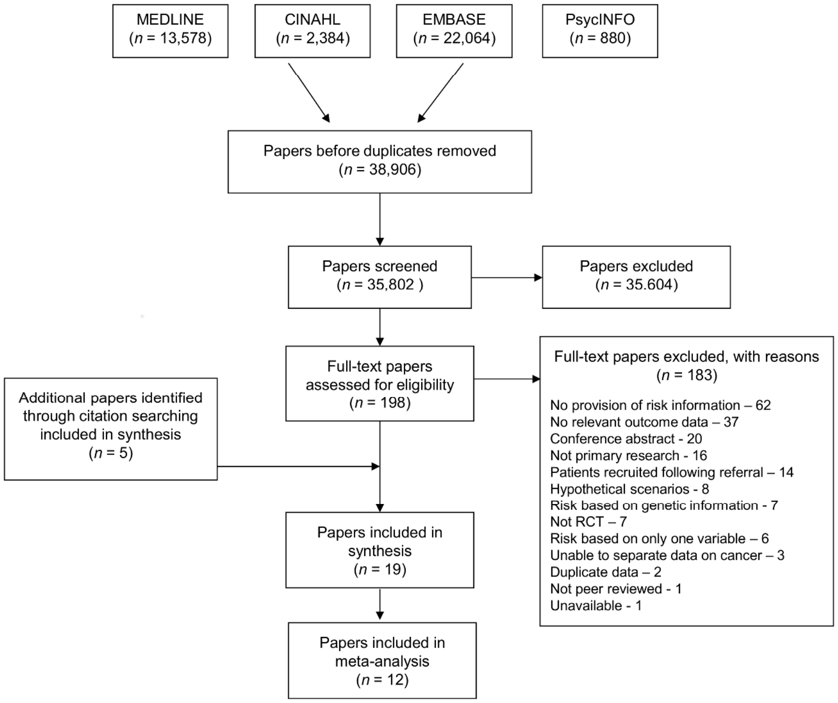

Figure 1. PRISMA flow diagram

254x190mm (300 x 300 DPI)

ew only

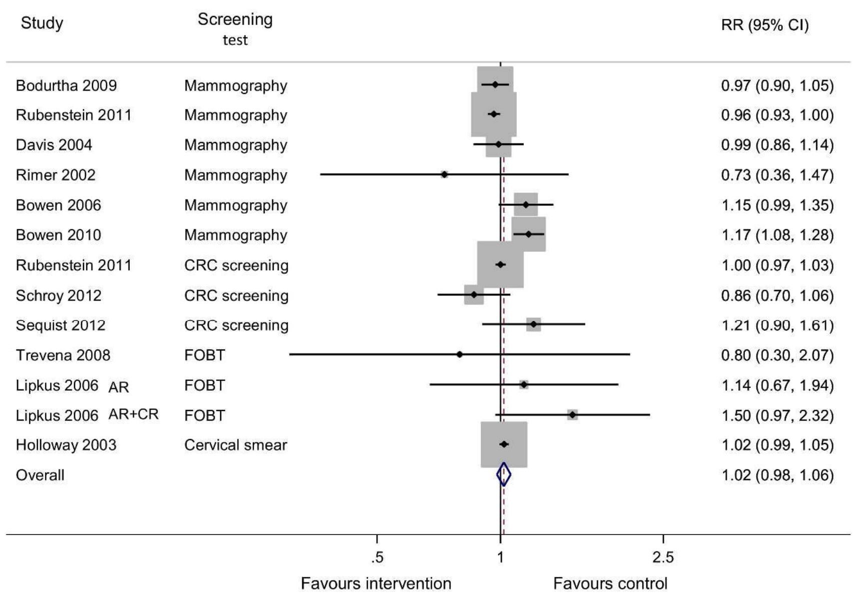

Figure 2. Relative risk for adherence to recommended screening post intervention. CRC – colorectal cancer; FOBT – faecal occult blood test; AR – absolute risk; CR – comparative risk

254x190mm (300 x 300 DPI)

w only

Supplementary file 1 – Complete search strategy

Medline and Cinahl

S28	S26 NOT S27
S27	review
S26	S24 AND S25
S25	S13 NOT S15
S24	S14 OR S16 OR S17 OR S21 OR S22 OR S23
S23	(behaviour OR behavior) AND health
S22	(MH "Health Behavior+") OR (MH "Risk Reduction Behavior+")
S21	S18 OR S20
S20	S19 AND S1
S19	screen* AND uptake OR attendance OR intention OR adherence
S18	(MM "Early Detection of Cancer/UT")
S17	anxiety* OR worry* OR denial* OR hopelessness* OR avoidance*
S16	efficacy OR effectiv*
S15	PT review OR PT letter OR PT comment OR PT editorial
S14	percep* OR perceive* OR understand* OR understood* OR accura* OR comprehen*
S13	S9 NOT S12
S12	S10 OR S11
S11	(MH "Prognosis+")
S10	prognos* OR treatment* OR surgery*
S9	S1 AND S8
S8	S6 OR S7
S7	(MH "Risk Assessment+")
S6	S4 AND S5
S5	score* OR model* OR predict* OR tool*
S4	S2 OR S3
S3	(MH "Risk+")
S2	risk*
S1	"cancer" OR (MH "Neoplasms+")

Embase

1	cancer.mp. or exp neoplasm/
2	exp risk/ or risk*.mp.
3	(score* or model* or predict* or tool*).mp. [mp=title, abstract, heading word, drug trade name, original title, device manufacturer, drug manufacturer, device trade name, keyword]
4	2 and 3
5	exp risk assessment/
6	4 or 5
7	1 and 6
8	(percep* or perceive* or understand* or understood* or accura* or comprehen*).mp. [mp=title, abstract, heading word, drug trade name, original title, device manufacturer, drug manufacturer, device trade name, keyword]
9	(efficacy* or effectiv*).mp. [mp=title, abstract, heading word, drug trade name, original title, device manufacturer, drug manufacturer, device trade name, keyword]
10	exp prognosis/
11	(prognos* or treatment* or surgery*).mp. [mp=title, abstract, heading word, drug trade name, original title, device manufacturer, drug manufacturer, device trade name, keyword]

For peer review only

12 (review or letter or comment or editorial).pt.
13 (radiotherapy* or stage* or grade*).mp. [mp=title, abstract, heading word, drug trade name, original title, device manufacturer, drug manufacturer, device trade name, keyword]
14 (anxiety* or worry* or fatalism* or hopelessness* or denial* or avoid*).mp. [mp=title, abstract, heading word, drug trade name, original title, device manufacturer, drug manufacturer, device trade name, keyword]
15 8 or 9 or 14
16 10 or 11 or 12 or 13
17 exp cancer screening/
18 health behaviour.mp. or exp health behavior/
19 ((behaviour or behavior) and health).mp. [mp=title, abstract, heading word, drug trade name, original title, device manufacturer, drug manufacturer, device trade name, keyword]
20 (screen* and (uptake or attendance or intention or adherence)).mp. [mp=title, abstract, heading word, drug trade name, original title, device manufacturer, drug manufacturer, device trade name, keyword]
21 20 and 1
22 15 or 17 or 18 or 19 or 21
23 22 and 7
24 23 not 16
25 limit 24 to yr="2000 -Current"
26 25 not review.mp. [mp=title, abstract, heading word, drug trade name, original title, device manufacturer, drug manufacturer, device trade name, keyword]

PsycInfo

S20 S19 NOT review Limiters - Publication Year: 2000-2015
S19 S17 NOT (S10 OR S11 OR S12)
S18 S17 NOT (S10 OR S11 OR S12)
S17 S7 and (S8 or S9 or S13 or S15 or S16)
S16 health AND (behaviour OR behavior)
S15 S14 AND S1
S14 screen* AND (uptake OR attendance OR intention OR adherence)
S13 MM "Cancer Screening"
S12 (prognos* OR treatment* OR surgery*) AND (S10 OR S11)
S11 prognos* OR treatment* OR surgery*
S10 DE "Prognosis"
S9 efficacy or effectiv* or worry* or anxiety* or hopelessness* or denial*
S8 percep* OR perceive* OR understand* OR understood* OR accura* OR comprehen*
S7 (S1 AND S6)
S6 (S4 OR S5)
S5 DE "Risk Assessment"
S4 (S2 AND S3)
S3 score* OR model* OR predict* OR tool*
S2 risk*
S1 DE "Neoplasms" OR DE "Benign Neoplasms" OR DE "Breast Neoplasms" OR DE "Endocrine Neoplasms" OR DE "Leukemias" OR DE "Nervous System Neoplasms" OR DE "Terminal Cancer"

For peer review only

Supplementary file 2. Quality assessment of included studies

Author, date	Study addressed a clearly focused issue	Randomisation	Recruitment / comparability of study groups at baseline	Blinding	Exposure measurement	Outcome measurement	Comparability of study groups during study	Follow up	Overall
Bodurtha, 2009	⬤	⬤	●	•	⬤	⬤	●	●	M-H
Bowen 2006	⬤	⬤	⬤	•	⬤	⬤	⬤	●	H
Bowen 2010	⬤	⬤	⬤	•	⬤	⬤	⬤	●	H
Davis, 2004	⬤	⬤	●	•	⬤	⬤	●	•	M
Glanz, 2013	⬤	⬤	●	●	●	●	●	⬤	M
Glazebrook 2006	⬤	⬤	●	•	●	●	●	•	M
Greene, 2003	●	●	•	•	●	●	•	•	L-M
Helmes, 2006	⬤	●	●	•	●	●	●	●	M
Holloway, 2003	⬤	⬤	⬤	●	⬤	⬤	●	•	M-H
Lipkus , 2006	⬤	⬤	•	•	●	●	•	⬤	M
Lipkus, 2001	⬤	●	•	•	n/a	●	●	●	M
Rimer 2002	⬤	⬤	●	•	●	⬤	●	●	M
Rubenstein, 2011	⬤	⬤	⬤	•	⬤	●	⬤	⬤	M
Schroy, 2011	⬤	⬤	⬤	•	⬤	⬤	⬤	●	M-H
Schroy, 2012	⬤	⬤	⬤	•	⬤	⬤	⬤	⬤	H
Seitz 2016	●	●	●	•	●	●	●	•	M

For peer review only

Sequist 2011	●	●	●	•	●	●	●	n/a	M
Sherratt 2016	⬤	⬤	⬤	●	●	●	⬤	⬤	H
Trevena 2008	●	●	⬤	⬤	●	●	●	●	M

• Low (L) ● Medium (M) ⬤ High (H)

For peer review only

PRISMA 2009 Checklist

Section/topic	#	Checklist item	Reported on page #
**TITLE**			
Title	1	Identify the report as a systematic review, meta-analysis, or both.	1
**ABSTRACT**			
Structured summary	2	Provide a structured summary including, as applicable: background; objectives; data sources; study eligibility criteria, participants, and interventions; study appraisal and synthesis methods; results; limitations; conclusions and implications of key findings; systematic review registration number.	2
**INTRODUCTION**			
Rationale	3	Describe the rationale for the review in the context of what is already known.	4
Objectives	4	Provide an explicit statement of questions being addressed with reference to participants, interventions, comparisons, outcomes, and study design (PICOS).	5
**METHODS**			
Protocol and registration	5	Indicate if a review protocol exists, if and where it can be accessed (e.g., Web address), and, if available, provide registration information including registration number.	5
Eligibility criteria	6	Specify study characteristics (e.g., PICOS, length of follow-up) and report characteristics (e.g., years considered, language, publication status) used as criteria for eligibility, giving rationale.	5
Information sources	7	Describe all information sources (e.g., databases with dates of coverage, contact with study authors to identify additional studies) in the search and date last searched.	5
Search	8	Present full electronic search strategy for at least one database, including any limits used, such that it could be repeated.	Supplementary file 1
Study selection	9	State the process for selecting studies (i.e., screening, eligibility, included in systematic review, and, if applicable, included in the meta-analysis).	6
Data collection process	10	Describe method of data extraction from reports (e.g., piloted forms, independently, in duplicate) and any processes for obtaining and confirming data from investigators.	6
Data items	11	List and define all variables for which data were sought (e.g., PICOS, funding sources) and any assumptions and simplifications made.	6
Risk of bias in individual studies	12	Describe methods used for assessing risk of bias of individual studies (including specification of whether this was done at the study or outcome level), and how this information is to be used in any data synthesis.	6/7
Summary measures	13	State the principal summary measures (e.g., risk ratio, difference in means).	7
Synthesis of results	14	Describe the methods of handling data and combining results of studies, if done, including measures of consistency (e.g., I^2) for each meta-analysis.	7

For peer review only - http://bmjopen.bmj.com/site/about/guidelines.xhtml

PRISMA 2009 Checklist

Section/topic	#	Checklist item	Reported on page #
Risk of bias across studies	15	Specify any assessment of risk of bias that may affect the cumulative evidence (e.g., publication bias, selective reporting within studies).	7
Additional analyses	16	Describe methods of additional analyses (e.g., sensitivity or subgroup analyses, meta-regression), if done, indicating which were pre-specified.	N/A
**RESULTS**			
Study selection	17	Give numbers of studies screened, assessed for eligibility, and included in the review, with reasons for exclusions at each stage, ideally with a flow diagram.	7 and Figure 1
Study characteristics	18	For each study, present characteristics for which data were extracted (e.g., study size, PICOS, follow-up period) and provide the citations.	Table 1 and Table 2
Risk of bias within studies	19	Present data on risk of bias of each study and, if available, any outcome level assessment (see item 12).	Supplementary file 2
Results of individual studies	20	For all outcomes considered (benefits or harms), present, for each study: (a) simple summary data for each intervention group (b) effect estimates and confidence intervals, ideally with a forest plot.	8-14 and Figure 4
Synthesis of results	21	Present results of each meta-analysis done, including confidence intervals and measures of consistency.	8-14 and Figure 4
Risk of bias across studies	22	Present results of any assessment of risk of bias across studies (see Item 15).	N/A
Additional analysis	23	Give results of additional analyses, if done (e.g., sensitivity or subgroup analyses, meta-regression [see Item 16]).	N/A
**DISCUSSION**			
Summary of evidence	24	Summarize the main findings including the strength of evidence for each main outcome; consider their relevance to key groups (e.g., healthcare providers, users, and policy makers).	14/15
Limitations	25	Discuss limitations at study and outcome level (e.g., risk of bias), and at review-level (e.g., incomplete retrieval of identified research, reporting bias).	16/17
Conclusions	26	Provide a general interpretation of the results in the context of other evidence, and implications for future research.	15/16
**FUNDING**			
Funding	27	Describe sources of funding for the systematic review and other support (e.g., supply of data); role of funders for the systematic review.	18

From: Moher D, Liberati A, Tetzlaff J, Altman DG, The PRISMA Group (2009). Preferred Reporting Items for Systematic Reviews and Meta-Analyses: The PRISMA Statement. PLoS Med 6(6): e1000097.

For peer review only

For peer review only - http://bmjopen.bmj.com/site/about/guidelines.xhtml

PRISMA 2009 Checklist

doi:10.1371/journal.pmed1000097

For more information, visit: www.prisma-statement.org.

For peer review only

BMJ Open

Effect of interventions incorporating personalised cancer risk information on intentions and behaviour: a systematic review and meta-analysis of randomised controlled trials

Journal:	*BMJ Open*
Manuscript ID	bmjopen-2017-017717.R2
Article Type:	Research
Date Submitted by the Author:	20-Nov-2017
Complete List of Authors:	Usher-Smith, Juliet; The Primary Care Unit, Department of Public Health and Primary Care<br>Silarova, Barbora; MRC Epidemiology Unit,<br>Sharp, Stephen; University of Cambridge, MRC Epidemiology Unit<br>Mills, Katie; The Primary Care Unit, Department of Public Health and Primary Care<br>Griffin, Simon; The Primary Care Unit, Department of Public Health and Primary Care
**Primary Subject Heading**:	Communication
Secondary Subject Heading:	Oncology
Keywords:	ONCOLOGY, PREVENTIVE MEDICINE, PUBLIC HEALTH

SCHOLARONE™ Manuscripts

only

Effect of interventions incorporating personalised cancer risk information on intentions and behaviour: a systematic review and meta-analysis of randomised controlled trials

Juliet A Usher-Smith[1], Barbora Silarova[2], Stephen J Sharp[2], Katie Mills[1], Simon J Griffin[1]

[1] The Primary Care Unit, Department of Public Health and Primary Care, University of Cambridge School of Clinical Medicine, Box 113 Cambridge Biomedical Campus, Cambridge, CB2 0SR, UK

[2] MRC Epidemiology Unit, Institute of Metabolic Science, Cambridge, CB2 0QQ

Correspondence to: Juliet Usher-Smith jau20@medschl.cam.ac.uk

For peer review only

For peer review only - http://bmjopen.bmj.com/site/about/guidelines.xhtml

ABSTRACT

Objective To provide a comprehensive review of the impact on intention to change health-related behaviours and health-related behaviours themselves, including screening uptake, of interventions incorporating information about cancer risk targeted at the general adult population.

Design A systematic review and random effects meta-analysis

Data sources An electronic search of Medline, EMBASE, CINAHL and PsychINFO from 01/01/2000 to 01/07/2017.

Inclusions criteria Randomised controlled trials of interventions including provision of a personal estimate of future cancer risk based on two or more non-genetic variables to adults recruited from the general population including at least one behavioural outcome.

Results We included 19 studies reporting 12 outcomes. There was significant heterogeneity in interventions and outcomes between studies. There is evidence that interventions incorporating personalised cancer risk information do not affect intention to attend or attendance at screening (Relative risk (RR) 1.00 (0.97-1.03)). There is limited evidence that they increase smoking abstinence, sun protection, adult skin self-examination and breast examination and decrease intention to tan. However, they do not increase smoking cessation, parental child skin examination or intention to protect skin. No studies assessed changes in diet, alcohol consumption or physical activity.

Conclusions Interventions incorporating personalised cancer risk information do not affect uptake of screening but there is limited evidence of effect on some health-related behaviours. Further research, ideally including objective measures of behaviour, is needed before cancer risk information is incorporated into routine practice for health promotion in the general population.

For peer review only - http://bmjopen.bmj.com/site/about/guidelines.xhtml

Key words: Cancer risk, systematic review, intervention, prevention, communication, meta-analysis

For peer review only

For peer review only - http://bmjopen.bmj.com/site/about/guidelines.xhtml

Strengths and limitations of this study

- This systematic review is the first comprehensive review of the effect on intention and health-related behaviour of individuals in the general population of interventions delivered across multiple settings which incorporate personalised information about cancer risk.
- The use of a broad search strategy across multiple databases enabled us to identify 19 randomised controlled trials reporting the impact of interventions incorporating personalised cancer risk information on 12 outcomes.
- However, there was large heterogeneity across the studies, including the content of interventions and the outcome measures. This meant it was only possible to meta-analyse one outcome, attendance at screening, and in many studies separating the effect of the risk information alone from additional elements of the interventions was not possible.

For peer review only - http://bmjopen.bmj.com/site/about/guidelines.xhtml

INTRODUCTION

In 2006 the US National Cancer Institute recognised risk prediction models as an 'area of extraordinary opportunity'[1]. Since then an increasing number of risk prediction models have been developed. Such models can facilitate a personalised approach to cancer prevention and treatment and a more equitable and cost-effective distribution of finite resources by targeting screening and prevention activities at those most likely to benefit. Furthermore, being able to estimate, communicate and monitor individual risk and demonstrate the impact of lifestyle change on future risk of cancer may complement wider collective approaches to shifting population distributions of behaviour, risk factors and cancer risk.

Research has shown that many individuals have incorrect perceptions of their risk of cancer[2–4] and that both over- and under-estimation are associated with maladaptive health-related behaviours[5]. Additionally, whilst up to 40% of all cancers are attributable to lifestyle factors[6], only 3% of people are aware that being overweight can increase their risk of cancer and less than a third that physical activity could help reduce risk[7–10]. One in seven people additionally believe that lifetime risk of cancer is unmodifiable[11]. Most behaviour change theories suggest that perceived risk is important alongside other constructs such as self-efficacy, response efficacy in promoting behaviour change[12,13]. Providing individuals with estimates of their risk of cancer alongside other behaviour change interventions may therefore help motivate behaviour change at an individual level. It may also enable individuals to make more informed decisions about uptake of screening tests for cancer. This has led to the development of an increasing number of interventions incorporating information about cancer risk being developed.

For peer review only - http://bmjopen.bmj.com/site/about/guidelines.xhtml

Understanding the impact of interventions incorporating information about cancer risk on behaviour and intention to change behaviour before they are introduced into routine practice is important. Previous systematic reviews in this area have focused only on trials in primary care[14] or tailored information about cancer risk and screening[15,16]. In this review we aimed to provide a comprehensive synthesis of the impact of interventions incorporating personalised information about cancer risk on intention to change health-related behaviours and health-related behaviours within the general adult population.

METHODS

We performed a systematic literature review following an a priori established study protocol (available on request). Reporting followed the PRISMA statement[17].

Search strategy

We performed an electronic literature search of Medline, EMBASE, CINAHL and PsychINFO from January 2000 until July 2017 with no language limits using a combination of subject headings and free text incorporating 'cancer', 'risk/risk factor/risk assessment' and 'prediction/model/score/tool' (see Supplementary file 1 for the complete search strategies). We then extended the search by manually screening the reference lists of all included papers. We chose to begin the search in 2000 as the previous review of tailored information about cancer risk and screening had noted that computer delivered interventions, as would be required for calculating risk scores, were only described in publications from 2000 onwards[15].

Study selection

We included studies if they were randomised controlled trials (RCTs) published as a primary research paper in a peer-reviewed journal, included adults with no previous history of cancer

For peer review only

For peer review only - http://bmjopen.bmj.com/site/about/guidelines.xhtml

and included provision to individuals of a personal estimate of future cancer risk based on two or more non-genetic variables and reported at least one behavioural outcome. In order to focus on the provision of personalised cancer risk to the general population, we excluded studies which had recruited participants on the basis of a personal or family history of cancer or following referral to specialist cancer risk services. Vignette, before-and-after studies without a control group, cross-sectional, longitudinal and qualitative studies were also excluded along with conference abstracts, editorials, commentaries and letters.

Two reviewers (JUS and BS) each screened half of the titles and abstracts to exclude papers that were clearly not relevant. A third reviewer (SG) independently assessed a random selection of 5% of the papers screened by each of the first reviewers. The full text was examined if a definite decision to exclude could not be made based on title and abstract alone. Two reviewers (JUS and BS) independently assessed all full-text papers. We discussed papers for which it was unclear whether or not the inclusion criteria were met at consensus meetings with a third reviewer (SG). Papers written in languages other than English were translated into English for assessment and subsequent data extraction.

Data extraction

Two researchers (JUS+BS/KM) independently extracted data from studies included in the review using a standardized data abstraction form to reduce bias. The data extracted included: (1) Study characteristics (cancer type, study design, study setting, duration of follow-up); (2) selection of participants (inclusion criteria, method of recruitment/randomisation); (3) participant characteristics (age, level of cancer risk, sample size); (4) the intervention (risk tool used, method and format of risk communication, additional information or follow-up provided), and (4) measured outcome(s). Reviewers were not blinded to publication details.

For peer review only - http://bmjopen.bmj.com/site/about/guidelines.xhtml

Quality assessment

We conducted quality assessment at the same time as data extraction using a checklist based on the Critical Appraisal Skills Programme (CASP) guidelines[18] as an initial framework. This includes eight questions concerning whether the study addressed a clearly focused issue, the method of recruitment and randomisation, whether blinding was used, the measurement of the exposure and outcome, the comparability of the study groups and the follow-up. Each study was then classified as high, medium or low quality. No studies were excluded based on quality alone.

Data synthesis and statistical analysis

For analysis, we grouped the measured outcomes into those relating to: 1) preferences or intention to attend cancer screening; 2) cancer screening uptake; 3) intention or motivation to change health-related behaviour; and 4) change in health-related behaviour. It was only possible to pool results for screening attendance. For this we used random effects meta-analysis[19] and the 'metan' package in Stata. We present intervention effects as relative risk (RR) rather than odds ratios (OR) to avoid overestimating the risk[20]. We estimated the heterogeneity between studies using the I^2 statistic. All analyses were conducted using statistical software package Stata/SE version 12.

RESULTS

After duplicates were removed, the search identified 38,906 papers. Of these, 35,604 were excluded at title and abstract level and a further 183 after full-text assessment. After title and abstract screening by the first reviewers (JUS and BS), no additional papers met the inclusion criteria in the random 5% screened by the second reviewer (SG). The most common reasons

For peer review only - http://bmjopen.bmj.com/site/about/guidelines.xhtml

for exclusion at full-text level were that the papers did not include provision of a personal risk estimate (*n*=62), did not include any data on predefined outcomes (*n*= 37), were conference abstracts (*n*=20), or were not primary research (*n*=16) (Figure 1). Five further papers were identified through citation searching, giving 19 studies included in the analysis.

A summary of the participants and setting of those 19 studies is shown in Table 1. With the exception of three studies conducted in the UK[21–23], all studies took place in the USA. Most recruited participants from those attending primary care clinics (n=3), or from lists of potentially eligible individuals from electronic medical records (n=7), telephone services (n=1), insurance records (n=1) or survey companies (n=1). Two recruited through schools, community centres and universities, one from those calling a cancer information service and three used public advertisements.

In eight studies personalised information was provided about risk of breast cancer, in five about risk of colorectal cancer, in three risk of skin cancer, one lung cancer, one cervical cancer and one multiple cancers. Further details of the risk models used to calculate the risk estimate provided to participants and the format of the intervention(s) are given in Table 2. All eight studies providing personalised information about breast cancer risk used the Gail risk model[24]. This was the first risk model developed for breast cancer and includes age, age at menarche, age at first live birth, number of previous biopsies, number of biopsies showing atypical hyperplasia, and number of first-degree relatives with breast cancer. Where details were given (*n*=3), all studies on colorectal cancer used the Harvard Cancer Risk tool[25] which includes family history, height and weight, alcohol consumption, vegetable and red meat consumption, physical activity, screening history, a history of inflammatory bowel disease, and use of aspirin, folate and female hormones. Other risk models used were the Liverpool Lung

For peer review only - http://bmjopen.bmj.com/site/about/guidelines.xhtml

Project model[26], Family Healthware tool[27], Wilkinson score for cervical cancer[28] and the brief skin cancer risk assessment tool (BRAT)[29] adapted for children. Quality assessment for each of study is provided in Supplementary file 2. Seven were assessed as high or medium/high quality, 11 as medium quality and one as medium/low.

Overall findings and evidence synthesis along with the number and quality of studies addressing each outcome are summarised in Table 3.

Preferences and intentions for screening

Preferences for screening

Two RCTs reported participants' views about screening. In the cluster-randomised trial by Holloway *et al.*[21] participants who received a 10 minute counselling session including information about relative and absolute risks of cervical cancer integrated within a smear test appointment were significantly less likely to state a preference for the next interval for cervical screening to be 12 months or less than those who received usual care (OR: 0.51 (95%CI: 0.41-0.64)). The second study by Lipkus *et al.*[30] reported attitudinal ambivalence towards faecal occult blood test (FOBT) screening measured by their agreement with three Likert-style items stating that they had "mixed feelings", felt "torn" and had "conflicting thoughts" about whether to get screened for CRC using an FOBT. Participants who received personalised estimates of either absolute or absolute plus comparative risk alongside written information about CRC screening had significantly lower ambivalence than those who received the same written information without tailored CRC risk information ($p<0.05$).

Intention to attend cancer screening

For peer review only - http://bmjopen.bmj.com/site/about/guidelines.xhtml

Eight studies assessed intentions to attend cancer screening: five for mammography and four for CRC screening. Five showed no effect of risk information, three in which the only substantial difference between the intervention and control groups was the provision of a risk estimate[31–33]. Bodurtha *et al.*[31] found no significant differences at 18 months between those randomised to receive either printed sheets with their 5-year and lifetime estimates of breast cancer risk alongside information addressing barriers to mammography, breast cancer seriousness and benefits of yearly mammography, or general information about breast cancer prevention practices not tailored to their risk level (OR after adjusting for baseline intentions and recruitment site: 0.97 (95%CI: 0.70 to 1.33)). Davis *et al.*[34] reported that women who received a brief intervention over the telephone including information about lifetime risk of cancer and screening recommendations were no more likely at one month to report being in the maintenance stage (having had one mammogram in the past two years and two or more in the past four years and planning to get another on schedule) than the control group who received no intervention (67% in the intervention group compared to 68% in the control group). Helmes *et al.*[35] reported changes in a single breast health intentions measure which included intention to have mammography, clinical breast examination, and breast self-examination. They found no significant differences at baseline (p=0.23) or three month follow-up (p=0.46) between women who received estimates of their lifetime risk of breast cancer along with information about breast awareness either face-to-face or over the telephone and a control group who received no intervention. Schroy *et al.*[32] randomised participants to complete an interactive 20-30 minutes computer-based decision aid which either did or did not include a personalised risk assessment. There was no difference between groups on a five-point scale of how sure they were that they would schedule a CRC screening test (mean scores 4.3 (standard deviation (SD): 1.0) for both groups). Trevena *et al.*[33] similarly reported no effect on intention to have CRC screening of a 20-page decision aid including information about baseline risk and absolute

For peer review only - http://bmjopen.bmj.com/site/about/guidelines.xhtml

reduction in CRC mortality with screening, compared to a 3-page booklet with information and recommendations about screening.

The two studies reporting an effect were by Lipkus *et al.*[30] and Seitz *et al.*[36]. In Lipkus *et al.* intention to complete an FOBT that would be given to them within the following month was measured on a seven-point Likert scale. The intentions reported by participants who received absolute risk (mean 3.65, n=40) or absolute plus either low (mean 6.43, n=38) or high (mean 6.65, n=39) comparative risk information were statistically significantly higher ($p<0.05$) than those participants in the control group who were provided with the same written information but without risk estimates (mean 2.21, n=43). The mean intention reported by the group which received the comparative risk was also significantly higher than for the absolute risk only group. In Seitz *et al.* women were separated into those with an estimated 10-year breast cancer risk above or below 1.5%. Intention to wait until age 50 before undergoing a mammogram was measured for those with a risk <1.5% and intention to start or continue to undergo mammograms in their 40s for those with a risk ≥1.5. In the low risk group, all risk-based intervention conditions resulted in a significant increase in the percentage of women planning to wait to age 50. However, in the high risk group no such significant difference was seen.

The eighth study by Lipkus *et al.*[37] reported the difference in intentions to get a mammogram between one group that received a one-page handout including their estimated absolute risk and another group that received the same handout plus information concerning how their risk compared to a woman of their age and race at the lowest level of risk. Immediately after the provision of risk information, overall 2.5%, 67.8%, and 24.8% reported that the risk information lowered, did not affect, or increased their intentions to undergo a mammogram respectively, with no differences between the groups.

For peer review only - http://bmjopen.bmj.com/site/about/guidelines.xhtml

Attendance at screening

Twelve RCTs reported attendance at screening: six for mammography[31,34,38–41]; five for colorectal cancer[30,32,33,38,42]; and one for cervical cancer[21]. Except for one high quality RCT in which the intervention group received information sheets including general information on breast cancer risk alongside personalised risk information and telephone counselling and the offer for more intensive group or genetic counselling[41], all showed no effect of the risk-based interventions as shown in the meta-analysis (Figure 2) with a combined RR of 1.02 (95%CI: 0.98-1.03, I^2: 61.6%).

Intention to change health-related behaviours

Intention to tan or protect skin

One RCT by Greene and Brinn measured intention to tan on a six-item Likert-type scale and intention to protect skin using a three-item scale[43]. Participants who completed a self-assessment risk score alongside receiving generic information about tanning, tanning beds and sun exposure reported significantly decreased intentions to use tanning beds than those receiving the same generic information alone (2.68, n=70 compared to 3.19, n=71, $p<0.05$). In contrast there were no significant differences in intentions to protect skin (2.38, n=70 compared to 2.49, n=71, $p>0.05$).

Change in health-related behaviours

Smoking status

One high quality RCT[23] reported the impact of risk information on smoking status. Receiving a personalised risk estimate in addition to a generic leaflet did not predict self-report smoking

For peer review only - http://bmjopen.bmj.com/site/about/guidelines.xhtml

status at six months in current smokers (p=0.66) but was associated with an increased odds of remaining a former smoker in those who had recently quit (OR 1.91 (95%CI 1.03-3.55)).

Sun exposure and sun protection habits

Two RCTs[22,44] measured sun protection habits by survey completion at baseline and follow up. One by Glanz *et al.* compared the effect on childhood sun exposure and sun protection habits of three mailings with personalised risk feedback, interactive skin cancer education materials and a family fun guide to a single mailing of standardised skin cancer information[44]. The other by Glazebrooke *et al.* compared usual care with a self-directed computer program including individualised feedback of risk alongside sections on skin protection, how to detect melanoma, dangers of sun exposure, how to check skin and how to reduce risk[22]. Both showed increases in overall sun protection habits (increase in sun protection habits index 0.19 in the intervention group compared to 0.14, p=0.02[44] and mean difference in skin protective behaviour score between intervention and control at six month follow-up 0.33 (95% CI 0.09, 0.57) [22]) with variable results for individual aspects including wearing a sun hat, wearing a shirt, wearing sunglasses, use of sun cream, number of sunburns, staying in the shade, and sun exposure during weekdays and weekends.

Tanning bed usage

The RCT by Greene and Brinn[43] measured change in tanning behaviour and tanning bed usage. Participants who completed the self-assessment risk score reported lower rates of tanning bed usage in the previous month at follow-up (2.18, n=70 compared to 3.76, n=71, p<0.05) but no difference in change in tanning behaviour from pre- to post-intervention (-1.25, n=70 compared to -2.08, n=71, p>0.05).

For peer review only - http://bmjopen.bmj.com/site/about/guidelines.xhtml

Self/parent skin examination

The two RCTs by Glanz et al. and Glazebrooke et al., measured rates of skin examination in adults[22] or parents and children[44]. Both showed statistically significant increases among adults and parents receiving personalised risk information (p<0.05) while the increase in parents examining their children was not statistically significant (p=0.06).

Clinical breast examination and breast self-examination

Three RCTs[31,40,41] measured rates of clinical breast examination and/or breast self-examination following provision of risk information. In the RCT by Bodurtha *et al.*, no significant differences were seen between those randomised to receive printed sheets including estimates of 5-year and lifetime risk of breast cancer alongside information addressing barriers to mammography, breast cancer seriousness and benefits of yearly mammography and those receiving general information about breast cancer prevention practices not tailored to their risk level for either frequency of clinical breast examination (crude rates: 91.4% vs 91.0%; adjusted OR: 1.00 (95%CI: 0.60 to 1.66)) or breast self-examination (crude rates: 56.8% vs 57.6%; adjusted OR: 0.95 (95%CI: 0.67 to 1.33)[31]. The other two studies, both by Bowen *et al.*, found significantly (p<0.01) greater increases in the proportion reporting performing breast self-examination in the intervention groups (35% to 52% and 36% to 62%) compared with controls (33% to 36% and 38% to 40%)[40,41]. However, both these studies compared intensive interventions (four weekly 2-hour sessions led by a health counsellor[40] or information sheets plus telephone counselling and the offer of more intensive group or genetic counselling[41]) with delayed intervention.

DISCUSSION

This systematic review is, to our knowledge, the first review of the impact of interventions

For peer review only - http://bmjopen.bmj.com/site/about/guidelines.xhtml

delivered across multiple settings which incorporate personalised information about cancer risk on intention to change health-related behaviour and health-related behaviours themselves in the general population. The findings show that such interventions do not affect intention to attend or attendance at screening. There is limited evidence that they increase smoking abstinence, sun protection, adult skin self-examination and breast examination and decrease intention to tan. However, this was not seen for smoking cessation, parental child skin examination or intention to protect skin. There is a notable absence of studies assessing the impact on diet, physical activity and alcohol consumption with only one reporting smoking status and none including objective measures of behaviour.

Our finding that interventions incorporating personalised information about cancer risk had no effect on intention to attend or attendance at screening is consistent with a previous Cochrane review in which personalised risk communication had little effect on the uptake of screening tests (fixed-effect OR 0.95 (95% CI 0.78 to 1.15))[16]. However, as in that review, there was evidence of increased concordance between screening preferences and recommendations and decreased ambivalence. This supports the suggestion made in that review that personalised risk information might be useful for shared and informed decision making. For example, in surveys of participants about their knowledge and values for cancer screening decisions and decision-making processes, only 21% report feeling extremely well informed[45] and the majority overestimate lifetime risk of cancer incidence and mortality[45,46]. While providing individuals with information about their estimated cancer risk may therefore not influence overall rates of screening it may contribute to the decision to take up screening or not at an individual level and support shared decision making.

The absence of significant effects on health-related behaviours is also consistent with research

For peer review only - http://bmjopen.bmj.com/site/about/guidelines.xhtml

in other disease areas, such as cardiovascular disease, where systematic reviews have found only few studies reporting behaviour change and no significant effects on lifestyle[47–49]. This is perhaps not surprising given that behaviour change is influenced by many other factors, including health beliefs, social context, the environment, and personal attributes such as time orientation[12,13]. However, there was no evidence that interventions that include information about cancer risk result in harm through false reassurance and the adoption of unhealthy behaviours. This is important as on average many of the general population overestimate their own risk of cancer[30,35,40,50–52] and so if information about cancer risk were routinely provided within clinical practice large numbers would be receiving an estimate lower than their prior perceptions.

The main strengths of this review are the systematic search of multiple electronic databases and the broad inclusion criteria. This allowed us to include studies that assess the impact of interventions incorporating personalised cancer risk information on multiple behavioural outcomes. However, from nearly 40,000 titles and abstracts, we only included 14 with an additional 5 found through citation searching. This highlights the challenge in identifying studies in this area in which the primary purpose may not be related to the provision of personalised risk information. There was also significant heterogeneity in the outcome measures included, duration of follow-up and method of recruitment across the included studies. For all outcomes except attendance at screening there were either too few studies to meaningfully pool results or each study used different non-comparable measures. Even for attendance at screening for which meta-analysis was possible, we were only able to pool crude estimates and the included studies addressed screening for breast, bowel and cervical cancer. While it is possible that the impact on screening attendance might be different across the different cancer sites because of the nature of the tests involved, the finding that only one study

For peer review only - http://bmjopen.bmj.com/site/about/guidelines.xhtml

of mammography showed an effect of interventions incorporating personalised cancer risk information suggests that this is unlikely to be the case. The duration of follow-up also varied from 1 to 18 months. However, the studies with shorter follow-up were those with intention as the outcome measures and, of the 10 studies reporting health-related behaviours, five had a follow-up period of a year or more and three a period of six months. It is therefore unlikely that the studies as a whole were too short to detect changes in behaviour or reflected only immediate un-sustained changes.

A further limitation is that many of the interventions consisted of provision of personalised risk information alongside a range of additional information, either written or delivered in person or in groups. Separating the effect of the risk information from those additional elements of the interventions was therefore not possible. However, we chose not to exclude these studies from this review because it is unlikely that personalised risk information would be incorporated into routine practice in isolation and, if anything, including them would overestimate the effect of the personalised risk information. It is also possible that the findings do not reflect the potential impact of interventions incorporating personalised information about cancer risk on the general population as a whole: half of the included studies focused on female cancers and so only recruited women and all were subject to recruitment bias with the participants who agreed to take part potentially more interested in their cancer risk or more healthy, resulting in a bias in either direction.

In addition to these specific limitations of our review, the findings also suggest a number of areas for future research. In particular, the absence of studies assessing the impact on diet, physical activity and alcohol consumption, and only one study reporting smoking cessation, demonstrate the need for trials assessing change in these behaviours, preferably measured

For peer review only

objectively, including measures of other theory based determinants of behaviour change (for example, self-efficacy). Only with such data will we be able to assess whether such individualised approaches have a place alongside population-wide prevention strategies.

ACKNOWLEDGEMENTS

The authors thank Isla Kuhn, Reader Services Librarian, University of Cambridge Medical Library, for her help developing the search strategy.

Contributors

JUS developed the protocol, completed the search, screened articles for inclusion, extracted data, synthesized the findings, interpreted the results and drafted the manuscript. BS developed the protocol, screened articles for inclusion, extracted data, interpreted the results and critically revised the manuscript. SS synthesized the findings and critically revised the manuscript. KM extracted data, interpreted the results and critically revised the manuscript. SJG developed the protocol, screened articles for inclusion, interpreted the results and critically revised the manuscript. All authors approved the final version.

Funding

JUS and KM are funded by a Cancer Research UK/BUPA Foundation Cancer Prevention Fellowship (C55650/A21464). BS was supported by the Medical Research Council [MC_UU_12015/4]. SJS is supported by the Medical Research Council www.mrc.ac.uk [Unit Programme number MC_UU_12015/1]. The University of Cambridge has received salary support in respect of SJG from the NHS in the East of England through the Clinical Academic Reserve. The views expressed are those of the authors and not necessarily those of the NHS or Department of Health.

For peer review only - http://bmjopen.bmj.com/site/about/guidelines.xhtml

All researchers were independent of the funding body and the funder had no role in data collection, analysis and interpretation of data; in the writing of the report; or decision to submit the article for publication.

Data sharing

All data are available from the reports or authors of the primary research. No additional data is available.

Competing Interests

All authors have completed the Unified Competing Interest form at *www.icmje.org/coi_disclosure.pdf* (available on request from the corresponding author) and declare that (1) they have no support from or relationships with companies that might have an interest in the submitted work in the previous 3 years; (2) their spouses, partners, or children have no financial relationships that may be relevant to the submitted work; and (3) they have no non-financial interests that may be relevant to the submitted work.

The corresponding author has the right to grant on behalf of all authors and does grant on behalf of all authors, an exclusive licence on a worldwide basis to the BMJ Publishing Group Ltd and its Licensees to permit this article (if accepted) to be published in BMJ editions and any other BMJPGL products and sub-licences to exploit all subsidiary rights, as set out in our licence (http://resources.bmj.com/bmj/authors/checklists-forms/licence-for-publication).

All authors had full access to all of the data in the study and can take responsibility for the integrity of the data and the accuracy of the data analysis

The corresponding author affirms that the manuscript is an honest, accurate, and transparent account of the study being reported; that no important aspects of the study have been omitted; and that any discrepancies from the study as planned (and, if relevant, registered) have been explained.

For peer review only - http://bmjopen.bmj.com/site/about/guidelines.xhtml

FIGURE LEGENDS

Figure 1. PRISMA flow diagram

Figure 2. Relative risk for adherence to recommended screening post intervention. CRC – colorectal cancer; FOBT – faecal occult blood test; AR – absolute risk; CR – comparative risk

REFERENCES

1 National Cancer Institute. The nation's investment in cancer research. A plan and budget proposal for the fiscal year 2006. 2006.

2 McCaul KD, O'Donnell SM. Naive beliefs about breast cancer risk. *Women's Heal* 1998;**4**:93–101.

3 Emery J, Morris H, Goodchild R, *et al.* The GRAIDS Trial: a cluster randomised controlled trial of computer decision support for the management of familial cancer risk in primary care. *Br J Cancer* 2007;**97**:486–93.

4 Weinstein ND, Atwood K, Puleo E, *et al.* Colon Cancer: Risk Perceptions and Risk Communication. *J Health Commun* 2004;**9**:53–65.

5 Waters EA, Klein WM, Moser RP, *et al.* Correlates of unrealistic risk beliefs in a nationally representative sample. *J Behav Med* 2011;**34**:225–35.

6 Parkin DM, Boyd L, Walker LC. The fraction of cancer attributable to lifestyle and environmental factors in the UK in 2010. *Br J Cancer* 2011;**105 Suppl**:S77-81.

7 Cancer Research UK. Perceptions of Risk Survey 2008 : Key Findings. 2008.

8 Grunfeld EA, Ramirez AJ, Hunter MS, *et al.* Women's knowledge and beliefs regarding breast cancer. *Br J Cancer* 2002;**86**:1373–8.

9 Redeker C, Wardle J, Wilder D, *et al.* The launch of Cancer Research UK's 'Reduce the

For peer review only

For peer review only - http://bmjopen.bmj.com/site/about/guidelines.xhtml

Risk' campaign: baseline measurements of public awareness of cancer risk factors in 2004. *Eur J Cancer* 2009;**45**:827–36.

10 Wardle J, Waller J, Brunswick N, *et al.* Awareness of risk factors for cancer among British adults. *Public Health* 2001;**115**:173–4.

11 Ryan AM, Cushen S, Schellekens H, *et al.* Poor Awareness of Risk Factors for Cancer in Irish Adults: Results of a Large Survey and Review of the Literature. *Oncologist* 2015;**20**:372–8.

12 Rogers R. A protection motivation theory of fear appeals and attitude change. *J Psychol* 1975;**91**:93–114.

13 Witte K. Putting the fear back into fear appeals: The extended parallel process model. *Commun Monogr* 1992;**59**:329–49.

14 Walker JG, Licqurish S, Pirotta M, *et al.* Cancer Risk Assessment Tools in Primary Care: A Systematic Review of Randomized Controlled Trials. 2015;:480–9.

15 Albada A, Ausems MGEM, Bensing JM, *et al.* Tailored information about cancer risk and screening: a systematic review. *Patient Educ Couns* 2009;**77**:155–71.

16 Edwards AGK, Evans R, Dundon J, *et al.* Personal risk communication for informed decision making about taking screening tests. *Cochrane Database Syst Rev 2006* Published Online First: 2006. doi:10.1002/14651858.CD001865.pub2

17 Moher D, Liberati A, Tetzlaff J, *et al.* Preferred reporting items for systematic reviews and meta-analyses: the PRISMA statement. *Int J Surg* 2010;**8**:336–41.

18 Critical Appraisal Skills Programme (CASP) Checklists. http://www.casp-uk.net/casp-tools-checklists

19 Hedges L V., Vevea JL. Fixed- and random-effects models in meta-analysis. *Psychol Methods* 1998;**3**:486–504.

20 Knol MJ, Le Cessie S, Algra A, *et al.* Overestimation of risk ratios by odds ratios in

For peer review only

For peer review only - http://bmjopen.bmj.com/site/about/guidelines.xhtml

trials and cohort studies: Alternatives to logistic regression. *CMAJ* 2012;**184**:895–9.

21 Holloway RM, Wilkinson C, Peters TJ, *et al.* Cluster-randomised trial of risk communication to enhance informed uptake of cervical screening. *Br J Gen Pract* 2003;**53**:620–5.

22 Glazebrook C, Garrud P, Avery A, *et al.* Impact of a multimedia intervention 'Skinsafe' on patients' knowledge and protective behaviors. *Prev Med (Baltim)* 2006;**42**:449–54.

23 Sherratt FC, Marcus MW, Robinson J, *et al.* Utilizing Lung Cancer Risk Prediction Models to Promote Smoking Cessation: Two Randomized Controlled Trials. *Am J Health Promot* Published Online First: 2016. doi:10.1177/0890117116673820

24 Gail MH, Brinton LA, Byar DP, *et al.* Projecting individualized probabilities of developing breast cancer for white females who are being examined annually. *J Natl Cancer Inst* 1989;**81**:1879–86.

25 Colditz G a, Atwood K a, Emmons K, *et al.* Harvard report on cancer prevention volume 4: Harvard Cancer Risk Index. Risk Index Working Group, Harvard Center for Cancer Prevention. *Cancer Causes Control* 2000;**11**:477–88.

26 Cassidy A, Duffy SW, Myles JP, *et al.* Lung cancer risk prediction: a tool for early detection. *Int J cancer* 2007;**120**:1–6.

27 Yoon PW, Scheuner MT, Jorgensen C, *et al.* Developing Family Healthware, a family history screening tool to prevent common chronic diseases. *Prev Chronic Dis* 2009;**6**:A33.

28 Wilkinson CE, Peters TJ, Stott NC, *et al.* Prospective evaluation of a risk scoring system for cervical neoplasia in primary care. *Br J Gen Pract* 1994;**44**:341–4.

29 Glanz K, Schoenfeld E, Weinstock MA, *et al.* Development and reliability of a brief skin cancer risk assessment tool. *Cancer Detect Prev* 2003;**27**:311–5.

30 Lipkus IM, Klein WMP. Effects of communicating social comparison information on

For peer review only - http://bmjopen.bmj.com/site/about/guidelines.xhtml

risk perceptions for colorectal cancer. *J Health Commun* 2006;**11**:391–407.

31 Bodurtha J, Quillin JMJ, Tracy K a, *et al.* Mammography screening after risk-tailored messages: the women improving screening through education and risk assessment (WISER) randomized, controlled trial. *J women's Heal* 2009;**18**:41–7.

32 Schroy PC, Emmons KM, Peters E, *et al.* Aid-assisted decision making and colorectal cancer screening: A randomized controlled trial. *Am J Prev Med* 2012;**43**:573–83.

33 Trevena L, Irwig L, Barratt A, *et al.* Randomized trial of a self-administered decision aid for colorectal cancer screening. *J Med Screen* 2008;**15**:76–82.

34 Davis S, Stewart S, Bloom J. Increasing the accuracy of perceived breast cancer risk: results from a randomized trial with Cancer Information Service callers. *Prev Med* 2004;**39**:64–73.

35 Helmes AW, Culver JO, Bowen DJ. Results of a randomized study of telephone versus in-person breast cancer risk counseling. *Patient Educ Couns* 2006;**64**:96–103.

36 Seitz HH, Gibson L, Skubisz C, *et al.* Effects of a risk-based online mammography intervention on accuracy of perceived risk and mammography intentions. *Patient Educ Couns* 2016;**99**:1647–56.

37 Lipkus IM, Biradavolu M, Fenn K, *et al.* Informing women about their breast cancer risks: truth and consequences. *Health Commun* 2001;**13**:205–26.

38 Rubinstein WS, Acheson LS, Neill SMO, *et al.* Clinical utility of family history for cancer screening and referral in primary care: A report from the Family Healthware Impact Trial. 2012;**13**:956–65.

39 Rimer BK, Halabi S, Sugg Skinner C, *et al.* Effects of a mammography decision-making intervention at 12 and 24 months. *Am J Prev Med* 2002;**22**:247–57.

40 Bowen DJ, Powers D, Greenlee H. Effects of Breast Cancer Risk Counseling for Sexual Minority Women. *Health Care Women Int* 2006;**27**:59–74.

For peer review only - http://bmjopen.bmj.com/site/about/guidelines.xhtml

41 Bowen DJ, Powers D. Effects of a mail and telephone intervention on breast health behaviors. *Health Educ Behav* 2010;**37**:479–89.

42 Sequist TD, Zaslavsky AM, Colditz GA, *et al.* Electronic patient messages to promote colorectal cancer screening: a randomized controlled trial. *Arch Intern Med* 2011;**171**:636–41.

43 Greene K, Brinn LS. Messages Influencing College Women's Tanning Bed Use: Statistical versus Narrative Evidence Format and a Self-Assessment to Increase Perceived Susceptibility. *J Health Commun* 2003;**8**:443.

44 Glanz K, Steffen AD, Schoenfeld E, *et al.* Randomized Trial of Tailored Skin Cancer Prevention for Children: The Project SCAPE Family Study. *J Health Commun* 2013;**18**:1368–83.

45 Hoffman RM, Elmore JG, Pignone MP, *et al.* Knowledge and values for cancer screening decisions: Results from a national survey. *Patient Educ Couns* 2016;**99**:624–30.

46 Hoffman RM, Lewis CL, Pignone MP, *et al.* Decision-Making Processes for Breast, Colorectal, and Prostate Cancer Screening: The DECISIONS Survey. *Med Decis Mak* 2010;**30**:53–64.

47 Usher-Smith JA, Silarova B, Schuit E, *et al.* Impact of provision of cardiovascular disease risk estimates to healthcare professionals and patients: a systematic review. *BMJ Open* 2015;**5**:e008717.

48 Willis A, Davies M, Yates T, *et al.* Primary prevention of cardiovascular disease using validated risk scores: a systematic review. *J R Soc Med* 2012;**105**:348–56.

49 Sheridan SL, Viera AJ, Krantz MJ, *et al.* The effect of giving global coronary risk information to adults: a systematic review. *Arch Intern Med* 2010;**170**:230–9.

50 Quillin JM, Fries E, McClish D, *et al.* Gail model risk assessment and risk perceptions. *J*

For peer review only

For peer review only - http://bmjopen.bmj.com/site/about/guidelines.xhtml

Behav Med 2004;**27**:205–14.

51 McCaul K, Canevello A, Mathwig J, *et al.* Risk communication and worry about breast cancer. *Psychol Health Med* 2003;**8**:379–89.

52 Lipkus IM, Klein WM, Rimer BK. Communicating breast cancer risks to women using different formats. *Cancer Epidemiol biomarkers Prev* 2001;**10**:895–8.

For peer review only

For peer review only - http://bmjopen.bmj.com/site/about/guidelines.xhtml

Table 1. Details of the setting and key outcomes of the included studies

Author, year	Cancer site(s)	Follow-up	Setting and participants	Risk level / co-morbidities	Outcome(s)	Quality*
Bodurtha 2009	Breast	18 months	899 women with no history of breast cancer recruited from waiting rooms of four women's health clinics	Not given	Mammography, clinical breast examinations, breast self-examination, mammography intentions	M-H
Bowen 2006	Breast	6 and 24 months	150 sexual minority women recruited via public advertisements	Mean Gail lifetime risk 12%	Breast self-examination, breast cancer screening	H
Bowen 2010	Breast	12 months	1,366 women recruited via purchased lists of telephone numbers with no previous diagnosis of breast cancer	Mean Gail lifetime risk 12%	Breast self-examination, mammography	
Davis, 2004	Breast	1 month	392 women with no history cancer calling the Cancer Information Service	27% 2-6% lifetime risk; 32% 6-9% lifetime risk; 41% 9-46% lifetime risk	Adherence to breast cancer screening, intention for breast cancer screening	M
Glanz 2013	Skin	16 weeks	Convenience sample of 1047 parents not currently being treated for skin cancer recruited through schools and community centres	38% high risk	Sun protection habits, sun exposure, skin examination by parents	M
Glazebrook 2006	Skin	6 months	589 recruited from 10 primary care practices from a convenience sample of appointments	Not given	Sun protection habits	M
Greene 2003	Skin	3-4 weeks	141 undergraduates at one university who received extra credit for participation	Not given	Intention to tan, actual tan bed usage	L-M
Helmes, 2006	Breast	3 months	Random sample of 340 members of state healthcare system with no history of breast/ovarian cancer or testing for cancer risk	Mean 9.5% (3.2) lifetime risk	Intention to have mammogram and clinical breast examination, intention to do breast self-examination	M
Holloway, 2003	Cervical	0, 4 years	1890 women attending routine cervical smear test at one of 29 GP practices	78-80% very low risk; 20-22% low risk	Preference for future screening interval, actual screening behaviour	M-H
Lipkus 2006	Colorectal	0	160 members of general public with no history of CRC or screening for CRC recruited through newspaper advertisements	Not given	Ambivalence, intention to screen using a FOBT, actual FOBT screening rates	M
Lipkus, 2001	Breast	0	121 members of general public recruited through newspaper advertisements	Mean 10 year risk 2.65% (SD 1.13)	Mammography screening and intentions	M
Rimer 2002	Breast	1 and 2 years	752 women aged 40-44 and 50-54 enrolled in a personal care plan	Mean 10 year risk 2.7%	Mammography	M
Rubenstein 2011	Breast, ovarian, colon	6 months	3786 patients from primary care clinic records with no history of colon, breast or ovaraian cancer invited by mail following record review	34% moderate or strong risk of ≥ 1 of the cancers	CRC screening, mammography	M

For peer review only - http://bmjopen.bmj.com/site/about/guidelines.xhtml

Schroy, 2011	Colorectal	0	666 patients due for bowel screening identified from monthly audits of one hospital's electronic medical record	Average	Preferences, satisfaction with the decision-making process, screening intentions, and test concordance	M-H
Schroy, 2012	Colorectal	0, 1, 3, 6 and 12 months	825 patients due for bowel screening identified from monthly audits of one hospital's electronic medical record	Average	Completion of a CRC screening test	H
Seitz 2016	Breast	0	2,918 women aged 35-49 with no history of breast cancer or a genetic mutation in BRCA1 or BRCA2 recruited through a survey company	42% 10 year risk <1.5% (mean 1.08 SD 0.01); 58% 10 year risk ≥1.5% (mean 2.53 SD 0.04)	Mammography intentions	M
Sequist 2012	Colorectal	1 and 4 months	1,103 patients from 14 ambulatory health centres who were overdue for colorectal cancer screening	Average	CRC screening	M
Sherratt 2016	Lung	6 months	297 current and 216 recent former smokers aged 18-60 without a history of lung cancer and attending smoking cessation services	Not given	Smoking status	H
Trevena 2008	Colorectal	1 month	314 patients recruited from 6 primary care practices without a history of colorectal cancer	Not given	Screening intentions, CRC screening	M

RCT – randomised controlled trial; CRC – colorectal cancer; CT computerised tomography; FOBT – faecal occult blood test
* L – low, M – medium, H - high

For peer review only - http://bmjopen.bmj.com/site/about/guidelines.xhtml

Table 2. Details of the risk-based interventions in each of the included studies

Author, year	Risk tool	Intervention group(s)	Comparison (where applicable)	Format of risk
Bodurtha 2009	Gail model (5 year and lifetime)	Information sheets with risk level and handouts addressing traditional constructs of Health Belief Model including barriers to mammography, breast cancer seriousness, individual risk for breast cancer, and benefits of yearly mammography	General information about breast cancer prevention practices, including mammography	Usual (<15%), Moderate (15-30%) or Strong (>30%)
Bowen 2006	Gail model (5 year, 10 year and at age 79)	Four weekly 2-hour sessions led by a health counsellor focusing on risk assessment and education, screening, stress management and social support	Delayed intervention	No details given
Bowen 2010	Gail model (lifetime)	Information sheets with general information on breast cancer risk and personalised risk information plus telephone counselling and offer for more intensive group or genetic counselling	Delayed intervention	Bar graph of absolute lifetime risk along with age-appropriate estimates for the "average risk" woman
Davis, 2004	BRCA tool (updated version of Gail model) (lifetime)	10min brief intervention designed to increase accuracy of perceived risk including results of risk assessment and screening recommendations tailored to participant's stage of adoption of mammography and follow up written information	No intervention	Verbal over the telephone. No additional details given.
Glanz 2013	Children's BRAT	Three mailings with personalised risk feedback, interactive skin cancer education materials, a family fun guide and suggestions for overcoming barriers and reminders to engage in preventive practices	Single mailing of standardised skin cancer information	No details given
Glazebrooke 2006	No details given	Self-directed computer program including sections on skin protection, how to detect melanoma, dangers of sun exposure, how to check skin, how to reduce risk and individualized feedback of risk	Usual care	Comparative risk
Greene 2003	Relative risk adapted from "ADD Wants to Convert"	Self-assessment of risk alongside generic messages about tanning, tanning beds and sun exposure	Generic messages about tanning, tanning beds and sun exposure	Numerical scale from 1-36
Helmes, 2006	Gail model (lifetime)	Face-to-face or telephone intervention consisting of 8 items: 1) a personal risk sheet ; 2) a personal computer-drawn pedigree; 3) a 23 page participant booklet; 4) Breast self-examination brochure; 5) Pap smear and mammography	No intervention	Bar charts of absolute % risk with numerical % alongside for the individual, an average-risk woman, and a high-risk woman

For peer review only - http://bmjopen.bmj.com/site/about/guidelines.xhtml

		brochure; 6) BSE shower card; 7) pictures of chromosomes and gene mutations; 8) a list of community resources for breast cancer		
Holloway, 2003	Wilkinson score	Brief 10 minute counselling session integrated with smear test appointment including relative and absolute risks and then negotiation of appropriate screening intervals	Usual care	Comparative and absolute risk in pictures and numbers
Lipkus 2006	Not given	Written information about CRC, CRC screening methods and CRC risk factors plus either 1) tailored CRC risk factor information or 2) tailored CRC risk factor information plus information on whether their total number of CRC risk factors was greater or not than average	Written information about CRC, CRC screening methods, and CRC risk factors	Narrative comparative risk
Lipkus, 2001	Gail model (10 year)	1 page handout describing the Gail model plus absolute risk alone	As for intervention group plus how their risk compared to a woman of their age and race at the lowest level of risk	Absolute risk +/- risk of a woman at the lowest level of risk as percentages in a pie chart
Rimer 2002	Gail model (10 year and lifetime)	Tailored print booklet and brief tailored newspaper plus personalized risk	Usual care (postcard reminder)	Absolute risk as a percentage
Rubenstein 2011	Family Healthware tool	Written personalized risk assessment and tailored prevention messages	Written generalized prevention messages	Qualitative risk - weak, moderate or strong familial risk
Schroy, 2011	Harvard cancer risk model (10 year)	Interactive 20-30 min computer-based decision aid plus personalized risk assessment	Interactive 20-30 min computer-based decision aid alone	Thermograph, indicating where the participant is along with a description e.g. your risk is below average
Schroy, 2012	Harvard cancer risk model (10 year)	Interactive 20-30 min computer-based decision aid plus personalized risk assessment followed immediately by a meeting with their providers to discuss screening and identify a preferred screening strategy. Providers received written notification hand-delivered by all the patients acknowledging that they were participating in the “CRC decision aid study” at the time of the visit to ensure that screening was discussed	As for intervention but without personalized risk assessment	Qualitative framing (“very much below average risk” to “very much above average risk”) with accompanying suggestions for behaviour modifications that might reduce risk, including a strong recommendation for screening, regardless of risk
Seitz 2016	Gail model (10 year)	Online risk plus basic information about mammography and national recommendations plus either 1) statements about women making choices 2) untailored exemplars of women making choices or 3) exemplars of similar women making choices	No information or the same basic information as intervention group	Absolute risk and risk of an average-risk age-matched women as numeric frequencies and icon arrays

For peer review only - http://bmjopen.bmj.com/site/about/guidelines.xhtml

Sequist 2011	Harvard cancer risk model (10 year)	Personalized electronic message highlighting their overdue screening status and providing a link to a web-based tool to assess their risk	No contact	Comparative risk on 7-point ordinal scale from very-much below average to very-much above average and in interactive graphical format
Sherratt 2016	Liverpool Lung Project model (5 year at age 70)	Personalised risk plus booklet stating the association between smoking and lung cancer and highlighting that quitting smoking was the best thing to do	As for intervention but without personalized risk assessment	Verbal and written absolute risk if continue to smoke and if stop smoking alongside icon arrays
Trevena 2008	No details given	20 page booklet including personalized risk, absolute reduction in colorectal cancer mortality with screening over the next 10 years, probability of test outcomes from screening and information about how to get screeed.	3 page booklet with information and recommendations about screening	Words and 1000-face diagrams

CRC – colorectal cancer

For peer review only - http://bmjopen.bmj.com/site/about/guidelines.xhtml

Table 3. Summary of evidence on outcomes

Outcome measure	Number of studies	Studies with significant positive effect	Studies with no effect	Best evidence synthesis
Screening				
Preferences for screening	2	1 medium/high quality and 1 high quality RCT	None	Evidence of positive effect
Intention to attend screening	8	1 medium quality RCT*	1 high quality, 1 medium/high quality and 4 medium quality RCTs*	Evidence of no effect
Attendance at screening	12	1 high quality RCT	2 high quality, 2 medium/high quality and 7 medium quality studies	Evidence of no effect
Health-related behaviours				
Intention to change health-related behaviours				
To tan	1	1 low/medium RCT	None	Limited evidence of positive effect
To protect skin	1	None	1 low/medium RCT	Limited evidence of no effect
Health-related behaviours				
Smoking cessation	1	None	1 high quality RCT	Limited evidence of no effect
Smoking abstinence	1	1 high quality RCT	None	Limited evidence of positive effect
Sun protection	2	2 medium quality RCTs		Indicative evidence of positive effect
Tanning bed usage	1	None	1 low/medium RCT	Limited evidence
Adult skin examination	2	2 medium quality RCTs	None	Indicative evidence of positive effect
Child skin examination	1	None	1 medium quality RCT	Limited evidence of no effect
Breast examination	3	2 high quality RCTs	1 medium/high RCT	Indicative evidence of positive effect
Diet	0	None	None	No evidence
Physical activity	0	None	None	No evidence
Alcohol	0	None	None	No evidence

* 1 medium quality study reported a significant positive effect in low risk women and no effect in high risk women

For peer review only - http://bmjopen.bmj.com/site/about/guidelines.xhtml

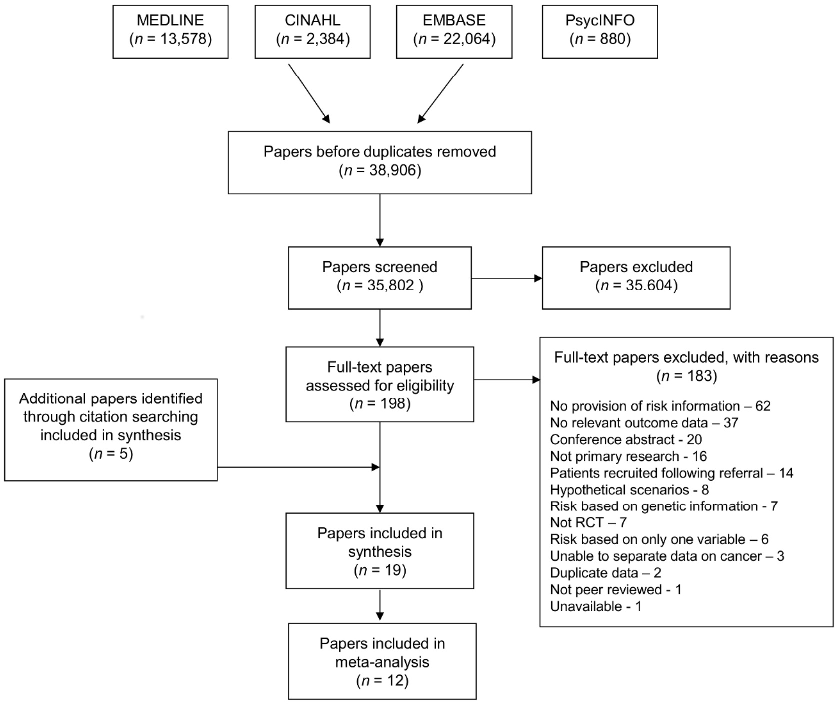

Figure 1. PRISMA flow diagram

254x190mm (300 x 300 DPI)

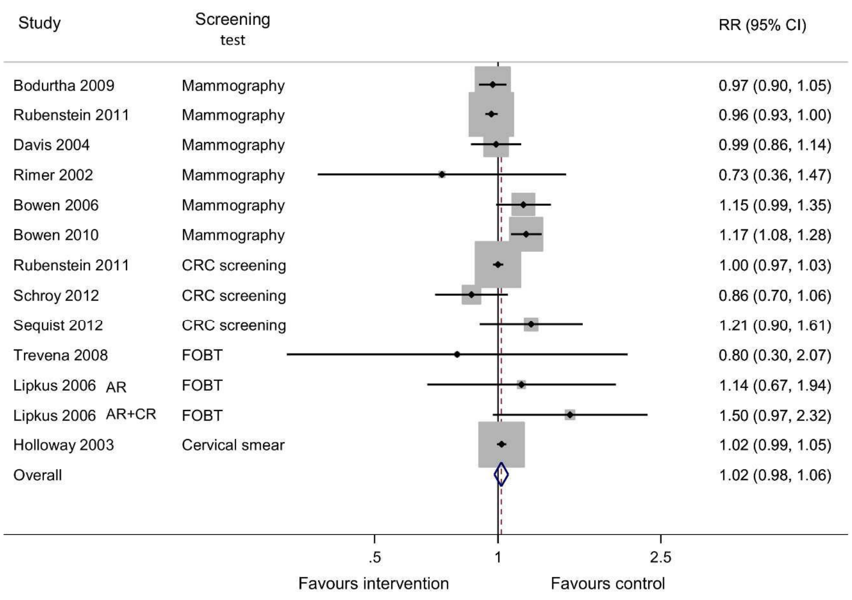

Figure 2. Relative risk for adherence to recommended screening post intervention. CRC – colorectal cancer; FOBT – faecal occult blood test; AR – absolute risk; CR – comparative risk

254x190mm (300 x 300 DPI)

For peer review only - http://bmjopen.bmj.com/site/about/guidelines.xhtml

Supplementary file 1 – Complete search strategy

Medline and Cinahl

S28	S26 NOT S27
S27	review
S26	S24 AND S25
S25	S13 NOT S15
S24	S14 OR S16 OR S17 OR S21 OR S22 OR S23
S23	(behaviour OR behavior) AND health
S22	(MH "Health Behavior+") OR (MH "Risk Reduction Behavior+")
S21	S18 OR S20
S20	S19 AND S1
S19	screen* AND uptake OR attendance OR intention OR adherence
S18	(MM "Early Detection of Cancer/UT")
S17	anxiety* OR worry* OR denial* OR hopelessness* OR avoidance*
S16	efficacy OR effectiv*
S15	PT review OR PT letter OR PT comment OR PT editorial
S14	percep* OR perceive* OR understand* OR understood* OR accura* OR comprehen*
S13	S9 NOT S12
S12	S10 OR S11
S11	(MH "Prognosis+")
S10	prognos* OR treatment* OR surgery*
S9	S1 AND S8
S8	S6 OR S7
S7	(MH "Risk Assessment+")
S6	S4 AND S5
S5	score* OR model* OR predict* OR tool*
S4	S2 OR S3
S3	(MH "Risk+")
S2	risk*
S1	"cancer" OR (MH "Neoplasms+")

Embase

1	cancer.mp. or exp neoplasm/
2	exp risk/ or risk*.mp.
3	(score* or model* or predict* or tool*).mp. [mp=title, abstract, heading word, drug trade name, original title, device manufacturer, drug manufacturer, device trade name, keyword]
4	2 and 3
5	exp risk assessment/
6	4 or 5
7	1 and 6
8	(percep* or perceive* or understand* or understood* or accura* or comprehen*).mp. [mp=title, abstract, heading word, drug trade name, original title, device manufacturer, drug manufacturer, device trade name, keyword]
9	(efficacy* or effectiv*).mp. [mp=title, abstract, heading word, drug trade name, original title, device manufacturer, drug manufacturer, device trade name, keyword]
10	exp prognosis/
11	(prognos* or treatment* or surgery*).mp. [mp=title, abstract, heading word, drug trade name, original title, device manufacturer, drug manufacturer, device trade name, keyword]

For peer review only

12 (review or letter or comment or editorial).pt.
13 (radiotherapy* or stage* or grade*).mp. [mp=title, abstract, heading word, drug trade name, original title, device manufacturer, drug manufacturer, device trade name, keyword]
14 (anxiety* or worry* or fatalism* or hopelessness* or denial* or avoid*).mp. [mp=title, abstract, heading word, drug trade name, original title, device manufacturer, drug manufacturer, device trade name, keyword]
15 8 or 9 or 14
16 10 or 11 or 12 or 13
17 exp cancer screening/
18 health behaviour.mp. or exp health behavior/
19 ((behaviour or behavior) and health).mp. [mp=title, abstract, heading word, drug trade name, original title, device manufacturer, drug manufacturer, device trade name, keyword]
20 (screen* and (uptake or attendance or intention or adherence)).mp. [mp=title, abstract, heading word, drug trade name, original title, device manufacturer, drug manufacturer, device trade name, keyword]
21 20 and 1
22 15 or 17 or 18 or 19 or 21
23 22 and 7
24 23 not 16
25 limit 24 to yr="2000 -Current"
26 25 not review.mp. [mp=title, abstract, heading word, drug trade name, original title, device manufacturer, drug manufacturer, device trade name, keyword]

PsycInfo
S20 S19 NOT review Limiters - Publication Year: 2000-2015
S19 S17 NOT (S10 OR S11 OR S12)
S18 S17 NOT (S10 OR S11 OR S12)
S17 S7 and (S8 or S9 or S13 or S15 or S16)
S16 health AND (behaviour OR behavior)
S15 S14 AND S1
S14 screen* AND (uptake OR attendance OR intention OR adherence)
S13 MM "Cancer Screening"
S12 (prognos* OR treatment* OR surgery*) AND (S10 OR S11)
S11 prognos* OR treatment* OR surgery*
S10 DE "Prognosis"
S9 efficacy or effectiv* or worry* or anxiety* or hopelessness* or denial*
S8 percep* OR perceive* OR understand* OR understood* OR accura* OR comprehen*
S7 (S1 AND S6)
S6 (S4 OR S5)
S5 DE "Risk Assessment"
S4 (S2 AND S3)
S3 score* OR model* OR predict* OR tool*
S2 risk*
S1 DE "Neoplasms" OR DE "Benign Neoplasms" OR DE "Breast Neoplasms" OR DE "Endocrine Neoplasms" OR DE "Leukemias" OR DE "Nervous System Neoplasms" OR DE "Terminal Cancer"

For peer review only

Supplementary file 2. Quality assessment of included studies

Author, date	Study addressed a clearly focused issue	Randomisation	Recruitment / comparability of study groups at baseline	Blinding	Exposure measurement	Outcome measurement	Comparability of study groups during study	Follow up	Overall
Bodurtha, 2009	●	●	•	·	●	●	•	•	M-H
Bowen 2006	●	●	●	·	●	●	●	•	H
Bowen 2010	●	●	●	·	●	●	●	•	H
Davis, 2004	●	●	•	·	●	●	•	·	M
Glanz, 2013	●	●	•	•	•	•	•	●	M
Glazebrook 2006	●	●	•	·	•	•	•	·	M
Greene, 2003	•	•	·	·	•	•	·	·	L-M
Helmes, 2006	●	•	•	·	•	•	•	•	M
Holloway, 2003	●	●	●	•	●	●	•	·	M-H
Lipkus , 2006	●	●	·	·	•	•	·	●	M
Lipkus, 2001	●	•	·	·	n/a	•	•	•	M
Rimer 2002	●	●	•	·	•	●	•	•	M
Rubenstein, 2011	●	●	●	·	●	•	●	●	M
Schroy, 2011	●	●	●	·	●	●	●	•	M-H
Schroy, 2012	●	●	●	·	●	●	●	●	H
Seitz 2016	•	•	•	·	•	•	•	·	M

For peer review only

Sequist 2011	M	M	M	L	M	M	M	n/a	M
Sherratt 2016	H	H	H	M	M	M	H	H	H
Trevena 2008	M	M	H	H	M	M	M	M	M

• Low (L) ● Medium (M) ⬤ High (H)

For peer review only

PRISMA 2009 Checklist

Section/topic	#	Checklist item	Reported on page #
**TITLE**			
Title	1	Identify the report as a systematic review, meta-analysis, or both.	1
**ABSTRACT**			
Structured summary	2	Provide a structured summary including, as applicable: background; objectives; data sources; study eligibility criteria, participants, and interventions; study appraisal and synthesis methods; results; limitations; conclusions and implications of key findings; systematic review registration number.	2
**INTRODUCTION**			
Rationale	3	Describe the rationale for the review in the context of what is already known.	4
Objectives	4	Provide an explicit statement of questions being addressed with reference to participants, interventions, comparisons, outcomes, and study design (PICOS).	5
**METHODS**			
Protocol and registration	5	Indicate if a review protocol exists, if and where it can be accessed (e.g., Web address), and, if available, provide registration information including registration number.	5
Eligibility criteria	6	Specify study characteristics (e.g., PICOS, length of follow-up) and report characteristics (e.g., years considered, language, publication status) used as criteria for eligibility, giving rationale.	5
Information sources	7	Describe all information sources (e.g., databases with dates of coverage, contact with study authors to identify additional studies) in the search and date last searched.	5
Search	8	Present full electronic search strategy for at least one database, including any limits used, such that it could be repeated.	Supplementary file 1
Study selection	9	State the process for selecting studies (i.e., screening, eligibility, included in systematic review, and, if applicable, included in the meta-analysis).	6
Data collection process	10	Describe method of data extraction from reports (e.g., piloted forms, independently, in duplicate) and any processes for obtaining and confirming data from investigators.	6
Data items	11	List and define all variables for which data were sought (e.g., PICOS, funding sources) and any assumptions and simplifications made.	6
Risk of bias in individual studies	12	Describe methods used for assessing risk of bias of individual studies (including specification of whether this was done at the study or outcome level), and how this information is to be used in any data synthesis.	6/7
Summary measures	13	State the principal summary measures (e.g., risk ratio, difference in means).	7
Synthesis of results	14	Describe the methods of handling data and combining results of studies, if done, including measures of consistency (e.g., I^2) for each meta-analysis.	7

For peer review only - http://bmjopen.bmj.com/site/about/guidelines.xhtml

PRISMA 2009 Checklist

Section/topic	#	Checklist item	Reported on page #
Risk of bias across studies	15	Specify any assessment of risk of bias that may affect the cumulative evidence (e.g., publication bias, selective reporting within studies).	7
Additional analyses	16	Describe methods of additional analyses (e.g., sensitivity or subgroup analyses, meta-regression), if done, indicating which were pre-specified.	N/A
**RESULTS**			
Study selection	17	Give numbers of studies screened, assessed for eligibility, and included in the review, with reasons for exclusions at each stage, ideally with a flow diagram.	7 and Figure 1
Study characteristics	18	For each study, present characteristics for which data were extracted (e.g., study size, PICOS, follow-up period) and provide the citations.	Table 1 and Table 2
Risk of bias within studies	19	Present data on risk of bias of each study and, if available, any outcome level assessment (see item 12).	Supplementary file 2
Results of individual studies	20	For all outcomes considered (benefits or harms), present, for each study: (a) simple summary data for each intervention group (b) effect estimates and confidence intervals, ideally with a forest plot.	8-14 and Figure 4
Synthesis of results	21	Present results of each meta-analysis done, including confidence intervals and measures of consistency.	8-14 and Figure 4
Risk of bias across studies	22	Present results of any assessment of risk of bias across studies (see Item 15).	N/A
Additional analysis	23	Give results of additional analyses, if done (e.g., sensitivity or subgroup analyses, meta-regression [see Item 16]).	N/A
**DISCUSSION**			
Summary of evidence	24	Summarize the main findings including the strength of evidence for each main outcome; consider their relevance to key groups (e.g., healthcare providers, users, and policy makers).	14/15
Limitations	25	Discuss limitations at study and outcome level (e.g., risk of bias), and at review-level (e.g., incomplete retrieval of identified research, reporting bias).	16/17
Conclusions	26	Provide a general interpretation of the results in the context of other evidence, and implications for future research.	15/16
**FUNDING**			
Funding	27	Describe sources of funding for the systematic review and other support (e.g., supply of data); role of funders for the systematic review.	18

From: Moher D, Liberati A, Tetzlaff J, Altman DG, The PRISMA Group (2009). Preferred Reporting Items for Systematic Reviews and Meta-Analyses: The PRISMA Statement. PLoS Med 6(6): e1000097.

For peer review only - http://bmjopen.bmj.com/site/about/guidelines.xhtml

PRISMA 2009 Checklist

doi:10.1371/journal.pmed1000097

For more information, visit: www.prisma-statement.org.

For peer review only

For peer review only - http://bmjopen.bmj.com/site/about/guidelines.xhtml
